# Recessive aminoacyl-tRNA synthetase disorders: lessons learned from *in vivo* disease models

**DOI:** 10.3389/fnins.2023.1182874

**Published:** 2023-05-09

**Authors:** Elizabeth Kalotay, Matthias Klugmann, Gary D. Housley, Dominik Fröhlich

**Affiliations:** ^1^Translational Neuroscience Facility and Department of Physiology, School of Biomedical Sciences, University of New South Wales, Sydney, NSW, Australia; ^2^Research Beyond Borders, Boehringer Ingelheim Pharma GmbH & Co. KG, Biberach an der Riss, Germany

**Keywords:** aminoacyl-tRNA synthetases, ARS1, ARS2, cytosolic ARS, mitochondrial ARS, recessive ARS mutations, animal models

## Abstract

Protein synthesis is a fundamental process that underpins almost every aspect of cellular functioning. Intriguingly, despite their common function, recessive mutations in aminoacyl-tRNA synthetases (ARSs), the family of enzymes that pair tRNA molecules with amino acids prior to translation on the ribosome, cause a diverse range of multi-system disorders that affect specific groups of tissues. Neurological development is impaired in most ARS-associated disorders. In addition to central nervous system defects, diseases caused by recessive mutations in cytosolic ARSs commonly affect the liver and lungs. Patients with biallelic mutations in mitochondrial ARSs often present with encephalopathies, with variable involvement of peripheral systems. Many of these disorders cause severe disability, and as understanding of their pathogenesis is currently limited, there are no effective treatments available. To address this, accurate *in vivo* models for most of the recessive ARS diseases are urgently needed. Here, we discuss approaches that have been taken to model recessive ARS diseases *in vivo*, highlighting some of the challenges that have arisen in this process, as well as key results obtained from these models. Further development and refinement of animal models is essential to facilitate a better understanding of the pathophysiology underlying recessive ARS diseases, and ultimately to enable development and testing of effective therapies.

## 1. Introduction

### 1.1. Canonical function of ARSs

Accurate and efficient protein synthesis is integral to cellular survival and functioning. At the ribosome, tRNA molecules act as ‘adaptors’ between mRNA codons and corresponding amino acids, enabling translation of mRNA sequences into polypeptides. Aminoacyl-tRNA synthetases (ARSs) are a family of ubiquitously expressed enzymes that play a crucial role in facilitating this process. ARSs are responsible for ligating tRNA molecules to their cognate amino acids, in a two-step reaction known as aminoacylation. In the first step, ATP is hydrolyzed within the active site of the ARS and an aminoacyl adenylate intermediate is formed (amino acid ‘activation’). The activated amino acid residue is then transferred to the 3′ acceptor end of a corresponding tRNA molecule (‘tRNA charging’), and AMP is released. Charged tRNA molecules are chaperoned to the ribosome by elongation factor thermally unstable (EF-Tu) in bacteria, or by eukaryotic translation elongation factor 1 alpha (eEF1A) in eukaryotes, where the tRNA anticodons are paired with complementary mRNA codons. ARSs are divided into two classes (I and II) based on structural differences in their catalytic domain, which influence their modes of substrate and ATP binding and the kinetics of the aminoacylation reaction (Ribas de Pouplana and Schimmel, 2001; Rubio Gomez and Ibba, 2020). These primary classes are further divided into three subclasses (a, b, and c), summarized in Table 1.

**Table 1:** The aminoacyl-tRNA synthetases.

Synthetase	Abbreviation	Gene(s)	Classification
Alanyl-tRNA synthetase	AlaRS	*AARS1*	Class IIa
Mitochondrial alanyl-tRNA synthetase	mt-AlaRS	*AARS2*	
Cysteinyl-tRNA synthetase	CysRS	*CARS1*	Class Ia
Mitochondrial cysteinyl-tRNA synthetase	mt-CysRS	*CARS2*	
Aspartyl-tRNA synthetase	AspRS	*DARS1*	Class IIb
Mitochondrial aspartyl-tRNA synthetase	mt-AspRS	*DARS2*	
Glutamyl-prolyl-tRNA synthetase	GluProRS	*EPRS1*	Class Ib (GluRS domain) Class IIa (ProRS domain)
Mitochondrial glutamyl-tRNA synthetase	mt-GluRS	*EARS2*	
Mitochondrial prolyl-tRNA synthetase	mt-ProRS	*PARS2*	
Phenylalanyl-tRNA synthetase	PheRS	*FARSA, FARSB*	Class IIc
Mitochondrial phenylalanyl-tRNA synthetase	mt-PheRS	*FARS2*	
Glycyl-tRNA synthetase	GlyRS	*GARS1*	Class IIa
Histidyl-tRNA synthetase	HisRS	*HARS1*	Class IIa
Mitochondrial histidyl-tRNA synthetase	mt-HisRS	*HARS2*	
Isoleucyl-tRNA synthetase	IleRS	*IARS1*	Class Ia
Mitochondrial isoleucyl-tRNA synthetase	mt-IleRS	*IARS2*	
Lysyl-tRNA synthetase	LysRS	*KARS1*	Class Ib and Class IIb
Leucyl-tRNA synthetase	LeuRS	*LARS1*	Class Ia
Mitochondrial leucyl-tRNA synthetase	mt-LeuRS	*LARS2*	
Methionyl-tRNA synthetase	MetRS	*MARS1*	Class Ia
Mitochondrial methionyl-tRNA synthetase	mt-MetRS	*MARS2*	
Asparaginyl-tRNA synthetase	AsnRS	*NARS1*	Class IIb
Mitochondrial asparaginyl-tRNA synthetase	mt-AsnRS	*NARS2*	
Glutaminyl-tRNA synthetase	GlnRS	*QARS1*	Class Ib
Mitochondrial glutaminyl-tRNA synthetase	mt-GlnRS	*QARS2*	
Arginyl-tRNA synthetase	ArgRS	*RARS1*	Class Ia
Mitochondrial arginyl-tRNA synthetase	mt-ArgRS	*RARS2*	
Seryl-tRNA synthetase	SerRS	*SARS1*	Class IIa
Mitochondrial seryl-tRNA synthetase	mt-SerRS	*SARS2*	
Threonyl-tRNA synthetase	ThrRS	*TARS1*	Class IIa
Mitochondrial threonyl-tRNA synthetase	mt-ThrRS	*TARS2*	
Valyl-tRNA synthetase	ValRS	*VARS1*	Class Ia
Mitochondrial valyl-tRNA synthetase	mt-ValRS	*VARS2*	
Tryptophanyl-tRNA synthetase	TrpRS	*WARS1*	Class Ic
Mitochondrial tryptophanyl-tRNA synthetase	mt-TrpRS	*WARS2*	
Tyrosyl-tRNA synthetase	TyrRS	*YARS1*	Class Ic
Mitochondrial tyrosyl-tRNA synthetase	mt-TyrRS	*YARS2*	

tRNA aminoacylation occurs separately in both the cytosol and mitochondria of cells. In eukaryotes, there are a total of 36 ARSs, comprising 17 that exclusively function within the cytosol, 17 within the mitochondria, and two bifunctional ARSs, which work in both compartments (Ognjenovic and Simonovic, 2018). Each ARS corresponds to one of the 20 proteinogenic amino acids, except for the fused cytosolic glutamyl-prolyl-tRNA synthetase (GluProRS), which catalyzes the aminoacylation reaction for both glutamic acid and proline in the cytosol. Furthermore, mitochondrial glutaminyl-tRNA synthetase (GlnRS) has not been identified in mammals, with aminoacylation of mammalian mitochondrial tRNA^Gln^ achieved through an indirect pathway involving transamidation of mischarged Glu-tRNA^Gln^ through the glutamyl-tRNA amidotransferase (Echevarría et al., 2014).

Each ARS is encoded by a single nuclear gene, apart from the cytosolic phenylalanyl-tRNA synthetase (PheRS), which has two separate genes encoding its alpha and beta subunits. For ARS gene nomenclature, a single letter is used to represent the relevant amino acid, followed by ‘*ARS1’* for the bifunctional and cytosolic ARSs, or ‘*ARS2*’ for their mitochondrial counterparts. For example, the aspartyl-tRNA synthetase (AspRS) encoded by *DARS1* is responsible for charging cytosolic tRNA^Asp^ molecules, while mitochondrial (mt)-AspRS encoded by *DARS2* performs the equivalent function in mitochondria. While most cytosolic and mitochondrial ARS pairs are encoded by separate genes, the cytosolic and mitochondrial forms of the bifunctional glycyl-tRNA synthetase (GlyRS) and lysyl-tRNA synthetase (LysRS) are encoded by single genes.

Accurate pairing of tRNAs with their cognate amino acids by ARSs is critical for implementation of the genetic code. ARSs bind specific tRNAs and amino acids based on their structural and physicochemical properties (Giege et al., 1998; Kaiser et al., 2020). However, strong structural similarities between certain amino acids can make them difficult to discriminate based on their binding site affinity alone. To help overcome this challenge, more than half of the ARSs (AlaRS, IleRS, LeuRS, LysRS, MetRS, PheRS, ProRS, SerRS, ThrRS, and ValRS) are capable of proofreading and hydrolyzing erroneously activated amino acids, either within the active site itself prior to tRNA charging, or after misacylation of tRNAs has occurred, using an appended editing domain (Perona and Gruic-Sovulj, 2014; Rubio Gomez and Ibba, 2020).

### 1.2. Secondary functions of ARSs

Throughout evolution, most ARSs have acquired diverse secondary functions. While some of these non-translational functions utilize the ARSs canonical substrate binding sites, or arise from alternative splicing (Lo et al., 2014), much of this functional expansion has been driven by the addition of novel protein domains (Guo and Schimmel, 2013; Pang et al., 2014). Examples of these functions include modulation of immune and inflammatory responses (Park et al., 2005; Ofir-Birin et al., 2013; Lee et al., 2016), regulation of angiogenesis (Mirando et al., 2014), as well as ageing and lifespan (Zhou et al., 2021). In mammals, eight of the cytosolic ARSs (ArgRS, AspRS, GlnRS, Glu-ProRS, IleRS, LeuRS, LysRS, and MetRS) come together with three Aminoacyl-tRNA synthetase Interacting Multifunctional Proteins (AIMP1, AIMP2, and AIMP3) to form a multi-synthetase complex (MSC). In response to specific molecular signals, individual ARSs and AIMPs within the complex undergo post-translational modifications and are released from the MSC to carry out their non-translational functions (Guo and Schimmel, 2013; Pang et al., 2014). Formation of the MSC appears dispensable for protein translation, suggesting that its primary role is to support or regulate secondary ARS functions (Kim et al., 2003; Cui et al., 2021).

### 1.3. ARSs and disease

Mutations in all ARS genes have been linked to diseases, the majority of which affect the nervous system. Despite their shared fundamental role in translation, the consequences of mutations in different ARS genes are remarkably heterogeneous, affecting distinct combinations of tissues. Genotype-phenotype correlations have been challenging to establish due to the broad disease spectrum associated with mutations in individual ARS genes, with reported cases of high intrafamilial variability (Riley et al., 2013; Ardissone et al., 2018; Muthiah et al., 2020; Ong et al., 2020; Ait-El-Mkadem Saadi et al., 2022). ARS mutations were first formally associated with human disease in 2003, with identification of dominant mutations in *GARS1* as the cause of Charcot-Marie-Tooth disease type 2D (CMT2D) and spinal muscular atrophy type V (SMAV; Antonellis et al., 2003). Dominant mutations in six other ARS1 genes (*AARS1, HARS1, MARS1, WARS1, SARS1,* and *YARS1*) have since also been linked to peripheral neuropathies (Wei et al., 2019). ARS2 genes have not yet been associated with dominant disease.

In contrast, recessive mutations in each of the cytosolic and mitochondrial ARSs have been connected to a broad range of multisystem diseases that often affect development of the central nervous system (CNS) (Ognjenovic and Simonovic, 2018; Fuchs et al., 2019). The recessive inheritance pattern of these disorders excludes haploinsufficiency as the disease mechanism. Interestingly, peripheral neuropathy is not a common clinical feature among patients with recessive ARS variants, suggesting that the pathophysiology underlying these two sets of diseases differs. This review focuses on the group of recessive ARS diseases, which broadly affect the CNS and other systems.

Key disease presentations associated with biallelic ARS1 and ARS2 mutations are summarized in Supplementary Tables 1, 2. Recessive ARS2 mutations most commonly cause encephalopathies, leukoencephalopathies, sensorineural hearing loss, and cardiovascular disease (Sissler et al., 2017; Zou et al., 2021; Del Greco and Antonellis, 2022). Diseases caused by recessive mutations in ARS1 genes often affect neurodevelopment and CNS myelination, but can also impact other systems, including the liver and lungs (Fuchs et al., 2019). Recently, novel variants in *AARS1* and *NARS1* have been associated with dominantly inherited CNS diseases (Sundal et al., 2019; Manole et al., 2020). These cases represent an exception to the generally recessive inheritance of ARS-related CNS pathologies; however, it is possible that dominant variants in other ARS genes may similarly be implicated in CNS or multisystem diseases in the future.

Recent studies have shown therapeutic benefits of bespoke interventions for individual recessive ARS1 disease cases. For example, multiple patients with recessive ARS1 diseases have experienced improvements following personalized amino acid and protein supplementation to compensate for reduced aminoacylation efficiency (Hadchouel et al., 2015; Kopajtich et al., 2016; Das et al., 2020; Lenz et al., 2020b; Kok et al., 2021). Improvements were also observed in a patient with biallelic *KARS2* mutations following multivitamin supplementation and adherence to a ketogenic diet (Murofushi et al., 2022), and in a patient with *DARS2* mutations following treatment with a derivative of succinic acid to support mitochondrial function (Bedova et al., 2020). Larger-scale clinical studies and further exploration of whether therapeutic benefits can similarly be achieved for other recessive ARS diseases are important next steps. Additionally, the development of curative treatments such as gene therapies that address the underlying disease causes are lacking and are urgently needed.

A question central to the pathophysiology of ARS dysfunction is how mutations in these essential, ubiquitously expressed enzymes preferentially affect specific tissues. Existing hypotheses include differing cellular energy and translational demands throughout development (Fuchs et al., 2019; González-Serrano et al., 2019), variations in tRNA and amino acid concentrations across different tissues (Kuo and Antonellis, 2020), and varied ARS expression levels across specific tissues and cell types (Fröhlich et al., 2017, 2018). Broader disruptions in translational efficiency and fidelity have been robustly linked to neurological disease, possibly reflecting a specialized need for rapid protein synthesis to support neuroplasticity, or difficulties in clearing misfolded proteins that arise from the unique morphology of neuronal cells (Kapur and Ackerman, 2018; Ognjenovic and Simonovic, 2018). Additionally, neurons are highly reliant on mitochondrial function to support axonal development, function, and regeneration (Devine and Kittler, 2018; Cheng et al., 2022), making them particularly sensitive to mitochondrial dysfunction that may be triggered by impaired mt-tRNA aminoacylation.

These explanations focus on loss of canonical ARS activity; however, it is possible that pathogenic ARS mutations also compromise secondary enzyme functions or have toxic gain-of-function effects, which may account for the heterogeneity of ARS disease phenotypes. An important and ongoing task is to identify the relative contributions of these mechanisms to the different diseases. A related point of importance is determining the extent to which their etiologies are distinct or shared. Better understanding of these key issues is essential for designing appropriately targeted therapies for this group of diseases.

## 2. Animal model systems for studying recessive ARS diseases

**Figure 1:**
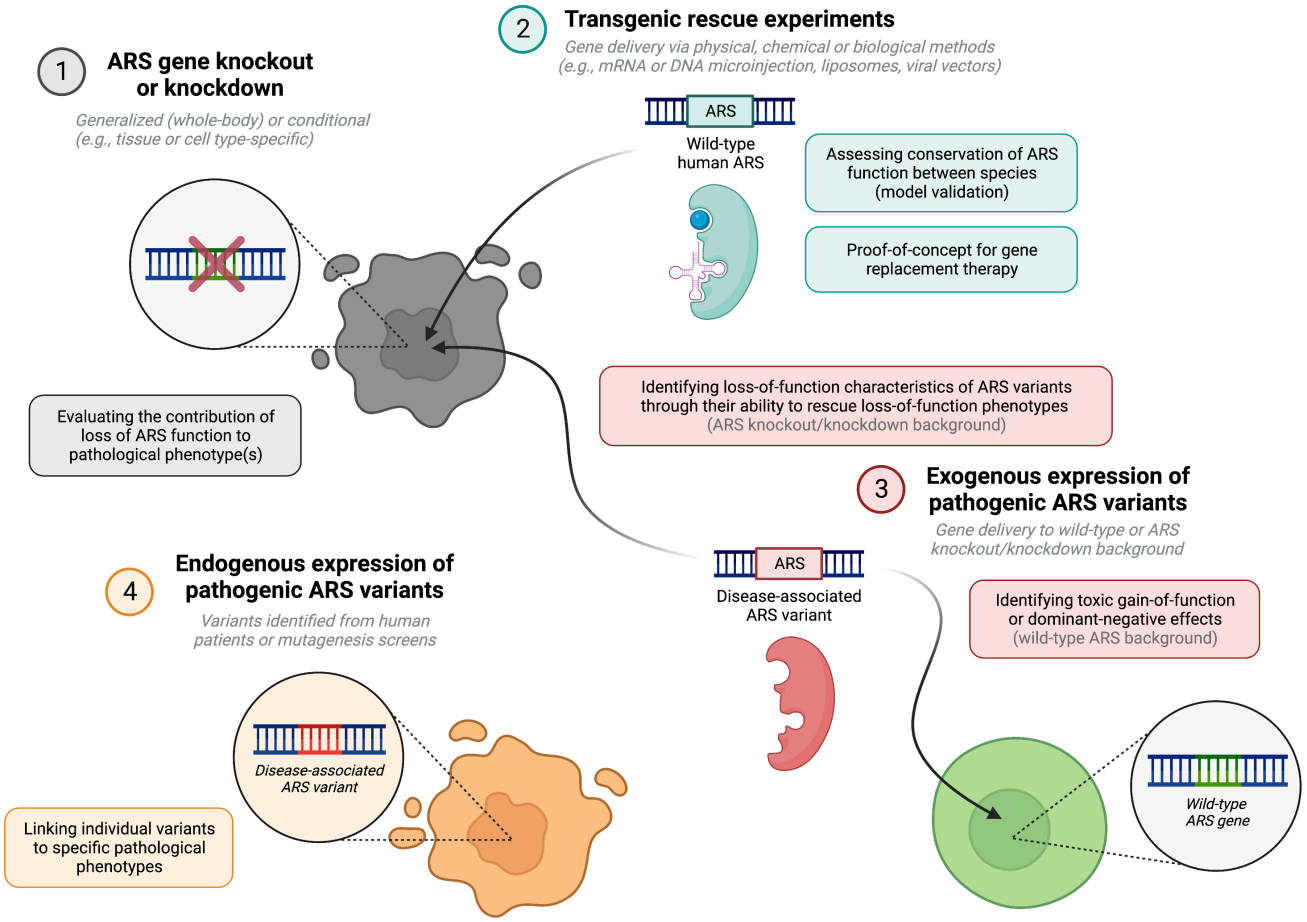
Key methodological approaches for modelling ARS pathology *in vivo*, and their potential theoretical relevance. This figure was created with BioRender.com.

**Figure 2:**
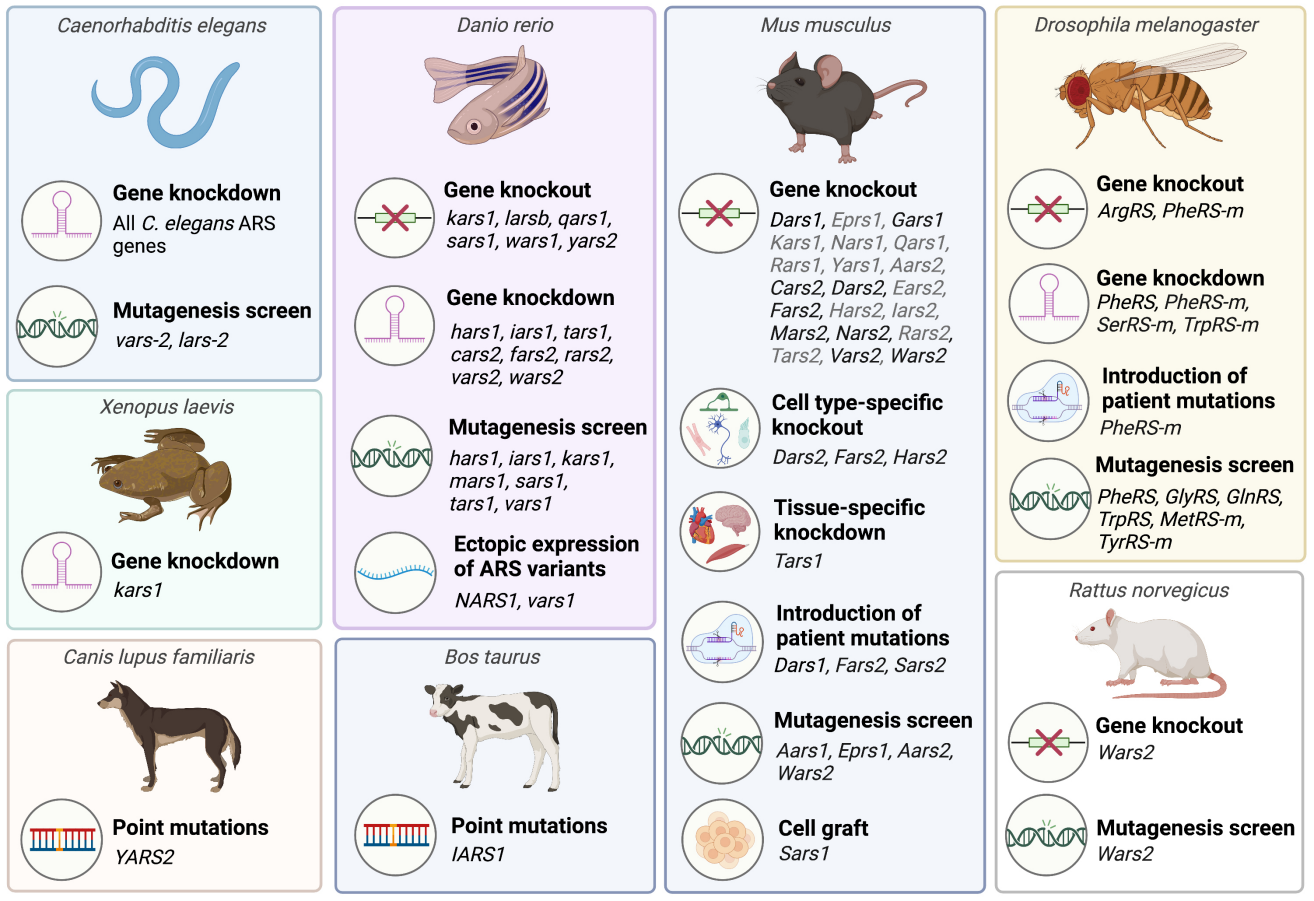
Overview of animal model systems employed to characterize aminoacyl-tRNA synthetases associated with recessive ARS disorders. A detailed list of animal studies including corresponding references is provided in Supplementary Tables 1, 2. Mouse ARS knockout models phenotyped by the International Mouse Phenotyping consortium (IMPC) that have not been published are listed in grey. This figure was created with BioRender.com.

The establishment of *in vivo* models for each of the recessive ARS diseases is crucial to advance the understanding of their pathogenesis and is a prerequisite to developing novel treatments. Protein structural analyses, *in vitro* aminoacylation assays, and yeast complementation assays have been widely used to investigate the effects of pathogenic ARS mutations on enzyme stability and activity (Meyer-Schuman and Antonellis, 2017; Oprescu et al., 2017), to help determine the extent to which loss of enzyme function may contribute to ARS disease pathogenesis. However, these assays are of limited value for establishing the contributions of toxic gain-of-function effects or loss of secondary functions, particularly where non-canonical functions remain unknown. Additionally, the performance of mutant ARSs in aminoacylation assays is not always reflective of their pathogenicity *in vivo* (van Berge et al., 2013; González-Serrano et al., 2019; Schuch et al., 2021). Despite the high costs and ethical considerations associated with conducting animal research, functional *in vivo* studies are vital as they facilitate comprehensive investigation into the downstream consequences of pathogenic ARS mutations within the context of an entire organism (Figure 1). While several studies have modelled ARS dysfunction *in vivo* (Figure 2; Supplementary Tables 1, 2), accurate pre-clinical models are still lacking for most ARS-associated diseases, particularly in mammals. This class of diseases has proven to be particularly challenging to model *in vivo* due to species-dependent penetrance and severity of the underlying ARS mutations, the essential and ubiquitous function of ARSs in protein synthesis, and the lack of redundancy amongst ARSs.

Equivalence of ARS function between species can be tested through overexpression of the wildtype human gene in knockout or knockdown animals, where ability of the wildtype human ARS to compensate for loss of the endogenous ARS is indicative of functional conservation. Once this has been established, rescue experiments using mutant ARSs on a loss-of-function background can inform on residual enzymatic activity of specific ARS variants. Additionally, overexpression of pathogenic mutant human genes on a wild-type or heterozygous gene knockout background can inform on toxic gain-of-function or dominant-negative effects (Oprescu et al., 2017).

Phenotyping of ARS knockout or knockdown animals can reveal aspects of ARS-associated diseases which arise from loss of enzyme function. Precision gene editing tools such as CRISPR/Cas9 can also be used to introduce specific disease-causing point mutations into endogenous ARS genes to replicate the disease. Both gene knockout and transgene expression can be spatially and temporally controlled, using cell type-specific promoters and inducible site-specific recombinase systems, including Cre-loxP and Flp-FRT technologies (Branda and Dymecki, 2004). Viral vectors may be used to deliver transgenes or to silence genes *via* RNA interference (RNAi) in distinct cell populations at predetermined time points, including specific cell populations within the nervous system (von Jonquieres et al., 2013, 2016). Combining these techniques enables substantial flexibility in gene manipulation.

### 2.1. Species for modelling ARS diseases

Due to their essential role in protein synthesis, ARSs have been highly conserved throughout evolution, allowing for translational studies across multiple species (Figure 2).

Invertebrate species such as *Caenorhabditis* (*C.*) *elegans* and *Drosophila melanogaster* provide relatively low cost, high-throughput platforms for assessing the downstream consequences of pathogenic ARS mutations, as well as for screening the therapeutic potential of candidate drugs and interventions. Many fundamental physiological processes are conserved between humans, *C. elegans* and *Drosophila*, and both species have successfully been used to model multiple neurodegenerative diseases (Lu and Vogel, 2009; Caldwell et al., 2020). These invertebrate transgenic animal models facilitate analysis of survival, morphological defects, as well as behavioral abnormalities using basic tests of sensory and motor function. A particular advantage of *C. elegans* is their transparency, which enables longitudinal visualization of nervous system development or degeneration.

Vertebrate species that have been used to model ARS diseases include zebrafish (*Danio rerio*), *Xenopus laevis,* and mouse (*Mus musculus*). Zebrafish have high fecundity, develop rapidly, and are less expensive to maintain than mice, making them well suited for early investigations into the *in vivo* effects of ARS gene manipulations. The external fertilization of zebrafish means that they are easily accessible for early genetic manipulation through microinjections of expression plasmids, transcription activator-like effector nuclease (TALEN) mRNA, or CRISPR/Cas9 reagents into early (e.g., one-cell stage) embryos (Köster and Sassen, 2015). Additionally, the transparency of zebrafish during development, and the recent creation of a zebrafish mutant that retains transparency in adulthood enables observation of zebrafish neuromorphology, neuronal activity, and cellular processes such as axonal transport *in vivo* (Stewart et al., 2014; Antinucci and Hindges, 2016; Atkins et al., 2022). Zebrafish brain structures are readily imaged *in situ* (Stewart et al., 2014), and their chemical permeability makes them well suited for rapidly screening water-soluble chemical compounds for their therapeutic efficacy and toxicity (Veldman and Lin, 2008). Unlike invertebrate models, it is possible to investigate the effects of ARS mutations on myelination in zebrafish (Preston and Macklin, 2015). The experimental paradigms available for the behavioral phenotyping of zebrafish have also expanded in recent years, to include tests of social and anxiety-related behaviors, in addition to basic tests of locomotor function (Stewart et al., 2014).

Use of *Xenopus* to study ARS disease has been less common than zebrafish, however, this species shares many of the advantages of zebrafish for disease modelling, including rapid production of large numbers of offspring, and the ability to precisely manipulate gene expression during the early stages of development through intracellular microinjections. The *Xenopus* model also enables selective unilateral manipulation of gene expression in the embryo through one-sided injections at the 2-cell stage of development, with the contralateral side of the body serving as an internal control (Lasser et al., 2019). Embryonic development of *Xenopus* has been well characterized, and this model system has been widely used to study abnormal development of multiple systems including the nervous system (Borodinsky, 2017; Lee-Liu et al., 2017; Choi et al., 2022) and the heart (Hoppler and Conlon, 2020).

As mammals, mice have a higher level of anatomical and physiological similarity to humans than zebrafish, *Xenopus,* or invertebrate species. It is therefore important that results from non-mammalian studies are validated in mammals. Mice are the most widely used species for modelling human diseases, and many genetically identical inbred strains of mice are readily available for use in studies of gene function, including existing transgenic mouse models for a range of diseases (Amos-Landgraf et al., 2022). Many tools for gene manipulation and other experimental techniques, including comprehensive behavioral test batteries have also been extensively validated in this species (Sukoff Rizzo and Crawley, 2017).

Online databases are available for each of these species, providing comprehensive genomic data, protocols, and information on existing transgenic lines and reagents to facilitate research with these model organisms (Consortium A.O.G.R, 2022).

### 2.2. ARS knockout and knockdown studies

Recessive ARS diseases are thought to be primarily caused by impairments in tRNA aminoacylation (Meyer-Schuman and Antonellis, 2017). *In vitro* studies with patient cells, yeast complementation assays, and aminoacylation assays are consistent with a loss of canonical enzyme function, and the pathogenicity of ARS variants can generally be predicted by these assays (Oprescu et al., 2017). Initial attempts to model recessive ARS diseases have therefore focused on ARS knockout or knockdown. Heterozygous ARS knockout in mice does not lead to nervous system dysfunction, whereas homozygous knockout of cytosolic or mitochondrial ARS genes is embryonically lethal (Seburn et al., 2006; Dogan et al., 2014; Dickinson et al., 2016; Akaike et al., 2017; Fröhlich et al., 2017; Cheong et al., 2020; Hines et al., 2021; Xu et al., 2021; Chen et al., 2022). While this precludes the use of ARS knockout mice to model recessive ARS disease, it demonstrates that cytosolic and mitochondrial ARSs are non-redundant and are each essential for embryonic development and survival. Consistent with this, human patients may be homozygous for hypomorphic alleles, or compound heterozygous for a functional null allele and a hypomorphic allele, but never carry two functional null alleles. This suggests that the threshold at which loss of ARS function triggers major disease phenotypes lies below 50% of wildtype enzyme function. While this poses a challenge for accurately modelling this group of diseases, it also implies that restoration of enzyme function to 50% may be sufficient to confer significant therapeutic benefits.

While homozygous ARS knockouts in mice are not viable, numerous studies have successfully characterized the consequences of ARS1 and ARS2 knockout and knockdown in *C. elegans*, *Drosophila,* and zebrafish, since these organisms are oviparous and undergo a larval stage in which early development is supported by maternal mRNA. These models recapitulate key aspects of the human diseases, supporting the loss-of-function hypothesis and enabling investigation into the downstream consequences of ARS knockout. In addition to general ARS gene knockout, cell type-specific ARS knockout models have so far been produced in *Drosophila* and mice. Summaries of ARS1 and ARS2 knockout models that have been generated to date are provided in Supplementary Tables 1, 2.

#### 2.2.1. Common phenotypic features of ARS knockout and knockdown

While ARSs are ubiquitously expressed, their expression is enriched within specific tissues during development, particularly within the CNS, eyes, muscles, and digestive system (Wang Z et al., 2018; Siekierska et al., 2019; Lin et al., 2021). Individual ARSs also appear to be differentially expressed across individual organs and cell types, possibly reflecting a higher requirement for incorporation of their corresponding amino acids in cell type-specific proteins, or cell type-specific secondary functions (Zhang et al., 2014; Fröhlich et al., 2017, 2018; Wang Z et al., 2018; Siekierska et al., 2019; Inoue et al., 2021; Lin et al., 2021, 2022).

Consistent with the comparatively high levels of ARS expression in the nervous system, multiple ARS1 knockout studies, primarily conducted in zebrafish, have demonstrated that loss of ARS1 gene function profoundly impairs nervous system development. Zebrafish knockout models have so far been created for *kars1, lars1, qars1*, *vars1,* and *wars1* (Zhang et al., 2014; Wang Z et al., 2018; Siekierska et al., 2019; Inoue et al., 2021; Lin et al., 2021, 2022). Complete loss of any of these enzymes is lethal during larval development. Up to 2 days post fertilization (dpf), levels of ARS mRNA in knockout animals are comparable to wildtypes due to contribution of maternal mRNA. From 3 dpf, ARS expression drops off and there is rapid functional deterioration, including severe morphological defects predominantly affecting the brain, eyes, and heart, as well as locomotor dysfunction. Death of ARS1 knockout zebrafish occurs within 8-12 dpf. Knockdown of *iars1* and cytosolic *hars1* expression in zebrafish similarly affect brain and eye development in a dose-dependent manner (Kopajtich et al., 2016; Waldron et al., 2019). In line with these zebrafish results, morpholino knockdown of endogenous *kars1* expression in *Xenopus* leads to abnormal development of the head and eyes, and a lack of myelination in the ventral side of the brain (Itoh et al., 2019). Brain and eye development were more severely affected in *Xenopus* following selective knockdown of the cytosolic isoform of *kars1* compared to its mitochondrial isoform (Itoh et al., 2019). The impact of ARS1 knockout and knockdown on the eyes and brain is consistent with the frequent occurrence of microcephaly, brain atrophy, and visual impairments amongst patients with biallelic ARS1 mutations. Given that these defects are common across the different ARS1 knockout models, they are likely to result from loss of canonical ARS1 function and impaired translation.

Studies examining loss of ARS2 function in *Drosophila* and zebrafish have similarly demonstrated highly detrimental effects on early development and survival. The effects of gene knockout or knockdown have directly been tested for mt-CysRS, mt-PheRS, mt-ArgRS, mt-SerRS, mt-ThrRS, mt-ValRS, mt-TrpRS, and mt-TyrRS. As was observed for ARS1 knockdown animals, severity of the phenotype corresponds to the level of gene knockdown, with complete ARS2 knockout being lethal during early development in both zebrafish and *Drosophila* (Kasher et al., 2011; Maffezzini et al., 2019; Fan et al., 2021; Jin et al., 2021; Chen et al., 2022; Mo et al., 2023). Central nervous system development and cardiac function appear to be particularly vulnerable to loss of ARS2 function, likely due to their high metabolic demands. Morpholino knockdown of endogenous *rars2* in zebrafish leads to underdevelopment and structural abnormalities within the brain, mirroring morphological defects induced by knockdown of the *tsen54* gene that encodes tRNA-splicing endonuclease complex, which like *RARS2,* has been linked to pontocerebellar hypoplasia (PCH) in human patients (Kasher et al., 2011). Transient knockdown of *vars2* expression during zebrafish embryonic development causes cerebral oedema (Kayvanpour et al., 2022), and homozygous knockout of *yars2* disrupts development of the retina (Jin et al., 2021). Knockdown of *vars2* in zebrafish additionally causes heart failure (Kayvanpour et al., 2022), and *wars2* knockdown causes pericardial oedema, impaired angiogenesis, and cardiac contractile failure (Wang et al., 2016).

#### 2.2.2. General (whole-body) ARS knockout and knockdown recapitulate key disease phenotypes

Despite some overlapping features, the consequences of knocking out different ARS genes vary, consistent with the heterogeneity of diseases caused by mutations in these genes (Supplementary Tables 1, 2).

In humans, homozygous mutations in *KARS1,* which encodes bifunctional LysRS, are associated with particularly heterogeneous clinical manifestations, with no clear genotype–phenotype correlation (Ardissone et al., 2018). The most common disease features include non-syndromic hearing loss, neurodevelopmental delay, leukoencephalopathy, microcephaly, and seizures (Lin et al., 2021). In zebrafish, knockout of *kars1* produces phenotypic features reminiscent of the human pathology; specifically, knockout larvae exhibit sensorineural hearing loss, visual impairments, and susceptibility to seizures (Lin et al., 2021). Patients with *KARS1* mutations have also been reported to exhibit neuromuscular dysfunction, a phenotype that is recapitulated in *kars1* knockout animals.

Mutations in *LARS1* have been associated with infantile liver failure syndrome type 1 (ILFS1) (Lenz et al., 2020a). The most common symptoms reported in the literature so far include growth abnormalities, neurodevelopmental delay, recurrent elevation of liver transaminases, liver failure, failure to thrive, seizures, blood abnormalities (microcytic anemia), abnormality of the musculature (hypotonia), and hypoalbuminemia (Lenz et al., 2020a). In zebrafish, cytosolic LeuRS is encoded by two genes, *larsa* and *larsb*, with *larsb* being more homologous to the human *LARS1* gene. Knockout of *larsb* (*larsb^−/−^*) in zebrafish most severely affected the liver, with a marked decrease in liver size starting from 3 dpf (Wang Z et al., 2018; Inoue et al., 2021). Like ILFS1 patients, the *larsb^−/−^* zebrafish also exhibited anemia and microcephaly (Inoue et al., 2021).

Similarly, mutations in *VARS1* cause Neurodevelopmental Disorder with Microcephaly, Seizures, and Cortical Atrophy (NDMSCA; Siekierska et al., 2019). Consistent with the occurrence of epilepsy in NDMSCA patients, *vars1* knockout zebrafish exhibit seizure activity from 5 dpf (Siekierska et al., 2019). Key clinical features observed in patients with biallelic *WARS1* mutations are recapitulated in zebrafish *wars1* knockouts, including facial abnormalities, muscular defects, and hearing deficits (Lin et al., 2022). Knockdown of *cars2* in zebrafish has been shown to impair migration of gonadotropin-releasing hormone (GnRH) neurons during embryonic development, consistent with the association between *CARS2* mutations and hypogonadotropic hypogonadism in humans (Wang et al., 2015).

Dysfunction of the heart and central nervous system in ARS2 knockout models reflects the common involvement of these systems in recessive ARS2 diseases. For example, biallelic missense mutations in *FARS2* have been linked to hereditary spastic paraplegia (Vantroys et al., 2017). In keeping with this phenotype, morpholino knockdown of *fars2* expression in zebrafish has been shown to cause impaired motor axon development and significant locomotor dysfunction (Chen et al., 2022). In another study, a distinctive phenotype of *yars2* knockout zebrafish was abnormal development of the retina (Jin et al., 2021). While biallelic *YARS2* mutations have not yet been reported to cause vision impairments, mutations in *YARS2* have been found to interact with mutations in a mitochondrial gene linked to Leber’s hereditary optic neuropathy, worsening cellular respiration in patient cells, and exacerbating associated visual impairments (Jiang et al., 2016).

The reproduction of characteristic pathological features in ARS1 and ARS2 knockout and knockdown animals supports the hypothesis that a loss-of-function underlies the pathogenesis of these disorders. It is important to note that existing studies may have preferentially screened for abnormalities that align with the clinical presentations of patients, which can make it difficult to determine whether certain phenotypes are uniquely triggered by knockdown or knockout of individual ARSs. Application of standardized procedures for comprehensive phenotyping of novel ARS disease models are required for such comparisons. Phenotypes observed in animal models not yet reported in patient cases may lead to the discovery of novel disease markers that are relevant in the clinical setting.

#### 2.2.3. Conditional (cell type-specific) knockout and knockdown

Conditional knockout of ARS function enables investigation into how dysfunction in specific cell types contribute to the ARS disease phenotypes. For example, neuronal knockout of *PheRS-m* in *Drosophila* causes a milder level of developmental delay compared to *Drosophila* with generalized *PheRS-m* knockout, indicating that loss of mitochondrial PheRS function within the nervous system partially contributes to this phenotype (Fan et al., 2021). Neuronal *PheRS-m* knockout flies also exhibit susceptibility to seizures, mirroring the epileptic phenotype of patients with *fars2* deficiency (Fan et al., 2021). Selective RNAi knockdown of *SerRS-m* in either the muscle or neurons of *Drosophila* caused a significant reduction in lifespan, with muscle specific *SerRS-m* depletion also impairing locomotor function (Guitart et al., 2013). Understanding which cell populations are most affected by loss of ARS function can guide treatment strategies such as gene replacement, by identifying target cell types for therapeutic transgene expression.

Mammalian cell type-specific ARS1 knockout models have not yet been characterized, however, results obtained from conditional knockouts of *Dars2* in mice suggest that this may similarly be a promising strategy for circumventing the embryonic lethality of generalized ARS1 knockout in mice. Oligodendrocyte- and neuron-specific *Dars1* knockout mouse models are currently under investigation in the authors’ lab. Mutations in the mitochondrial *DARS2* gene cause Leukoencephalopathy with Brainstem and Spinal cord involvement and Lactate elevation (LBSL) (Scheper et al., 2007; Muthiah et al., 2020). Despite LBSL being a white matter disease, a direct comparison between the effects of oligodendrocyte and neuronal *Dars2* knockout in mice demonstrates that neurons are more susceptible to loss of mt-AspRS function than oligodendrocytes (Aradjanski et al., 2017). While oligodendrocyte-specific *Dars2* knockout from 4 weeks of age does not impact oligodendrocyte survival or CNS myelination, selective loss of *Dars2* function in CamKIIα-expressing forebrain cortical and hippocampal neurons from postnatal day (P) 14 causes severe mitochondrial dysfunction and neuroinflammation, leading to widespread neuronal apoptosis by 6 months of age (Aradjanski et al., 2017). As noted by the authors of this study, oligodendrocytes may be more sensitive to loss of *Dars2* at an earlier timepoint, during myelination of the developing brain.

A subsequent neuronal *Dars2* knockout model created using a different CamKIIα-Cre line similarly showed substantial hippocampal and cortical neurodegeneration, as well as neuroinflammation, activation of immune signaling, and induction of the integrated stress response (ISR) (Nemeth et al., 2020). Interestingly, some differences were observed between the two CamKIIα-Cre *Dars2* knockout models. Where the mice characterized by Aradjanski et al. (2017) exhibited significant mitochondrial dysfunction and low weight gain relative to controls, mice characterized by Nemeth et al. (2020) had an increased number of mitochondria within axons of the corpus callosum, which retained their normal morphology. These mice also had higher body mass than controls. Behaviorally, mice characterized in the first study showed a progressive decline in motor function, including tremors, ataxia, and kyphosis, whereas the second group of *Dars2* knockout mice showed a progressive increase in locomotor activity relative to control animals. The different results produced in these two studies, despite using the same promoter for Cre-mediated *Dars2* knockout, highlight the sensitivity of phenotypical outcomes to factors such as genetic background or environmental conditions. These factors are therefore important to take into consideration when evaluating and comparing results obtained from different studies and research groups.

In another LBSL model, knockout of *Dars2* in mouse Purkinje neurons between P5 and P7 similarly resulted in mitochondrial dysfunction, neuroinflammation, impaired cellular connectivity, and substantial loss of Purkinje cells, highlighting the importance of *Dars2* for supporting neuronal function (Rumyantseva et al., 2020). Notably, even among neuronal cell populations, there are differences in vulnerability to loss of *Dars2,* with cortical neurons appearing more sensitive to *Dars2* depletion than hippocampal neurons (Aradjanski et al., 2017). Together, these conditional knockout studies suggest that neuronal dysfunction likely contributes to LBSL pathology and have provided mammalian models of neuronal mt-AspRS deficiency, which can be employed in drug efficacy studies to test novel LBSL treatment strategies.

The tissue-specific consequences of loss of mt-AspRS function were similarly highlighted in another conditional knockout mouse model, where *Dars2* expression was knocked out in cardiac and skeletal muscle by embryonic day (E) 15.5, with skeletal muscle showing a greater capacity to compensate for the consequent loss of mitochondrial function than cardiomyocytes (Dogan et al., 2014). Mitochondrial stress responses, which were not observed in skeletal muscle even at the peak of mitochondrial dysfunction at 6 weeks of age, were initiated in cardiomyocytes as early as at 1 week of age, prior to detectable mitochondrial respiratory chain deficiency. These early stress responses included initiation of the mitochondrial unfolded protein response (UPR) and increased expression of fibroblast growth factor 21 (FGF21), leading to upregulation of peroxisome proliferator-activated receptor gamma coactivator 1-alpha (PGC1-α), which stimulates mitochondrial biogenesis. Increased FGF21 expression in *Dars2* knockout mice led to systemic metabolic changes including decreased fat mass and lower blood glucose levels.

Mutations in *FARS2* are similarly linked to neurological disease including infantile-onset mitochondrial encephalopathy and spastic paraplegia (Almannai et al., 2018). Neuronal *Fars2* knockout (*Fars2*-cKO) was recently tested in mice, with gene knockout in nestin-expressing cells initiated by E11 (Chen et al., 2022). *Fars2*-cKO mice exhibit severe structural abnormalities within the brain during subsequent embryonic development, including enlargement of the ventricles and cortical degeneration, consistent with the occurrence of brain atrophy in multiple human patients with biallelic *FARS2* mutations (Vantroys et al., 2017; Chen et al., 2022). Analysis of brain tissue from *Fars2*-cKO embryos revealed reduced translation and assembly of mitochondrial respiratory chain subunit complexes, mitochondrial degeneration, and progressive apoptotic cell death within the cortex. *Fars2*-cKO mice are stillborn or die shortly after birth, indicating that mt-PheRS function in neurons is essential to support embryonic development. It is possible that viable *Fars2*-cKO offspring may be produced if neuronal *Fars2* knockout is initiated at a later timepoint.

Hearing loss is a common feature of recessive diseases caused by mutations in several ARSs, including *EPRS1*, *HARS1*, *HARS2, IARS2, KARS1, LARS2* and NARS2 (Pierce et al., 2011; Puffenberger et al., 2012; Pierce et al., 2013; Santos-Cortez et al., 2013; Simon et al., 2015; Soldà et al., 2016; Sun et al., 2019; Jin et al., 2022). A recent study investigated the consequences of selective *Hars2* knockout (*Hars2-*cKO) in the inner hair cells (IHC) and outer hair cells (OHC) of the cochlea in mice using the Cre-loxP system, with Cre recombinase expression driven by the promoter for the hair cell-selective transcription factor *Gfi1* (Xu et al., 2021). Phenotypically, *Hars2-*cKO mice exhibited hearing loss from P30, despite displaying no significant loss of hair cells until ~P45, after which loss of both IHCs and OHCs rapidly progressed. Increasing numbers of morphologically abnormal mitochondria were observed in the hair cells of *Hars2-*cKO mice from P14, with a concomitant accumulation of reactive oxygen species (ROS) and mitochondrial apoptosis. Whole-cell patch clamping of IHCs revealed impaired synaptic transmission in IHCs due to a reduction in calcium influx, likely underlying the early hearing loss in *Hars2-*cKO mice. Deficits in synaptic transmission preceding the progressive loss of hair cells may similarly be caused by loss-of-function of other ARSs that have been linked to hearing loss; a possibility that can be explored in future studies through the generation of appropriate conditional knockout models.

#### 2.2.4. Rescue experiments

Rescue experiments with wildtype human ARS mRNA consistently improve the phenotypes of knockout animals, demonstrating conservation of ARS function and validating their use for modelling ARS diseases. As monogenic diseases, recessive ARS disorders may be amenable to gene replacement therapy; a treatment strategy that can be explored using ARS knockout models.

ARS knockout models may also be used for screening pathogenic ARS variants, as multiple studies across *Drosophila, Xenopus*, and zebrafish have demonstrated the ability of wildtype, but not mutant human ARS mRNA to rescue knockout phenotypes. A recent study demonstrated that the phenotype caused by loss of *vars1* in zebrafish could be rescued effectively by wildtype, but not by mutant human *VARS1* mRNA (Siekierska et al., 2019). Zebrafish *wars1* and *wars2* knockouts could be rescued with wildtype human *WARS1* and *WARS2* mRNA, respectively, however mutant *WARS1* mRNA failed to rescue *wars1* knockout zebrafish (Bögershausen et al., 2022; Lin et al., 2022). Similarly, developmental defects induced by morpholino knockdown of *kars1* in *Xenopus* could partially be rescued through delivery of wildtype, but not pathogenic human *KARS1* mRNA (Itoh et al., 2019). LysRS is known to have a phosphorylation-dependent secondary function where it is released from the MSC, translocates to the nucleus, and activates the microphthalmia-associated transcription factor (MITF) to regulate the immune response in mast cells (Ofir-Birin et al., 2013). Interestingly, blocking nuclear localization of the pathogenic human *KARS1* variant resulted in rescue of the knockdown phenotype similar to the wildtype *KARS1* variant (Itoh et al., 2019), possibly by preventing a toxic gain-of-function within the nucleus, or increasing the bioavailability of LysRS outside the nucleus to perform the canonical aminoacylation function.

Many recessive ARS disease patients are compound heterozygous carriers of two different variants. In this instance, rescue experiments can inform on the contribution of individual variants to specific pathological hallmarks. In a study where multiple pathological *wars1* variants were tested for their ability to rescue zebrafish *wars1* knockout, different variants failed to rescue different aspects of the knockout phenotype, such as hearing deficits or head and eye abnormalities, seemingly consistent with clinical features exhibited by patients with the respective mutations (Lin et al., 2022).

Likewise, expression of a human *FARS2* variant linked to epileptic encephalopathy exacerbated the seizure phenotype of *PheRS-m* knockout *Drosophila*, while expression of a pathological variant associated with spastic paraplegia impaired climbing activity (Fan et al., 2021). Worsening of the *PheRS-m* knockout phenotypes upon expression of mutant alleles implies that these variants produce toxic gain-of-function effects. The distinct phenotypes produced by mutations in the same gene suggest that separate protein functions are affected by the different variants; a possibility that may be further investigated using these models.

### 2.3. Expression of editing-defective ARS variants

Impairments in the ability of ARSs to recognize their appropriate substrates or to rectify errors that occur during tRNA aminoacylation can lead to the misincorporation of amino acids into proteins as they are synthesized. Protein mistranslation in the cytosol exerts stress on the endoplasmic reticulum, which can in turn lead to activation of the UPR (Walter and Ron, 2011). Prolonged activation of the UPR as a result of the ongoing production of misfolded proteins ultimately leads to the induction of pro-apoptotic signaling pathways and cell death (Lin et al., 2008). Mistranslation of proteins within the mitochondria can trigger the mitochondrial UPR, which, like the cytosolic UPR, can be protective in the short-term, but damaging if persistently activated (Shpilka and Haynes, 2018).

Defective proteins produced due to mistranslation can have additional deleterious effects. For example, expression of an editing defective ValRS variant in zebrafish caused DNA damage, widespread cell death, and a reduction in lifespan, hypothesized to be a consequence of malfunctioning DNA replication and repair proteins (Song et al., 2016).

Studies conducted in both *Drosophila* and mice have indicated that the nervous system is particularly affected by errors in protein translation arising from compromised fidelity in tRNA aminoacylation. In *Drosophila,* complete disruption of the proofreading mechanisms of PheRS is lethal during development (Lu et al., 2014). A partial deficit of PheRS editing activity in *Drosophila* results in growth defects, locomotor deficits, and accelerated ageing. Selective expression of editing-defective PheRS variants in the eye causes significant morphological defects of the retina, highlighting the reliance of neural cells on accurate tRNA aminoacylation for normal development. The same study demonstrated that this defect is not restricted to developmental stages, as the expression of proofreading deficient PheRS in adult *Drosophila* also causes neurodegeneration (Lu et al., 2014).

Similar outcomes have been observed in mice expressing editing-defective AlaRS variants. Homozygous expression of an aminoacylation-capable but editing-defective *Aars1* variant (p.Ala734Glu) in mice (*Aars1^A734E/A734E^*) causes accumulation of misfolded proteins and progressive degeneration of Purkinje neurons (Lee et al., 2006). In vertebrates, proofreading is carried out by the protein ANKRD16, in addition to the proofreading performed by the AlaRS editing domain (Vo et al., 2018). Deletion of the *Ankrd16* gene in *Aars1^A734E/A734E^* mice is embryonically lethal, and conditional knockout of *Ankrd16* in specific neuronal cell populations of *Aars1*^A734E/A734E^ mice results in widespread neurodegeneration within the corresponding cell populations, indicating that vulnerability of neurons to tRNA^Ala^ mischarging is not exclusive to Purkinje neurons (Vo et al., 2018). In a separate study, homozygous expression of another *Aars1* variant with more severely compromised editing activity (*Aars1^C723A/C723A^*) was embryonically lethal, but expression of this variant *in trans* to the p.Ala734Glu allele (*Aars1^C723A/A734E^*) resulted in mice that exhibit loss of cardiomyocytes in addition to Purkinje cell degeneration, revealing the sensitivity of another cell population to errors in protein synthesis (Liu et al., 2014).

*In vivo* consequences of mitochondrial mistranslation due to an ARS editing deficiency have so far been investigated for mt-AlaRS. Two editing domain mutations in *Aars2* (*Aars2^V760E^,* equivalent to the *Aars1^A734E^* variant, and *Aars2^C749A^*) have been shown to increase the misacylation of tRNA^Ala^ with serine *in vitro*, without significantly compromising mt-AlaRS aminoacylation activity (Hilander et al., 2018). Mice heterozygous for either of these variants are phenotypically unaffected, and embryonic fibroblasts cultured from mice expressing the more severe *Aars2^C749A^* variant are capable of sustaining normal mitochondrial protein synthesis in serine-enriched media, demonstrating the ability of the wildtype allele to compensate for the editing defect (Hilander et al., 2018). In contrast, homozygosity for either of these variants in mice is lethal during embryonic development, highlighting the necessity of mt-AlaRS proofreading activity for supporting mitochondrial function. Whether editing deficiencies in other mitochondrial ARSs are equally deleterious is yet to be tested *in vivo*.

### 2.4. Expression of human patient ARS variants

Another approach for creating ARS disease models is to introduce specific mutations, which cause the respective disease in human patients, into the endogenous ARS gene of the model species. As previously mentioned, it is possible for different mutations within a single ARS gene to cause different diseases, possibly by perturbing residues that are important for separate functions of the enzyme (Sissler et al., 2017). Comparison between the downstream effects of individual pathogenic ARS variants may provide insights into the specific pathways that contribute to distinct disease phenotypes.

Biallelic mutations in the *DARS1* gene cause the leukodystrophy Hypomyelination with Brainstem and Spinal cord involvement and Leg spasticity (HBSL; Taft et al., 2013; Wolf et al., 2015; Muthiah et al., 2020). The introduction of HBSL-causing mutations [p.Asp367Tyr (Fröhlich et al., 2020), p.Met256Leu (Klugmann et al., 2022)] into the mouse *Dars1* gene were recently evaluated as candidate models for HBSL. In contrast to HBSL patients, mice homozygously expressing these variants (*Dars1^D367Y/D367Y^*, *Dars1^M256L/M256L^*) do not show any severe nervous system dysfunction (Fröhlich et al., 2020; Klugmann et al., 2022). Neurological abnormalities were only observed upon crossing these homozygous *Dars1* mutant mice with heterozygous *Dars1*-null mice (*Dars1^D367Y/−^, Dars1^M256L/−^*), indicating that both variants are hypomorphic, despite an apparent increase in aminoacylation activity for the *Dars1^D367Y^* variant when assayed *in vitro* (Fröhlich et al., 2020). Specifically, compound heterozygous *Dars1^D367Y/−^* mice have severe developmental deficits, with only ~18% of *Dars1^D267Y/−^* offspring surviving to birth. All surviving *Dars1^D367Y/−^* mice are significantly smaller and lighter than control littermates, and hydrocephalus and eye defects are common among *Dars1^D367Y/−^* mice (Fröhlich et al., 2020). Resembling aspects of HBSL pathology, motor dysfunction and myelin defects, primarily within the spinal cord, were observed in aged *Dars1^D367Y/−^* mice. Similarly, *Dars1^M256L/−^* mice have a reduced birth rate and exhibit developmental delay (Klugmann et al., 2022). Eye abnormalities and hydrocephalus occur in ~20% of *Dars1^M256L/−^* mice. *Dars1^M256L/−^* mice do not develop significant motor deficits, however, vacuolization of the spinal cord white matter was also observed in aged mice. Interestingly, *Dars1^M256L/−^* mice show pronounced metabolic changes, with an overall reduction in body fat mass compared to wildtype littermates. A similar trend was observed in *Dars1^D367Y/−^* mice, albeit to a lesser extent (Fröhlich et al., 2020). It is unclear whether similar metabolic changes occur in HBSL patients, however, based on the results from these mouse studies, this warrants investigation.

Despite the comparatively mild phenotype shown by mice expressing HBSL-causing *Dars1* mutations, two mouse models with knock-in of pathogenic ARS2 variants were more severely affected than their human counterparts. Homozygous expression of a *Fars2* mutation (p.Asp142Tyr), which severely compromises mt-PheRS aminoacylation activity and causes hereditary spastic paraplegia in humans, is embryonically lethal in mice during early gestation, at a similar timepoint to *Fars2* knockout mice (Yang et al., 2016; Chen et al., 2022). Similarly, homozygous or compound heterozygous expression of human disease-causing *Sars2* variants was embryonically lethal in mice (Yu et al., 2022). The specific factors underlying the variation in phenotype severity between mouse and human carriers of isogenic ARS mutations are not known, however, these species differences should be considered when evaluating novel ARS disease models.

Ectopic expression of a pathogenic ARS variant on a wildtype background can be informative about whether the variant exerts a toxic gain-of-function or a dominant-negative effect on wildtype enzyme function *in vivo*. For example, expression of mutant human *KARS1* mRNA in *Xenopus* was sufficient to cause head and eye defects resembling animals with morpholino knockdown of endogenous *kars1* expression, suggesting that these disease-causing variants interfere with an essential function of wildtype *kars1* (Itoh et al., 2019). Similarly, injection of a human *NARS1* variant mRNA into wildtype zebrafish led to abnormalities in embryonic development, causing dose-dependent cyclopia and defects in gastrulation (Manole et al., 2020). As previously mentioned, *NARS1* is unusual among the ARSs in that it has been associated with severe neurodevelopmental disorders that have both dominant and recessive modes of inheritance (Manole et al., 2020), suggesting that some pathogenic *NARS1* variants might exert toxic gain-of-function or dominant-negative effects.

### 2.5. Pathogenic ARS variants identified through genetic screens

Additional ARS mutants with phenotypes relevant to the associated recessive diseases have been identified *via* forward genetic screens. For example, two missense variants in the *Drosophila MetRS-m* gene (p.Val42Asp., p.Ser224Leu) were identified in a genetic screen (mosaic FLP-FRT eye screen) for mutations causing a neurodegenerative phenotype in the eye (Bayat et al., 2012). Cells from *MetRS-m* mutant *Drosophila* recapitulated pathological features of human patient cells, including mitochondrial dysfunction, increased ROS, and impaired cell proliferation. Abnormal lipid deposits were observed in neural tissue of *MetRS-m* mutant *Drosophila*, possibly indicative of metabolic dysfunction. *MetRS-m* mutant flies also exhibited progressive myofibril degeneration in their flight muscles, rendering them incapable of flight. Interestingly, despite apoptosis being a recurrent feature in numerous ARS loss-of-function models, apoptosis was not observed in the brain or non-neural tissue tested in the study. The *MetRS-m* mutant phenotype could be completely rescued through overexpression of *Drosophila* or human wildtype mt-MetRS, indicating conservation of function between the species and confirming that the phenotype was due to loss of mt-MetRS function. The pathological phenotype of *MetRS-m* mutant *Drosophila* could also be attenuated through administration of antioxidants. Similar phenotypic improvements following antioxidant administration were reported in a *SerRS-m* deficient *Drosophila* model (Guitart et al., 2013). It is yet to be determined whether this approach is similarly effective in other models of ARS2 dysfunction and in human patients.

Novel pathogenic ARS variants identified in mutagenesis screens can elicit additional pathological phenotypes not yet documented in human patients. N-ethyl-N-nitrosourea (ENU) mutagenesis has been used to induce biallelic hypomorphic point mutations within the mouse *Wars2* gene, with the p.Val117Leu variant (*Wars2^V117L/V117L^*) identified in a screen for mutations causing age-related disease phenotypes (Potter et al., 2016). Mutations in *Wars2* cause a range of clinical presentations, including neurodevelopmental disorder with abnormal movements, lactic acidosis with or without seizures (NEMMLAS), and infantile-onset Parkinsonism (Theisen et al., 2017; Wortmann et al., 2017; Burke et al., 2018; Virdee et al., 2019). Expression of the p.Val117Leu variant results in a *Wars2* splicing defect, reducing mt-TrpRS levels in mice (Agnew et al., 2018). While *Wars2^V117L/−^* mice are not viable, *Wars2^V117L/V117L^* mice exhibit reduced adiposity, progressive hearing loss, and hypertrophic cardiomyopathy (Agnew et al., 2018; Mušo et al., 2022). No overt brain or neurological abnormalities were observed in *Wars2^V117L/V117L^* mice (Agnew et al., 2018). Mutations in *WARS2* have not been directly linked to hearing loss in human patients, however, sensorineural hearing loss is caused by mutations in other ARS2 genes (Sissler et al., 2017; Del Greco and Antonellis, 2022). While cardiomyopathy has previously been documented in a NEMMLAS patient (Wortmann et al., 2017), other aspects of the mouse phenotype differ from the typical clinical presentation of human patients with recessive *WARS2* mutations. The equivalent mutation has not yet been observed in human patients, and it is likely that different point mutations in *WARS2* affect different aspects of enzyme function, given the heterogeneity of clinical presentations associated with recessive ARS disorders. Novel pathological phenotypes associated with ARS dysfunction in animal models may be observed in future disease cases, as more patients are identified with new variants.

Results from a forward genetic screen for gene variants affecting the dendritic and axonal morphology of *Drosophila* olfactory projection neurons suggest that the function of ARSs in protein translation is required to support proper development and maintenance of these structures (Chihara et al., 2007). Here, a loss-of-function mutation within the predicted catalytic domain of the *Drosophila GlyRS* gene caused defective arborization of axonal and dendritic terminals. Normal neuronal morphology could be fully restored through transgenic expression of wildtype GlyRS. Given that GlyRS is a bifunctional enzyme, the authors sought to differentiate between the contributions of cytosolic and mitochondrial protein translation to this phenotype. Loss-of-function mutations introduced into TrpRS and GlnRS, which act exclusively in the cytosol, produced similar morphological defects, while selective disruption of mitochondrial protein synthesis did not affect the development of axonal and dendritic terminals. *Drosophila* with compromised mitochondrial protein translation instead showed a progressive reduction in dendritic density, indicating the importance of mitochondrial translation for maintenance of dendritic terminals rather than their initial development. Abnormalities in dendritic and axonal morphology have been linked to several neurological disorders (Kulkarni and Firestein, 2012; Van Battum et al., 2015), and may also contribute to ARS disease pathogenesis.

### 2.6. Animal models to investigate secondary ARS functions

Non-canonical enzyme functions are an important consideration when generating and interpreting novel ARS disease models. A number of non-canonical ARS functions have been demonstrated *in vivo*, including regulation of the immune response (Nayak et al., 2021), myogenic differentiation (Dai et al., 2021), autophagy (Han et al., 2012), cell proliferation, and tumorigenesis (Ho et al., 2021). Understanding secondary ARS functions can enable better anticipation of cellular dysfunction that may result from pathogenic variants, guiding identification of relevant disease biomarkers and selection of appropriate treatment strategies.

Multiple ARSs have been shown to influence angiogenesis, dysregulation of which is associated with several pathologies including inflammatory diseases and cancer (Yoo and Kwon, 2013; Mirando et al., 2014; Jeong et al., 2021). Neurovascular communication is also recognized to play an important role in neurogenesis and CNS development (Peguera et al., 2021; Vogenstahl et al., 2022). Forward genetic screens conducted in zebrafish have linked *iars1, sars1,* and *tars1* variants to defects in angiogenesis during development, which can also be induced through morpholino knockdown of the respective genes (Jin et al., 2007; Fukui et al., 2009; Herzog et al., 2009; Cao et al., 2016; Castranova et al., 2016; Wang K et al., 2018), but not through general inhibition of protein synthesis (Fukui et al., 2009; Herzog et al., 2009; Xu et al., 2012; Cao et al., 2016). Pro- and anti-angiogenic effects of TyrRS and TrpRS splice variants *via* the phosphoinositide 3-kinase (PI3K) and extracellular signal-regulated kinase (ERK) pathways have also been demonstrated *in vivo* in rats and rhesus monkeys (Wang et al., 2016; Zeng et al., 2016). Additionally, a *Wars2* variant identified through linkage analysis impairs cardiac angiogenesis in rats, and *wars2* knockdown in zebrafish causes similar vascularization defects (Wang et al., 2016). GluProRS activity has also been linked to angiogenesis *in vitro* (Mirando et al., 2014), but has not yet been investigated *in vivo*.

Studies investigating the angiogenic dysregulation caused by loss of SerRS and ThrRS function indicate that ARSs influence angiogenesis through multiple independent pathways. Under normal conditions, SerRS can translocate to the nucleus *via* a nuclear localization signal (NLS). In the nucleus, SerRS regulates angiogenesis through repression of vascular endothelial growth factor A (VEGFA) expression by antagonizing the transcription factor C-Myc and recruiting the histone deacetylase Sirtuin 2 (Xu et al., 2012; Shi et al., 2014). *In vivo* experiments in zebrafish and mice have demonstrated that phosphorylation of SerRS, triggered by hypoxia, inactivates this VEGFA repression function (Shi et al., 2020). Excessive vessel branching induced by *sars1* knockdown in zebrafish could be rescued through injection of wildtype human or zebrafish SerRS mRNA, as well as by expression of an aminoacylation-deficient *sars1* variant, but not a phospho-mimetic *sars1* variant, demonstrating that regulation of angiogenesis by SerRS is separate from its canonical function (Shi et al., 2020).

Zebrafish with loss-of-function mutations or knockdown of *tars1* similarly exhibit excessive branching of vessels, also caused by increased VEGFA signaling (Castranova et al., 2016; Zhang et al., 2021). Interestingly, despite their common link to VEGFA regulation, wildtype *sars1* mRNA is not able to rescue the abnormal angiogenesis phenotype observed in *tars1* mutants, indicating that these ARSs modulate VEGFA activity *via* different pathways (Cao et al., 2016). Hyperactivation of VEGFA in *tars1* mutant zebrafish occurs downstream of the amino acid response (AAR), *via* activation of general control nondepressible 2 (GCN2), phosphorylation of eukaryotic initiation factor 2 alpha (eIF2α), and upregulation of activating transcription factor 4 (Atf4) (Zhang et al., 2021). The abnormal phenotype of *tars1*-deficient zebrafish can be rescued through expression of wildtype zebrafish or human ThrRS mRNA, but not by aminoacylation-deficient *tars1* variants, suggesting that in contrast to SerRS, ThrRS aminoacylation function may be required to support angiogenesis (Castranova et al., 2016; Zhang et al., 2021).

Metabolic changes have also been observed in multiple animal models of ARS dysfunction. In the case of mt-ARSs, this can result from loss of canonical enzyme function. Metabolic changes can occur downstream of mitochondrial stress *via* FGF21 activation, as has been demonstrated for both *Dars2* and *Wars2* loss-of-function mouse models (Dogan et al., 2014; Mušo et al., 2022). In addition to FGF21 activation, upregulation of growth/differentiation factor 15 (GD15) was observed in *Wars2* mutant mice and hypothesized to cause their decreased food intake (Mušo et al., 2022). Cytosolic ARS function has also been linked to metabolism. For example, GluProRS has been shown to have a phosphorylation-dependent secondary function in adipogenesis, with expression of a phospho-defective *Eprs1* variant in mice causing reduced adiposity, higher energy expenditure, improved glucose homeostasis, and a longer lifespan (Arif et al., 2017). Mice with loss-of-function mutations in *Dars1* similarly show a reduction in adiposity (Fröhlich et al., 2020; Klugmann et al., 2022), however, the underlying mechanisms have not been investigated, and it is therefore unknown whether this metabolic change occurs as a consequence of disrupted AspRS canonical function, or a yet unidentified secondary function.

### 2.7. Identification of potential treatment strategies

Determining which pathways are disrupted downstream of pathological ARS mutations can help to identify suitable treatment targets. Existing animal models of ARS diseases have elucidated numerous pathways perturbed by loss of ARS function that directly contribute to the corresponding pathophysiology.

Dysregulation of the cell cycle has been implicated in a number of ARS disease models. Tumor protein 53 (p53) plays a regulatory role in several cellular processes, including DNA repair, cell cycle arrest, and apoptosis (Chen, 2016). In a zebrafish LysRS loss-of-function model, tissue-specific apoptosis was found to result from pathological p53 upregulation, with inhibition of p53 signaling rescuing multiple aspects of the abnormal phenotype (Lin et al., 2021). Interestingly, p53 upregulation was also observed in zebrafish expressing an editing-defective ValRS variant, however, in this model p53 activation was a protective response to DNA damage, with p53 knockdown in *vars1* mutants exacerbating developmental abnormalities and shortening their lifespan (Song et al., 2016). In zebrafish, knockdown of *hars1* has been shown to cause cell cycle arrest and apoptosis, which was most pronounced in the nervous system (Waldron et al., 2019). This was found to occur independently of p53 activation, instead resulting from induction of the AAR preventing normal accumulation of cyclin D1 (CCND1), thus causing stalling of the cell cycle and triggering apoptosis. Overexpression of CCND1 in *hars1* knockdown zebrafish rescued the abnormal phenotype, confirming the causal role of cell cycle arrest in the observed neurodevelopmental defects and identifying a potential treatment target (Waldron et al., 2019). As induction of the AAR is likely to be a common consequence of defective tRNA charging by mutant ARSs, cell cycle dysregulation may similarly contribute to the pathogenesis of other ARS disorders.

LeuRS zebrafish models have demonstrated that disruption of secondary LeuRS function directly contributes to the pathological phenotype, representing an actionable therapeutic target for *LARS1* associated liver disease. LeuRS can act as an amino acid sensor for intracellular leucine concentration resulting in activation of mammalian target of rapamycin complex 1 (mTORC1) signaling, which inhibits autophagy (Han et al., 2012). Loss of this secondary LeuRS function results in pathological induction of autophagy, which is evident in *larsb* knockout zebrafish, particularly within the liver, the central nervous system, and in skeletal muscle (Inoue et al., 2021). Both morpholino-mediated and pharmacological inhibition of autophagy using bafilomycin partially rescued the aberrant phenotype of *larsb* knockout zebrafish and increased liver size and survival rates (Inoue et al., 2021). Another study reported rescue of liver failure in *larsb* knockout zebrafish following treatment with an mTORC1 morpholino or the mTORC1 inhibitor rapamycin (Wang Z et al., 2018). However, these results could not be reproduced in a subsequent study, making the therapeutic benefit of rapamycin for LysRS dysfunction inconclusive (Inoue et al., 2021).

Loss of mt-ARS function has been shown to cause mitochondrial dysfunction in several ARS disease models, commonly accompanied by a pathological build-up of ROS, which contributes to a number of disorders including neurodegeneration, cardiovascular disease, and cancer (Bayat et al., 2012; Guitart et al., 2013; Forrester et al., 2018; Kausar et al., 2018; Xu et al., 2021). Counteracting the accumulation of ROS through administration of antioxidants has been shown to improve the phenotypes of *MetRS-m* mutant and *SerRS-m* knockdown *Drosophila* (Bayat et al., 2012; Guitart et al., 2013), but has yet to be tested in other *in vivo* models of mt-ARS dysfunction. Finally, a recent study that conducted RNA sequencing for *PheRS-m* knockout *Drosophila* revealed downregulation of the hedgehog signaling pathway during development, with pharmacological agonism of this pathway achieving partial rescue of the abnormal phenotype (Mo et al., 2023). Whether hedgehog signaling is similarly perturbed by loss-of-function of other mt-ARSs and could thus represent a generalizable treatment target is yet to be established.

As monogenic diseases, ARS disorders are ideal candidates to be treated by gene therapy, addressing the root cause of the diseases with the potential for permanent alleviation of symptoms. Therapeutic genetic material can be delivered *in vivo* using viral vectors, with adeno-associated viruses (AAVs) showing particular promise for clinical applications due to their low immunogenicity and high transduction efficiency (Naso et al., 2017; Deverman et al., 2018). Using this approach, diseases caused by loss-of-function gene variants can be treated by delivering functional gene copies directly to the affected cell populations. Alternatively, toxic gain-of-function variants may be targeted through AAV-mediated RNAi. In the case of recessive ARS diseases, specific cell populations are more affected than others by ARS dysfunction, and directing transgene expression to these cells is likely to result in optimal treatment outcomes. Cell type-specific targeting of AAV-mediated transgene expression can be achieved through selection of appropriate capsid serotypes and promoter sequences (von Jonquieres et al., 2013, 2016; Naso et al., 2017; Fröhlich et al., 2022). Development of suitable animal models and identification of the cell populations primarily affected by ARS mutations will guide vector design and enable pre-clinical efficacy studies to establish the optimal timepoint of intervention, dosage, and route of administration. Since most recessive ARS disorders are associated with CNS abnormalities, the biggest challenge for virus-mediated gene therapies will be to achieve sufficient gene delivery to the CNS while avoiding detrimental side effects in the periphery. CNS delivery can be achieved by selecting CNS-targeted, liver de-targeted capsids for systemic injections, or through more direct delivery routes, including intracranial, intracerebroventricular, intracisternal, or intrathecal injections, which provide better CNS distribution, reduced impact on peripheral organs, and lower immunogenicity (Kells et al., 2015; Hudry and Vandenberghe, 2019; Marchi et al., 2022).

### 2.8. Limitations and considerations for the use of animal models

Despite the apparent benefit of *in vivo* ARS disease models, it is important to recognize that animal models have inherent limitations, and that the validity and suitability of each model must be evaluated on a case-by-case basis to avoid the inappropriate or unnecessary use of animals. Due to species-specific differences, animal models can only provide approximations of the corresponding human conditions. The three important criteria for the validity of an animal disease model are whether the mechanisms underlying the observed phenotype are analogous to the disease pathomechanism (construct validity), how closely the model’s phenotype resembles key clinical features of the human condition (face validity), and whether modifiers of the model’s phenotype (e.g., treatments) produce similar effects in human patients (predictive validity) (Tadenev and Burgess, 2019).

The known causes of ARS diseases, and high ARS gene conservation across species means that it is possible to closely replicate the genetic basis of these diseases in animal studies, maximizing their construct validity. However, due to various interspecies differences, high construct validity does not necessarily coincide with high face validity, as has already been observed in mouse lines that are either non-viable or phenotypically unaffected despite expressing ARS variants found in human disease patients (Fröhlich et al., 2020; Chen et al., 2022; Klugmann et al., 2022; Yu et al., 2022). Conversely, some animal models produced through alternative approaches including gene knockdown or knockout have reproduced key phenotypic features of the corresponding human ARS diseases. While these models do not replicate the exact genetic defects found in human patients, they directly link loss of ARS function to pathological phenotypes and may be utilized to further investigate the corresponding disease mechanisms and to test novel therapies. In some cases, novel phenotypes observed in animal models may be clinically relevant, particularly for rare diseases where the full clinical spectrum is not yet known. Ultimately, predictive validity of an animal model is important for preclinical testing of candidate treatments.

In light of these limitations and trade-offs, it will be important to complement results from animal studies with evidence obtained using additional methods, such as induced pluripotent stem cell (iPSC) models, which can provide insight into aberrant cellular mechanisms and pathways underlying the human pathophysiology.

## 3. Conclusion

The list of diseases associated with recessive mutations in ARSs is extensive and continuing to expand. The severity of many of these diseases, and the current lack of curative treatments, are strong motivators to better understand their underlying pathophysiology. Several animal models have so far provided key insights into ARS disease mechanisms, potential treatment targets, and methodological considerations for creating future models. Knockout of ARS function *in vivo* has demonstrated the necessity of each of these enzymes to support embryonic development and survival. Animals with compromised ARS function, through gene knockdown or expression of deleterious mutations, recapitulate key features of the respective human diseases, supporting the hypothesis that loss-of-function is a major contributor to their pathogenesis. Additionally, some disease-associated ARS variants have been shown to exert toxic gain-of-function effects, which contribute to the pathological phenotypes observed *in vivo*. The heterogeneity of clinical symptoms exhibited by ARS disease patients, even between individuals harboring different mutations of the same gene, has been reproduced in animal disease models and likely reflects the impact of these variants on different ARS protein domains and functions. Determining the relative contributions of compromised canonical enzyme function, disrupted secondary functions, and toxic gain-of-function effects to the pathogenesis of ARS diseases is a key issue of interest that may be explored further using these models. Pre-clinical models demonstrating substantive rescue of human recessive ARS gene mutation-related biochemical, structural, and behavioral deficits are needed to drive translation of novel treatment strategies to clinical trials, as the critical bridge towards addressing the unmet clinical needs arising from ARS deficits.

## Author contributions

EK and DF led the project and the manuscript production. MK and GH contributed to the manuscript preparation. All authors contributed to the article and approved the submitted version.

## Funding

This work was funded by the European Leukodystrophy Association (ELA 2018-014I2) and the Australian Government Medical Research Future Fund (Leukodystrophy Flagship – Massimo’s Mission; MRFF-ARLKO). EK was supported by an Australian Government Research Training Program (RTP) Scholarship.

## Conflict of interest

MK was employed by the company Boehringer Ingelheim Pharma GmbH & Co. KG.

The remaining authors declare that the research was conducted in the absence of any commercial or financial relationships that could be construed as a potential conflict of interest.

## Publisher’s note

All claims expressed in this article are solely those of the authors and do not necessarily represent those of their affiliated organizations, or those of the publisher, the editors and the reviewers. Any product that may be evaluated in this article, or claim that may be made by its manufacturer, is not guaranteed or endorsed by the publisher.

## References

[ref1] Agnew T. Goldsworthy M. Aguilar C. Morgan A. Simon M. Hilton H.. (2018). A Wars2 mutant mouse model displays OXPHOS deficiencies and activation of tissue-specific stress response pathways. Cell Rep. 25, 3315–3328.e3316. doi: 10.1016/j.celrep.2018.11.080 30566859 PMC6315286

[ref2] Ait-El-Mkadem Saadi S. Kaphan E. Morales Jaurrieta A. Fragaki K. Chaussenot A. Bannwarth S.. (2022). Splicing variants in NARS2 are associated with milder phenotypes and intra-familial variability. Eur. J. Med. Genet. 65:104643. doi: 10.1016/j.ejmg.2022.104643 36252909

[ref3] Akaike T. Ida T. Wei F. Y. Nishida M. Kumagai Y. Alam M. M.. (2017). Cysteinyl-tRNA synthetase governs cysteine polysulfidation and mitochondrial bioenergetics. Nat. Commun. 8:1177. doi: 10.1038/s41467-017-01311-y 29079736 PMC5660078

[ref4] Almannai M. Wang J. Dai H. El-Hattab A. W. Faqeih E. A. Saleh M. A.. (2018). FARS2 deficiency; new cases, review of clinical, biochemical, and molecular spectra, and variants interpretation based on structural, functional, and evolutionary significance. Mol. Genet. Metab. 125, 281–291. doi: 10.1016/j.ymgme.2018.07.014 30177229

[ref5] Amos-Landgraf J. Franklin C. Godfrey V. Grieder F. Grimsrud K. Korf I.. (2022). The mutant mouse resource and research center (MMRRC): the NIH-supported National Public Repository and distribution archive of mutant mouse models in the USA. Mamm. Genome 33, 203–212. doi: 10.1007/s00335-021-09894-0 34313795 PMC8314026

[ref6] Antinucci P. Hindges R. (2016). A crystal-clear zebrafish for in vivo imaging. Sci. Rep. 6:29490. doi: 10.1038/srep29490 27381182 PMC4933947

[ref7] Antonellis A. Ellsworth R. E. Sambuughin N. Puls I. Abel A. Lee-Lin S. Q.. (2003). Glycyl tRNA synthetase mutations in Charcot-Marie-tooth disease type 2D and distal spinal muscular atrophy type V. Am. J. Hum. Genet. 72, 1293–1299. doi: 10.1086/375039 12690580 PMC1180282

[ref8] Aradjanski M. Dogan S. A. Lotter S. Wang S. Hermans S. Wibom R.. (2017). DARS2 protects against neuroinflammation and apoptotic neuronal loss, but is dispensable for myelin producing cells. Hum. Mol. Genet. 26, 4181–4189. doi: 10.1093/hmg/ddx307 28985337

[ref9] Ardissone A. Tonduti D. Legati A. Lamantea E. Barone R. Dorboz I.. (2018). KARS-related diseases: progressive leukoencephalopathy with brainstem and spinal cord calcifications as new phenotype and a review of literature. Orphanet J. Rare Dis. 13:45. doi: 10.1186/s13023-018-0788-4 29615062 PMC5883414

[ref10] Arif A. Terenzi F. Potdar A. A. Jia J. Sacks J. China A.. (2017). EPRS is a critical mTORC1-S6K1 effector that influences adiposity in mice. Nature 542, 357–361. doi: 10.1038/nature21380 28178239 PMC5480610

[ref11] Atkins M. Hazan J. Fassier C. (2022). In vivo live imaging of axonal transport in developing zebrafish axons. Methods Mol. Biol. 2431, 325–350. doi: 10.1007/978-1-0716-1990-2_17 35412285

[ref12] Bayat V. Thiffault I. Jaiswal M. Tetreault M. Donti T. Sasarman F.. (2012). Mutations in the mitochondrial methionyl-tRNA synthetase cause a neurodegenerative phenotype in flies and a recessive ataxia (ARSAL) in humans. PLoS Biol. 10:e1001288. doi: 10.1371/journal.pbio.1001288 22448145 PMC3308940

[ref13] Bedova M. A. Ilves A. G. Prakhova L. N. Savintseva Z. I. Chernysheva E. M. (2020). Two clinical cases of LBSL: diagnostic problems and possible therapeutic approaches. Ann. Indian Acad. Neurol. 23, 825–826. doi: 10.4103/aian.AIAN_430_19 33688143 PMC7900743

[ref14] Bögershausen N. Krawczyk H. E. Jamra R. A. Lin S. J. Yigit G. Hüning I.. (2022). WARS1 and SARS1: two tRNA synthetases implicated in autosomal recessive microcephaly. Hum. Mutat. 43, 1454–1471. doi: 10.1002/humu.24430 35790048

[ref15] Borodinsky L. N. (2017). *Xenopus laevis* as a model organism for the study of spinal cord formation, development, function and regeneration. Front Neural Circuits 11:90. doi: 10.3389/fncir.2017.00090 29218002 PMC5704749

[ref16] Branda C. S. Dymecki S. M. (2004). Talking about a revolution: the impact of site-specific recombinases on genetic analyses in mice. Dev. Cell 6, 7–28. doi: 10.1016/s1534-5807(03)00399-x 14723844

[ref17] Burke E. A. Frucht S. J. Thompson K. Wolfe L. A. Yokoyama T. Bertoni M.. (2018). Biallelic mutations in mitochondrial tryptophanyl-tRNA synthetase cause levodopa-responsive infantile-onset parkinsonism. Clin. Genet. 93, 712–718. doi: 10.1111/cge.13172 29120065 PMC5828974

[ref18] Caldwell K. A. Willicott C. W. Caldwell G. A. (2020). Modeling neurodegeneration in *Caenorhabditis elegans*. Dis. Model. Mech. 13:dmm046110. doi: 10.1242/dmm.046110 33106318 PMC7648605

[ref19] Cao Z. Wang H. Mao X. Luo L. (2016). Noncanonical function of threonyl-tRNA synthetase regulates vascular development in zebrafish. Biochem. Biophys. Res. Commun. 473, 67–72. doi: 10.1016/j.bbrc.2016.03.051 26993167

[ref20] Castranova D. Davis A. E. Lo B. D. Miller M. F. Paukstelis P. J. Swift M. R.. (2016). Aminoacyl-transfer RNA Synthetase deficiency promotes angiogenesis via the unfolded protein response pathway. Arterioscler. Thromb. Vasc. Biol. 36, 655–662. doi: 10.1161/atvbaha.115.307087 26821951 PMC4808418

[ref21] Chen J. (2016). The cell-cycle arrest and apoptotic functions of p53 in tumor initiation and progression. Cold Spring Harb. Perspect. Med. 6:a026104. doi: 10.1101/cshperspect.a026104 26931810 PMC4772082

[ref22] Chen X. Liu F. Li B. Wang Y. Yuan L. Yin A.. (2022). Neuropathy-associated Fars2 deficiency affects neuronal development and potentiates neuronal apoptosis by impairing mitochondrial function. Cell Biosci. 12:103. doi: 10.1186/s13578-022-00838-y 35794642 PMC9258231

[ref23] Cheng X. T. Huang N. Sheng Z. H. (2022). Programming axonal mitochondrial maintenance and bioenergetics in neurodegeneration and regeneration. Neuron 110, 1899–1923. doi: 10.1016/j.neuron.2022.03.015 35429433 PMC9233091

[ref24] Cheong A. Archambault D. Degani R. Iverson E. Tremblay K. D. Mager J. (2020). Nuclear-encoded mitochondrial ribosomal proteins are required to initiate gastrulation. Development 147:dev188714. doi: 10.1242/dev.188714 32376682 PMC7272334

[ref25] Chihara T. Luginbuhl D. Luo L. (2007). Cytoplasmic and mitochondrial protein translation in axonal and dendritic terminal arborization. Nat. Neurosci. 10, 828–837. doi: 10.1038/nn1910 17529987

[ref26] Choi B. Kim H. Jang J. Park S. Jung H. (2022). Development and degeneration of retinal ganglion cell axons in *Xenopus tropicalis*. Mol. Cells 45, 846–854. doi: 10.14348/molcells.2022.0081 36380734 PMC9676988

[ref27] Consortium A.O.G.R (2022). Harmonizing model organism data in the Alliance of genome resources. Genetics 220:iyac022. doi: 10.1093/genetics/iyac022 35380658 PMC8982023

[ref28] Cui H. Kapur M. Diedrich J. K. Yates J. R. Ackerman S. L. Schimmel P. (2021). Regulation of ex-translational activities is the primary function of the multi-tRNA synthetase complex. Nucleic Acids Res. 49, 3603–3616. doi: 10.1093/nar/gkaa1183 33341895 PMC8053116

[ref29] Dai C. Reyes-Ordoñez A. You J. S. Chen J. (2021). A non-translational role of threonyl-tRNA synthetase in regulating JNK signaling during myogenic differentiation. FASEB J. 35:e21948. doi: 10.1096/fj.202101094R 34569098 PMC10226677

[ref30] Das A. Fröhlich D. Achanta L. B. Rowlands B. D. Housley G. D. Klugmann M.. (2020). L-aspartate, L-ornithine and L-ornithine-L-aspartate (LOLA) and their impact on brain energy metabolism. Neurochem. Res. 45, 1438–1450. doi: 10.1007/s11064-020-03044-9 32424601

[ref31] Del Greco C. Antonellis A. (2022). The role of nuclear-encoded mitochondrial tRNA charging enzymes in human inherited disease. Genes (Basel) 13:2319. doi: 10.3390/genes13122319 36553587 PMC9777667

[ref32] Deverman B. E. Ravina B. M. Bankiewicz K. S. Paul S. M. Sah D. W. Y. (2018). Gene therapy for neurological disorders: progress and prospects. Nat. Rev. Drug Discov. 17:767. doi: 10.1038/nrd.2018.158 30206384

[ref33] Devine M. J. Kittler J. T. (2018). Mitochondria at the neuronal presynapse in health and disease. Nat. Rev. Neurosci. 19, 63–80. doi: 10.1038/nrn.2017.170 29348666

[ref34] Dickinson M. E. Flenniken A. M. Ji X. Teboul L. Wong M. D. White J. K.. (2016). High-throughput discovery of novel developmental phenotypes. Nature 537, 508–514. doi: 10.1038/nature19356 27626380 PMC5295821

[ref35] Dogan S. A. Pujol C. Maiti P. Kukat A. Wang S. Hermans S.. (2014). Tissue-specific loss of DARS2 activates stress responses independently of respiratory chain deficiency in the heart. Cell Metab. 19, 458–469. doi: 10.1016/j.cmet.2014.02.004 24606902

[ref36] Echevarría L. Clemente P. Hernández-Sierra R. Gallardo M. E. Fernández-Moreno M. A. Garesse R. (2014). Glutamyl-tRNAGln amidotransferase is essential for mammalian mitochondrial translation in vivo. Biochem. J. 460, 91–101. doi: 10.1042/bj20131107 24579914

[ref37] Fan W. Jin X. Xu M. Xi Y. Lu W. Yang X.. (2021). FARS2 deficiency in Drosophila reveals the developmental delay and seizure manifested by aberrant mitochondrial tRNA metabolism. Nucleic Acids Res. 49, 13108–13121. doi: 10.1093/nar/gkab1187 34878141 PMC8682739

[ref38] Forrester S. J. Kikuchi D. S. Hernandes M. S. Xu Q. Griendling K. K. (2018). Reactive oxygen species in metabolic and inflammatory signaling. Circ. Res. 122, 877–902. doi: 10.1161/circresaha.117.311401 29700084 PMC5926825

[ref39] Fröhlich D. Kalotay E. von Jonquieres G. Bongers A. Lee B. Suchowerska A. K.. (2022). Dual-function AAV gene therapy reverses late-stage Canavan disease pathology in mice. Front. Mol. Neurosci. 15:1061257. doi: 10.3389/fnmol.2022.1061257 36568275 PMC9772617

[ref40] Fröhlich D. Mendes M. I. Kueh A. J. Bongers A. Herold M. J. Salomons G. S.. (2020). A Hypomorphic Dars1 (D367Y) model recapitulates key aspects of the Leukodystrophy HBSL. Front. Cell. Neurosci. 14:625879. doi: 10.3389/fncel.2020.625879 33551752 PMC7855723

[ref41] Fröhlich D. Suchowerska A. K. Spencer Z. H. von Jonquieres G. Klugmann C. B. Bongers A.. (2017). *In vivo* characterization of the aspartyl-tRNA synthetase DARS: homing in on the leukodystrophy HBSL. Neurobiol. Dis. 97, 24–35. doi: 10.1016/j.nbd.2016.10.008 27816769

[ref42] Fröhlich D. Suchowerska A. K. Voss C. He R. Wolvetang E. von Jonquieres G.. (2018). Expression pattern of the aspartyl-tRNA Synthetase DARS in the human brain. Front. Mol. Neurosci. 11:81. doi: 10.3389/fnmol.2018.00081 29615866 PMC5869200

[ref43] Fuchs S. A. Schene I. F. Kok G. Jansen J. M. Nikkels P. G. J. van Gassen K. L. I.. (2019). Aminoacyl-tRNA synthetase deficiencies in search of common themes. Genet. Med. 21, 319–330. doi: 10.1038/s41436-018-0048-y 29875423 PMC7091658

[ref44] Fukui H. Hanaoka R. Kawahara A. (2009). Noncanonical activity of seryl-tRNA synthetase is involved in vascular development. Circ. Res. 104, 1253–1259. doi: 10.1161/circresaha.108.191189 19423848 PMC2821192

[ref45] Giege R. Sissler M. Florentz C. (1998). Universal rules and idiosyncratic features in tRNA identity. Nucleic Acids Res. 26, 5017–5035. doi: 10.1093/nar/26.22.5017 9801296 PMC147952

[ref46] González-Serrano L. E. Chihade J. W. Sissler M. (2019). When a common biological role does not imply common disease outcomes: disparate pathology linked to human mitochondrial aminoacyl-tRNA synthetases. J. Biol. Chem. 294, 5309–5320. doi: 10.1074/jbc.REV118.002953 30647134 PMC6462531

[ref47] Guitart T. Picchioni D. Piñeyro D. Ribas de Pouplana L. (2013). Human mitochondrial disease-like symptoms caused by a reduced tRNA aminoacylation activity in flies. Nucleic Acids Res. 41, 6595–6608. doi: 10.1093/nar/gkt402 23677612 PMC3711456

[ref48] Guo M. Schimmel P. (2013). Essential nontranslational functions of tRNA synthetases. Nat. Chem. Biol. 9, 145–153. doi: 10.1038/nchembio.1158 23416400 PMC3773598

[ref49] Hadchouel A. Wieland T. Griese M. Baruffini E. Lorenz-Depiereux B. Enaud L.. (2015). Biallelic mutations of Methionyl-tRNA Synthetase cause a specific type of pulmonary alveolar Proteinosis prevalent on Réunion Island. Am. J. Hum. Genet. 96, 826–831. doi: 10.1016/j.ajhg.2015.03.010 25913036 PMC4570277

[ref50] Han J. M. Jeong S. J. Park M. C. Kim G. Kwon N. H. Kim H. K.. (2012). Leucyl-tRNA synthetase is an intracellular leucine sensor for the mTORC1-signaling pathway. Cells 149, 410–424. doi: 10.1016/j.cell.2012.02.044 22424946

[ref51] Herzog W. Müller K. Huisken J. Stainier D. Y. (2009). Genetic evidence for a noncanonical function of seryl-tRNA synthetase in vascular development. Circ. Res. 104, 1260–1266. doi: 10.1161/circresaha.108.191718 19423847 PMC2726715

[ref52] Hilander T. Zhou X. L. Konovalova S. Zhang F. P. Euro L. Chilov D.. (2018). Editing activity for eliminating mischarged tRNAs is essential in mammalian mitochondria. Nucleic Acids Res. 46, 849–860. doi: 10.1093/nar/gkx1231 29228266 PMC5778596

[ref53] Hines T. J. Tadenev A. L. D. Lone M. A. Hatton C. L. Bagasrawala I. Stum M. G.. (2021). Precision mouse models of Yars/dominant intermediate Charcot-Marie-tooth disease type C and Sptlc1/hereditary sensory and autonomic neuropathy type 1. J. Anat. 241, 1169–1185. doi: 10.1111/joa.13605 34875719 PMC9170831

[ref54] Ho M. T. Lu J. Brunßen D. Suter B. (2021). A translation-independent function of Phe RS activates growth and proliferation in Drosophila. Dis. Model. Mech. 14:dmm048132. doi: 10.1242/dmm.048132 33547043 PMC7988764

[ref55] Hoppler S. Conlon F. L. (2020). Xenopus: experimental access to cardiovascular development, regeneration discovery, and cardiovascular heart-defect modeling. Cold Spring Harb. Perspect. Biol. 12:a037200. doi: 10.1101/cshperspect.a037200 31767648 PMC7263084

[ref56] Hudry E. Vandenberghe L. H. (2019). Therapeutic AAV gene transfer to the nervous system: a clinical reality. Neuron 101, 839–862. doi: 10.1016/j.neuron.2019.02.017 30844402 PMC11804970

[ref57] Inoue M. Miyahara H. Shiraishi H. Shimizu N. Tsumori M. Kiyota K.. (2021). Leucyl-tRNA synthetase deficiency systemically induces excessive autophagy in zebrafish. Sci. Rep. 11:8392. doi: 10.1038/s41598-021-87879-4 33863987 PMC8052342

[ref58] Itoh M. Dai H. Horike S. I. Gonzalez J. Kitami Y. Meguro-Horike M.. (2019). Biallelic KARS pathogenic variants cause an early-onset progressive leukodystrophy. Brain 142, 560–573. doi: 10.1093/brain/awz001 30715177

[ref59] Jeong J. H. Ojha U. Lee Y. M. (2021). Pathological angiogenesis and inflammation in tissues. Arch. Pharm. Res. 44, 1–15. doi: 10.1007/s12272-020-01287-2 PMC7682773 33230600

[ref60] Jiang P. Jin X. Peng Y. Wang M. Liu H. Liu X.. (2016). The exome sequencing identified the mutation in YARS2 encoding the mitochondrial tyrosyl-tRNA synthetase as a nuclear modifier for the phenotypic manifestation of Leber's hereditary optic neuropathy-associated mitochondrial DNA mutation. Hum. Mol. Genet. 25, 584–596. doi: 10.1093/hmg/ddv498 26647310

[ref61] Jin S. W. Herzog W. Santoro M. M. Mitchell T. S. Frantsve J. Jungblut B.. (2007). A transgene-assisted genetic screen identifies essential regulators of vascular development in vertebrate embryos. Dev. Biol. 307, 29–42. doi: 10.1016/j.ydbio.2007.03.526 17531218 PMC2695512

[ref62] Jin D. Wek S. A. Cordova R. A. Wek R. C. Lacombe D. Michaud V.. (2022). Aminoacylation-defective bi-allelic mutations in human EPRS1 associated with psychomotor developmental delay, epilepsy, and deafness. Clin. Genet. 103, 358–363. doi: 10.1111/cge.14269 36411955 PMC9898101

[ref63] Jin X. Zhang Z. Nie Z. Wang C. Meng F. Yi Q.. (2021). An animal model for mitochondrial tyrosyl-tRNA synthetase deficiency reveals links between oxidative phosphorylation and retinal function. J. Biol. Chem. 296:100437. doi: 10.1016/j.jbc.2021.100437 33610547 PMC8010715

[ref64] Kaiser F. Krautwurst S. Salentin S. Haupt V. J. Leberecht C. Bittrich S.. (2020). The structural basis of the genetic code: amino acid recognition by aminoacyl-tRNA synthetases. Sci. Rep. 10:12647. doi: 10.1038/s41598-020-69100-0 32724042 PMC7387524

[ref65] Kapur M. Ackerman S. L. (2018). mRNA translation gone awry: translation Fidelity and neurological disease. Trends Genet. 34, 218–231. doi: 10.1016/j.tig.2017.12.007 29352613 PMC5834357

[ref66] Kasher P. R. Namavar Y. van Tijn P. Fluiter K. Sizarov A. Kamermans M.. (2011). Impairment of the tRNA-splicing endonuclease subunit 54 (tsen54) gene causes neurological abnormalities and larval death in zebrafish models of pontocerebellar hypoplasia. Hum. Mol. Genet. 20, 1574–1584. doi: 10.1093/hmg/ddr034 21273289

[ref67] Kausar S. Wang F. Cui H. (2018). The role of mitochondria in reactive oxygen species generation and its implications for neurodegenerative diseases. Cells 7:274. doi: 10.3390/cells7120274 30563029 PMC6316843

[ref68] Kayvanpour E. Wisdom M. Lackner M. K. Sedaghat-Hamedani F. Boeckel J. N. Müller M.. (2022). VARS2 depletion leads to activation of the integrated stress response and disruptions in mitochondrial fatty acid oxidation. Int. J. Mol. Sci. 23:7327. doi: 10.3390/ijms23137327 35806332 PMC9267100

[ref69] Kells A. P. Goulet M. Aubin J. Yuan S. Dismuke D. Reed R. P.. (2015). 502. Optimization of intrathecal delivery of AAV for targeting the spinal compartment. Mol. Ther. 23, S200–S201. doi: 10.1016/S1525-0016(16)34111-9

[ref70] Kim M. J. Park B. J. Kang Y. S. Kim H. J. Park J. H. Kang J. W.. (2003). Downregulation of FUSE-binding protein and c-myc by tRNA synthetase cofactor p 38 is required for lung cell differentiation. Nat. Genet. 34, 330–336. doi: 10.1038/ng1182 12819782

[ref71] Klugmann M. Kalotay E. Delerue F. Ittner L. M. Bongers A. Yu J.. (2022). Developmental delay and late onset HBSL pathology in hypomorphic Dars1(M256L) mice. Neurochem. Res. 47, 1972–1984. doi: 10.1007/s11064-022-03582-4 35357600 PMC9217827

[ref72] Kok G. Tseng L. Schene I. F. Dijsselhof M. E. Salomons G. Mendes M. I.. (2021). Treatment of ARS deficiencies with specific amino acids. Genet. Med. 23, 2202–2207. doi: 10.1038/s41436-021-01249-z 34194004 PMC8244667

[ref73] Kopajtich R. Murayama K. Janecke A. R. Haack T. B. Breuer M. Knisely A. S.. (2016). Biallelic IARS mutations cause growth retardation with prenatal onset, intellectual disability, muscular Hypotonia, and infantile Hepatopathy. Am. J. Hum. Genet. 99, 414–422. doi: 10.1016/j.ajhg.2016.05.027 27426735 PMC4974065

[ref74] Köster R. Sassen W. (2015). A molecular toolbox for genetic manipulation of zebrafish. Adv Genom Genet 5:151. doi: 10.2147/AGG.S57585

[ref75] Kulkarni V. A. Firestein B. L. (2012). The dendritic tree and brain disorders. Mol. Cell. Neurosci. 50, 10–20. doi: 10.1016/j.mcn.2012.03.005 22465229

[ref76] Kuo M. E. Antonellis A. (2020). Ubiquitously expressed proteins and restricted phenotypes: exploring cell-specific sensitivities to impaired tRNA charging. Trends Genet. 36, 105–117. doi: 10.1016/j.tig.2019.11.007 31839378 PMC6980692

[ref77] Lasser M. Pratt B. Monahan C. Kim S. W. Lowery L. A. (2019). The many faces of Xenopus: *Xenopus laevis* as a model system to study Wolf-Hirschhorn syndrome. Front. Physiol. 10:817. doi: 10.3389/fphys.2019.00817 31297068 PMC6607408

[ref78] Lee J. W. Beebe K. Nangle L. A. Jang J. Longo-Guess C. M. Cook S. A.. (2006). Editing-defective tRNA synthetase causes protein misfolding and neurodegeneration. Nature 443, 50–55. doi: 10.1038/nature05096 16906134

[ref79] Lee E. Y. Lee H. C. Kim H. K. Jang S. Y. Park S. J. Kim Y. H.. (2016). Infection-specific phosphorylation of glutamyl-prolyl tRNA synthetase induces antiviral immunity. Nat. Immunol. 17, 1252–1262. doi: 10.1038/ni.3542 27595231 PMC5173487

[ref80] Lee-Liu D. Méndez-Olivos E. E. Muñoz R. Larraín J. (2017). The African clawed frog *Xenopus laevis*: a model organism to study regeneration of the central nervous system. Neurosci. Lett. 652, 82–93. doi: 10.1016/j.neulet.2016.09.054 27693567

[ref81] Lenz D. Smith D. E. C. Crushell E. Husain R. A. Salomons G. S. Alhaddad B.. (2020a). Genotypic diversity and phenotypic spectrum of infantile liver failure syndrome type 1 due to variants in LARS1. Genet. Med. 22, 1863–1873. doi: 10.1038/s41436-020-0904-4 32699352

[ref82] Lenz D. Stahl M. Seidl E. Schöndorf D. Brennenstuhl H. Gesenhues F.. (2020b). Rescue of respiratory failure in pulmonary alveolar proteinosis due to pathogenic MARS1 variants. Pediatr. Pulmonol. 55, 3057–3066. doi: 10.1002/ppul.25031 32833345

[ref83] Lin S. J. Vona B. Barbalho P. G. Kaiyrzhanov R. Maroofian R. Petree C.. (2021). Biallelic variants in KARS1 are associated with neurodevelopmental disorders and hearing loss recapitulated by the knockout zebrafish. Genet. Med. 23, 1933–1943. doi: 10.1038/s41436-021-01239-1 34172899 PMC8956360

[ref84] Lin S. J. Vona B. Porter H. M. Izadi M. Huang K. Lacassie Y.. (2022). Biallelic variants in WARS1 cause a highly variable neurodevelopmental syndrome and implicate a critical exon for normal auditory function. Hum. Mutat. 43, 1472–1489. doi: 10.1002/humu.24435 35815345

[ref85] Lin J. H. Walter P. Yen T. S. (2008). Endoplasmic reticulum stress in disease pathogenesis. Annu. Rev. Pathol. 3, 399–425. doi: 10.1146/annurev.pathmechdis.3.121806.151434 18039139 PMC3653419

[ref86] Liu Y. Satz J. S. Vo M. N. Nangle L. A. Schimmel P. Ackerman S. L. (2014). Deficiencies in tRNA synthetase editing activity cause cardioproteinopathy. Proc. Natl. Acad. Sci. U. S. A. 111, 17570–17575. doi: 10.1073/pnas.1420196111 25422440 PMC4267364

[ref87] Lo W. S. Gardiner E. Xu Z. Lau C. F. Wang F. Zhou J. J.. (2014). Human tRNA synthetase catalytic nulls with diverse functions. Science 345, 328–332. doi: 10.1126/science.1252943 25035493 PMC4188629

[ref88] Lu J. Bergert M. Walther A. Suter B. (2014). Double-sieving-defective aminoacyl-tRNA synthetase causes protein mistranslation and affects cellular physiology and development. Nat. Commun. 5:5650. doi: 10.1038/ncomms6650 25427601 PMC4263187

[ref89] Lu B. Vogel H. (2009). Drosophila models of neurodegenerative diseases. Annu. Rev. Pathol. 4, 315–342. doi: 10.1146/annurev.pathol.3.121806.151529 18842101 PMC3045805

[ref90] Maffezzini C. Laine I. Dallabona C. Clemente P. Calvo-Garrido J. Wibom R.. (2019). Mutations in the mitochondrial tryptophanyl-tRNA synthetase cause growth retardation and progressive leukoencephalopathy. Mol Genet Genomic Med 7:e654. doi: 10.1002/mgg3.654 30920170 PMC6565557

[ref91] Manole A. Efthymiou S. O'Connor E. Mendes M. I. Jennings M. Maroofian R.. (2020). De novo and bi-allelic pathogenic variants in NARS1 cause neurodevelopmental delay due to toxic gain-of-function and partial loss-of-function effects. Am. J. Hum. Genet. 107, 311–324. doi: 10.1016/j.ajhg.2020.06.016 32738225 PMC7413890

[ref92] Marchi P. M. Marrone L. Azzouz M. (2022). Delivery of therapeutic AAV9 vectors via cisterna magna to treat neurological disorders. Trends Mol. Med. 28, 79–80. doi: 10.1016/j.molmed.2021.09.007 34756547

[ref93] Meyer-Schuman R. Antonellis A. (2017). Emerging mechanisms of aminoacyl-tRNA synthetase mutations in recessive and dominant human disease. Hum. Mol. Genet. 26, R114–R127. doi: 10.1093/hmg/ddx231 28633377 PMC5886470

[ref94] Mirando A. C. Francklyn C. S. Lounsbury K. M. (2014). Regulation of angiogenesis by aminoacyl-tRNA synthetases. Int. J. Mol. Sci. 15, 23725–23748. doi: 10.3390/ijms151223725 25535072 PMC4284789

[ref95] Mo L. Li R. He C. Chen Q. Xu C. Shen L.. (2023). Hedgehog pathway is negatively regulated during the development of *Drosophila melanogaster* Phe RS-m (Drosophila homologs gene of human FARS2) mutants. Hum. Cell 36, 121–131. doi: 10.1007/s13577-022-00796-0 36205831

[ref96] Murofushi Y. Hayakawa I. Abe Y. Ohto T. Murayama K. Suzuki H.. (2022). Ketogenic diet for KARS-related mitochondrial dysfunction and progressive Leukodystrophy. Neuropediatrics 53, 65–68. doi: 10.1055/s-0041-1732446 34448181

[ref97] Mušo M. Bentley L. Vizor L. Yon M. Burling K. Barker P.. (2022). A Wars2 mutant mouse shows a sex and diet specific change in fat distribution, reduced food intake and depot-specific upregulation of WAT browning. Front. Physiol. 13:953199. doi: 10.3389/fphys.2022.953199 36091365 PMC9452902

[ref98] Muthiah A. Housley G. D. Klugmann M. Fröhlich D. (2020). The Leukodystrophies HBSL and LBSL-correlates and distinctions. Front. Cell. Neurosci. 14:626610. doi: 10.3389/fncel.2020.626610 33574740 PMC7870476

[ref99] Naso M. F. Tomkowicz B. Perry W. L. 3rd Strohl W. R. (2017). Adeno-associated virus (AAV) as a vector for gene therapy. Bio Drugs 31, 317–334. doi: 10.1007/s40259-017-0234-5 PMC5548848 28669112

[ref100] Nayak P. Kejriwal A. Ratnaparkhi G. S. (2021). SUMOylation of Arginyl tRNA Synthetase modulates the Drosophila innate immune response. Front. Cell Dev. Biol. 9:695630. doi: 10.3389/fcell.2021.695630 34660574 PMC8514731

[ref101] Nemeth C. L. Tomlinson S. N. Rosen M. O'Brien B. M. Larraza O. Jain M.. (2020). Neuronal ablation of mt-AspRS in mice induces immune pathway activation prior to severe and progressive cortical and behavioral disruption. Exp. Neurol. 326:113164. doi: 10.1016/j.expneurol.2019.113164 31887305 PMC7448750

[ref102] Ofir-Birin Y. Fang P. Bennett S. P. Zhang H. M. Wang J. Rachmin I.. (2013). Structural switch of lysyl-tRNA synthetase between translation and transcription. Mol. Cell 49, 30–42. doi: 10.1016/j.molcel.2012.10.010 23159739 PMC3766370

[ref103] Ognjenovic J. Simonovic M. (2018). Human aminoacyl-tRNA synthetases in diseases of the nervous system. RNA Biol. 15, 623–634. doi: 10.1080/15476286.2017.1330245 28534666 PMC6103678

[ref104] Ong M. T. Willoughby J. Connolly D. J. Mordekar S. Johnson D. (2020). Genotype–phenotype variability of DARS mutation-case reports of a trio of siblings. Eur J Med Case Rep 4, 110–115. doi: 10.24911/ejmcr/173-1551044010

[ref105] Oprescu S. N. Griffin L. B. Beg A. A. Antonellis A. (2017). Predicting the pathogenicity of aminoacyl-tRNA synthetase mutations. Methods 113, 139–151. doi: 10.1016/j.ymeth.2016.11.013 27876679 PMC5253330

[ref106] Pang Y. L. Poruri K. Martinis S. A. (2014). tRNA synthetase: tRNA aminoacylation and beyond. Wiley Interdisc Rev RNA 5, 461–480. doi: 10.1002/wrna.1224 PMC4062602 24706556

[ref107] Park S. G. Kim H. J. Min Y. H. Choi E. C. Shin Y. K. Park B. J.. (2005). Human lysyl-tRNA synthetase is secreted to trigger proinflammatory response. Proc. Natl. Acad. Sci. U. S. A. 102, 6356–6361. doi: 10.1073/pnas.0500226102 15851690 PMC1088368

[ref108] Peguera B. Segarra M. Acker-Palmer A. (2021). Neurovascular crosstalk coordinates the central nervous system development. Curr. Opin. Neurobiol. 69, 202–213. doi: 10.1016/j.conb.2021.04.005 34077852 PMC8411665

[ref109] Perona J. J. Gruic-Sovulj I. (2014). Synthetic and editing mechanisms of aminoacyl-tRNA synthetases. Top. Curr. Chem. 344, 1–41. doi: 10.1007/128_2013_456 23852030

[ref110] Pierce S. B. Chisholm K. M. Lynch E. D. Lee M. K. Walsh T. Opitz J. M.. (2011). Mutations in mitochondrial histidyl tRNA synthetase HARS2 cause ovarian dysgenesis and sensorineural hearing loss of Perrault syndrome. Proc. Natl. Acad. Sci. U. S. A. 108, 6543–6548. doi: 10.1073/pnas.1103471108 21464306 PMC3081023

[ref111] Pierce S. B. Gersak K. Michaelson-Cohen R. Walsh T. Lee M. K. Malach D.. (2013). Mutations in LARS2, encoding mitochondrial leucyl-tRNA synthetase, lead to premature ovarian failure and hearing loss in Perrault syndrome. Am. J. Hum. Genet. 92, 614–620. doi: 10.1016/j.ajhg.2013.03.007 23541342 PMC3617377

[ref112] Potter P. K. Bowl M. R. Jeyarajan P. Wisby L. Blease A. Goldsworthy M. E.. (2016). Novel gene function revealed by mouse mutagenesis screens for models of age-related disease. Nat. Commun. 7:12444. doi: 10.1038/ncomms12444 27534441 PMC4992138

[ref113] Preston M. A. Macklin W. B. (2015). Zebrafish as a model to investigate CNS myelination. Glia 63, 177–193. doi: 10.1002/glia.22755 25263121 PMC4539269

[ref114] Puffenberger E. G. Jinks R. N. Sougnez C. Cibulskis K. Willert R. A. Achilly N. P.. (2012). Genetic mapping and exome sequencing identify variants associated with five novel diseases. PLoS One 7:e28936. doi: 10.1371/journal.pone.0028936 22279524 PMC3260153

[ref115] Ribas de Pouplana L. Schimmel P. (2001). Two classes of tRNA synthetases suggested by sterically compatible dockings on tRNA acceptor stem. Cells 104, 191–193. doi: 10.1016/s0092-8674(01)00204-5 11269237

[ref116] Riley L. G. Menezes M. J. Rudinger-Thirion J. Duff R. de Lonlay P. Rotig A.. (2013). Phenotypic variability and identification of novel YARS2 mutations in YARS2 mitochondrial myopathy, lactic acidosis and sideroblastic anaemia. Orphanet J. Rare Dis. 8:193. doi: 10.1186/1750-1172-8-193 24344687 PMC3878580

[ref117] Rubio Gomez M. A. Ibba M. (2020). Aminoacyl-tRNA synthetases. RNA 26, 910–936. doi: 10.1261/rna.071720.119 32303649 PMC7373986

[ref118] Rumyantseva A. Motori E. Trifunovic A. (2020). DARS2 is indispensable for Purkinje cell survival and protects against cerebellar ataxia. Hum. Mol. Genet. 29, 2845–2854. doi: 10.1093/hmg/ddaa176 32766765

[ref119] Santos-Cortez R. L. Lee K. Azeem Z. Antonellis P. J. Pollock L. M. Khan S.. (2013). Mutations in KARS, encoding lysyl-tRNA synthetase, cause autosomal-recessive nonsyndromic hearing impairment DFNB89. Am. J. Hum. Genet. 93, 132–140. doi: 10.1016/j.ajhg.2013.05.018 23768514 PMC3710764

[ref120] Scheper G. C. van der Klok T. van Andel R. J. van Berkel C. G. Sissler M. Smet J.. (2007). Mitochondrial aspartyl-tRNA synthetase deficiency causes leukoencephalopathy with brain stem and spinal cord involvement and lactate elevation. Nat. Genet. 39, 534–539. doi: 10.1038/ng2013 17384640

[ref121] Schuch L. A. Forstner M. Rapp C. K. Li Y. Smith D. E. C. Mendes M. I.. (2021). FARS1-related disorders caused by bi-allelic mutations in cytosolic phenylalanyl-tRNA synthetase genes: look beyond the lungs! Clin. Genet. 99, 789–801. doi: 10.1111/cge.13943 33598926

[ref122] Seburn K. L. Nangle L. A. Cox G. A. Schimmel P. Burgess R. W. (2006). An active dominant mutation of glycyl-tRNA synthetase causes neuropathy in a Charcot-Marie-tooth 2D mouse model. Neuron 51, 715–726. doi: 10.1016/j.neuron.2006.08.027 16982418

[ref123] Shi Y. Liu Z. Zhang Q. Vallee I. Mo Z. Kishi S.. (2020). Phosphorylation of seryl-tRNA synthetase by ATM/ATR is essential for hypoxia-induced angiogenesis. PLoS Biol. 18:e3000991. doi: 10.1371/journal.pbio.3000991 33351793 PMC7755189

[ref124] Shi Y. Xu X. Zhang Q. Fu G. Mo Z. Wang G. S.. (2014). tRNA synthetase counteracts c-Myc to develop functional vasculature. elife 3:e02349. doi: 10.7554/eLife.02349 24940000 PMC4057782

[ref125] Shpilka T. Haynes C. M. (2018). The mitochondrial UPR: mechanisms, physiological functions and implications in ageing. Nat. Rev. Mol. Cell Biol. 19, 109–120. doi: 10.1038/nrm.2017.110 29165426

[ref126] Siekierska A. Stamberger H. Deconinck T. Oprescu S. N. Partoens M. Zhang Y.. (2019). Biallelic VARS variants cause developmental encephalopathy with microcephaly that is recapitulated in vars knockout zebrafish. Nat. Commun. 10:708. doi: 10.1038/s41467-018-07953-w 30755616 PMC6372652

[ref127] Simon M. Richard E. M. Wang X. Shahzad M. Huang V. H. Qaiser T. A.. (2015). Mutations of human NARS2, encoding the mitochondrial asparaginyl-tRNA synthetase, cause nonsyndromic deafness and Leigh syndrome. PLoS Genet. 11:e1005097. doi: 10.1371/journal.pgen.1005097 25807530 PMC4373692

[ref128] Sissler M. González-Serrano L. E. Westhof E. (2017). Recent advances in mitochondrial aminoacyl-tRNA Synthetases and disease. Trends Mol. Med. 23, 693–708. doi: 10.1016/j.molmed.2017.06.002 28716624

[ref129] Soldà G. Caccia S. Robusto M. Chiereghin C. Castorina P. Ambrosetti U.. (2016). First independent replication of the involvement of LARS2 in Perrault syndrome by whole-exome sequencing of an Italian family. J. Hum. Genet. 61, 295–300. doi: 10.1038/jhg.2015.149 26657938 PMC4817218

[ref130] Song Y. Shi Y. Carland T. M. Lian S. Sasaki T. Schork N. J.. (2016). p53-dependent DNA damage response sensitive to editing-defective tRNA synthetase in zebrafish. Proc. Natl. Acad. Sci. U. S. A. 113, 8460–8465. doi: 10.1073/pnas.1608139113 27402763 PMC4968768

[ref131] Stewart A. M. Braubach O. Spitsbergen J. Gerlai R. Kalueff A. V. (2014). Zebrafish models for translational neuroscience research: from tank to bedside. Trends Neurosci. 37, 264–278. doi: 10.1016/j.tins.2014.02.011 24726051 PMC4039217

[ref132] Sukoff Rizzo S. J. Crawley J. N. (2017). Behavioral phenotyping assays for genetic mouse models of neurodevelopmental, neurodegenerative, and psychiatric disorders. Annu Rev Anim Biosci 5, 371–389. doi: 10.1146/annurev-animal-022516-022754 28199172

[ref133] Sun C. Song J. Jiang Y. Zhao C. Lu J. Li Y.. (2019). Loss-of-function mutations in Lysyl-tRNA synthetase cause various leukoencephalopathy phenotypes. Neurol Genet 5:e565. doi: 10.1212/nxg.0000000000000316 31192300 PMC6515944

[ref134] Sundal C. Carmona S. Yhr M. Almstrom O. Ljungberg M. Hardy J.. (2019). An AARS variant as the likely cause of Swedish type hereditary diffuse leukoencephalopathy with spheroids. Acta Neuropathol. Commun. 7:188. doi: 10.1186/s40478-019-0843-y 31775912 PMC6880494

[ref135] Tadenev A. L. D. Burgess R. W. (2019). Model validity for preclinical studies in precision medicine: precisely how precise do we need to be? Mamm. Genome 30, 111–122. doi: 10.1007/s00335-019-09798-0 30953144 PMC6606658

[ref136] Taft R. J. Vanderver A. Leventer R. J. Damiani S. A. Simons C. Grimmond S. M.. (2013). Mutations in DARS cause hypomyelination with brain stem and spinal cord involvement and leg spasticity. Am. J. Hum. Genet. 92, 774–780. doi: 10.1016/j.ajhg.2013.04.006 23643384 PMC3644624

[ref137] Theisen B. E. Rumyantseva A. Cohen J. S. Alcaraz W. A. Shinde D. N. Tang S.. (2017). Deficiency of WARS2, encoding mitochondrial tryptophanyl tRNA synthetase, causes severe infantile onset leukoencephalopathy. Am. J. Med. Genet. A 173, 2505–2510. doi: 10.1002/ajmg.a.38339 28650581

[ref138] Van Battum E. Y. Brignani S. Pasterkamp R. J. (2015). Axon guidance proteins in neurological disorders. Lancet Neurol. 14, 532–546. doi: 10.1016/s1474-4422(14)70257-1 25769423

[ref139] van Berge L. Kevenaar J. Polder E. Gaudry A. Florentz C. Sissler M.. (2013). Pathogenic mutations causing LBSL affect mitochondrial aspartyl-tRNA synthetase in diverse ways. Biochem. J. 450, 345–350. doi: 10.1042/BJ20121564 23216004

[ref140] Vantroys E. Larson A. Friederich M. Knight K. Swanson M. A. Powell C. A.. (2017). New insights into the phenotype of FARS2 deficiency. Mol. Genet. Metab. 122, 172–181. doi: 10.1016/j.ymgme.2017.10.004 29126765 PMC5734183

[ref141] Veldman M. B. Lin S. (2008). Zebrafish as a developmental model organism for pediatric research. Pediatr. Res. 64, 470–476. doi: 10.1203/PDR.0b013e318186e609 18679162

[ref142] Virdee M. Swarnalingam E. Kozenko M. Tarnopolsky M. Jones K. (2019). Expanding the phenotype: neurodevelopmental disorder, mitochondrial, with abnormal movements and lactic acidosis, with or without seizures (NEMMLAS) due to WARS2 Biallelic variants, encoding mitochondrial Tryptophanyl-tRNA synthase. J. Child Neurol. 34, 778–781. doi: 10.1177/0883073819854604 31282308

[ref143] Vo M. N. Terrey M. Lee J. W. Roy B. Moresco J. J. Sun L.. (2018). ANKRD16 prevents neuron loss caused by an editing-defective tRNA synthetase. Nature 557, 510–515. doi: 10.1038/s41586-018-0137-8 29769718 PMC5973781

[ref144] Vogenstahl J. Parrilla M. Acker-Palmer A. Segarra M. (2022). Vascular regulation of developmental neurogenesis. Front. Cell Dev. Biol. 10:890852. doi: 10.3389/fcell.2022.890852 35573692 PMC9099230

[ref145] von Jonquieres G. Frohlich D. Klugmann C. B. Wen X. Harasta A. E. Ramkumar R.. (2016). Recombinant human myelin-associated glycoprotein promoter drives selective AAV-mediated transgene expression in oligodendrocytes. Front. Mol. Neurosci. 9:13. doi: 10.3389/fnmol.2016.00013 26941604 PMC4763065

[ref146] von Jonquieres G. Mersmann N. Klugmann C. B. Harasta A. E. Lutz B. Teahan O.. (2013). Glial promoter selectivity following AAV-delivery to the immature brain. PLoS One 8:e65646. doi: 10.1371/journal.pone.0065646 23799030 PMC3683058

[ref147] Waldron A. Wilcox C. Francklyn C. Ebert A. (2019). Knock-down of Histidyl-tRNA Synthetase causes cell cycle arrest and apoptosis of neuronal progenitor cells in vivo. Front. Cell Dev. Biol. 7:67. doi: 10.3389/fcell.2019.00067 31134197 PMC6524715

[ref148] Walter P. Ron D. (2011). The unfolded protein response: from stress pathway to homeostatic regulation. Science 334, 1081–1086. doi: 10.1126/science.1209038 22116877

[ref149] Wang F. Huang G. D. Tian H. Zhong Y. B. Shi H. J. Li Z.. (2015). Point mutations in KAL1 and the mitochondrial gene MT-tRNA (cys) synergize to produce Kallmann syndrome phenotype. Sci. Rep. 5:13050. doi: 10.1038/srep13050 26278626 PMC4642522

[ref150] Wang M. Sips P. Khin E. Rotival M. Sun X. Ahmed R.. (2016). Wars2 is a determinant of angiogenesis. Nat. Commun. 7:12061. doi: 10.1038/ncomms12061 27389904 PMC4941120

[ref151] Wang Z. Song J. Luo L. Ma J. (2018). Loss of Leucyl-tRNA synthetase b leads to ILFS1-like symptoms in zebrafish. Biochem. Biophys. Res. Commun. 505, 378–384. doi: 10.1016/j.bbrc.2018.09.133 30262142

[ref152] Wang K. Zhao S. Liu B. Zhang Q. Li Y. Liu J.. (2018). Perturbations of BMP/TGF-β and VEGF/VEGFR signalling pathways in non-syndromic sporadic brain arteriovenous malformations (BAVM). J. Med. Genet. 55, 675–684. doi: 10.1136/jmedgenet-2017-105224 30120215 PMC6161649

[ref153] Wei N. Zhang Q. Yang X. L. (2019). Neurodegenerative Charcot-Marie-tooth disease as a case study to decipher novel functions of aminoacyl-tRNA synthetases. J. Biol. Chem. 294, 5321–5339. doi: 10.1074/jbc.REV118.002955 30643024 PMC6462521

[ref154] Wolf N. I. Toro C. Kister I. Latif K. A. Leventer R. Pizzino A.. (2015). DARS-associated leukoencephalopathy can mimic a steroid-responsive neuroinflammatory disorder. Neurology 84, 226–230. doi: 10.1212/WNL.0000000000001157 25527264 PMC4335995

[ref155] Wortmann S. B. Timal S. Venselaar H. Wintjes L. T. Kopajtich R. Feichtinger R. G.. (2017). Biallelic variants in WARS2 encoding mitochondrial tryptophanyl-tRNA synthase in six individuals with mitochondrial encephalopathy. Hum. Mutat. 38, 1786–1795. doi: 10.1002/humu.23340 28905505

[ref156] Xu X. Shi Y. Zhang H. M. Swindell E. C. Marshall A. G. Guo M.. (2012). Unique domain appended to vertebrate tRNA synthetase is essential for vascular development. Nat. Commun. 3:681. doi: 10.1038/ncomms1686 22353712 PMC3293412

[ref157] Xu P. Wang L. Peng H. Liu H. Liu H. Yuan Q.. (2021). Disruption of Hars 2 in Cochlear hair cells causes progressive mitochondrial dysfunction and hearing loss in mice. Front. Cell. Neurosci. 15:804345. doi: 10.3389/fncel.2021.804345 34975414 PMC8715924

[ref158] Yang Y. Liu W. Fang Z. Shi J. Che F. He C.. (2016). A newly identified missense mutation in FARS2 causes autosomal-recessive spastic paraplegia. Hum. Mutat. 37, 165–169. doi: 10.1002/humu.22930 26553276

[ref159] Yoo S. Y. Kwon S. M. (2013). Angiogenesis and its therapeutic opportunities. Mediat. Inflamm. 2013:127170. doi: 10.1155/2013/127170 PMC3745966 23983401

[ref160] Yu T. Zhang Y. Zheng W. Q. Wu S. Li G. Zhang Y.. (2022). Selective degradation of tRNASer(AGY) is the primary driver for mitochondrial seryl-tRNA synthetase-related disease. Nucleic Acids Res. 50, 11755–11774. doi: 10.1093/nar/gkac1028, PMID: 36350636 PMC9723649

[ref161] Zeng R. Wang M. You G. Y. Yue R. Z. Chen Y. C. Zeng Z.. (2016). Effect of Mini-Tyrosyl-tRNA Synthetase/Mini-Tryptophanyl-tRNA Synthetase on angiogenesis in Rhesus monkeys after acute myocardial infarction. Cardiovasc. Ther. 34, 4–12. doi: 10.1111/1755-5922.12161 26400816

[ref162] Zhang X. Ling J. Barcia G. Jing L. Wu J. Barry B. J.. (2014). Mutations in QARS, encoding glutaminyl-tRNA synthetase, cause progressive microcephaly, cerebral-cerebellar atrophy, and intractable seizures. Am. J. Hum. Genet. 94, 547–558. doi: 10.1016/j.ajhg.2014.03.003 24656866 PMC3980424

[ref163] Zhang F. Zeng Q. Y. Xu H. Xu A. N. Liu D. J. Li N. Z.. (2021). Selective and competitive functions of the AAR and UPR pathways in stress-induced angiogenesis. Cell Discov 7:98. doi: 10.1038/s41421-021-00332-8 34697290 PMC8547220

[ref164] Zhou Z. Sun B. Yu D. Bian M. (2021). Roles of tRNA metabolism in aging and lifespan. Cell Death Dis. 12:548. doi: 10.1038/s41419-021-03838-x 34039958 PMC8154886

[ref165] Zou Y. Yang Y. Fu X. He X. Liu M. Zong T.. (2021). The regulatory roles of aminoacyl-tRNA synthetase in cardiovascular disease. Mol Ther Nucleic Acids 25, 372–387. doi: 10.1016/j.omtn.2021.06.003 34484863 PMC8399643

