# Supplementary material for: Recessive aminoacyl-tRNA synthetase disorders: lessons learned from in vivo disease models
Source: Front Neurosci. 2023 May 9;17:1182874. doi: 10.3389/fnins.2023.1182874 (PMC10234152; doi:10.3389/fnins.2023.1182874)
Supplement: Supplementary file 1 [file Table_1.pdf]

Supplementary Table 1. Diseases caused by recessive mutations in cytosolic ARSs and associated animal studies. Some mouse ARS knockout models were generated and phenotyped by the International Mouse Phenotyping Consortium (IMPC), with data available at www.mousephenotype.org. Note that in *C. elegans*, the genes corresponding to the cytosolic alanyl- and valyl-tRNA synthetases are *aars-2* and *vars-2*. Other *C. elegans* genes follow the standard pattern of nomenclature, where cytosolic and bifunctional ARS genes are assigned the suffix *-1* and mitochondrial genes are assigned the suffix *-2*.

Human ARS gene	Clinical presentation(s)	Animal studies and disease models
AARS1	• Developmental and epileptic encephalopathy 29 [OMIM #616339] (Simons et al., 2015; Nakayama et al., 2017; Helman et al., 2021)<br>• Late-onset posterior-predominant leukoencephalopathy (Helman et al., 2021)<br>• Microcephaly, developmental delay, recurrent acute liver failure (Marten et al., 2020)<br>• Nonphotosensitive trichothiodystrophy 8; TTD8 [OMIM #619691] (Botta et al., 2021)	***C. elegans***<br>• RNAi *aars-2* knockdown (Zheng et al., 2022)<br>**Mouse (*Mus musculus*)**<br>• Editing domain 'sticky' *(sti)* mutation in *Aars1* (Lee et al., 2006; Vo et al., 2018)<br>• Introduction of multiple (mild to severe) editing domain mutations into *Aars1* (Liu et al., 2014)
CARS1	• Microcephaly, developmental delay, and brittle hair syndrome (MDBH) [OMIM #618891] (Kuo et al., 2019)	***C. elegans***<br>• RNAi *cars-1* knockdown (Zheng et al., 2022)
DARS1	• Hypomyelination with brainstem and spinal cord involvement and leg spasticity (HBSL) [OMIM #615281] (Taft et al., 2013; Wolf et al., 2015)	***C. elegans***<br>• RNAi *dars-1* knockdown (Zheng et al., 2022)<br>**Mouse (*Mus musculus*)**<br>• Homozygous and heterozygous *Dars1* knockout (Fröhlich et al., 2017)<br>• Introduction of patient point mutations into the mouse *Dars1* gene (homozygous and *in trans* to null allele) (Fröhlich et al., 2020; Klugmann et al., 2022)
EPRS1	• Hypomyelinating leukodystrophy 15; HLD15 [OMIM #617951] (Mendes et al., 2018)<br>• Psychomotor developmental delay, epilepsy and deafness (Jin et al., 2022)	***C. elegans***<br>• RNAi *ears-1* and *pars-1* knockdown (Zheng et al., 2022)<br>**Mouse (*Mus musculus*)**<br>• *Eprs1* knockout (IMPC data) (Dickinson et al., 2016)<br>• Knock-in of phospho-deficient and phospho-mimetic *Eprs1* variants (targeting secondary enzyme functions) (Arif et al., 2017)
FARSA, FARSB	• Rajab interstitial lung disease with brain calcifications 1 [OMIM #613658] & 2 [OMIM #619013] (Antonellis et al., 2018; Zadjali et al., 2018; Krenke et al., 2019; Schuch et al., 2021; Charbit-Henrion et al., 2022; Kim et al., 2022)	***C. elegans***<br>• RNAi *fars-1* knockdown (Zheng et al., 2022)<br>***Drosophila melanogaster***<br>• Knockdown of *PheRS* and the individual subunits (α-*PheRs* and β-*PheRs*) (Ho et al., 2021)

		• Expression of "double-sieving" (amino acid recognition and editing) defective variants, RNAi knockdown of individual subunits (Lu et al., 2014)
GARS1	• Multisystem developmental disease (Oprescu et al., 2017)<br>• Systemic mitochondrial disease/mitochondrial respiratory chain disorder (McMillan et al., 2014; Taylor et al., 2014; Nafisinia et al., 2017a)	***C. elegans***<br>• RNAi *gars-1* knockdown (Zheng et al., 2022)<br>***Drosophila melanogaster***<br>• *GlyRS* variant identified in MARCM-based forward screen for mutants affecting dendritic and axonal development (Chihara et al., 2007)<br>**Zebrafish (*Danio rerio*)**<br>• Recessive lethal 's266' mutation (p.T209K; equivalent to p.T130K in human GlyRS) identified in an ENU screen (Malissovas et al., 2016)<br>**Mouse (*Mus musculus*)**<br>• Heterozygous and homozygous loss of function through gene trap insertion into intron 2 of *Gars1* (Seburn et al., 2006)
HARS1	• Usher Syndrome Type 3B [OMIM #614504] (Puffenberger et al., 2012)<br>• Multisystem ataxic syndrome (Galatolo et al., 2020)	***C. elegans***<br>• RNAi *hars-1* knockdown (Pierce et al., 2011; Zheng et al., 2022)<br>**Zebrafish (*Danio rerio*)**<br>• Morpholino knockdown of *hars1* (Waldron et al., 2019)<br>• *hars1* variant identified in mutagenesis screen for genes important for early development (Amsterdam et al., 2004)
IARS1	• Growth retardation, impaired intellectual development, hypotonia, and hepatopathy [OMIM #617093] (Kopajtich et al., 2016; Orenstein et al., 2017; Smigiel et al., 2017; Fagbemi et al., 2020; Zou et al., 2022)	***C. elegans***<br>• RNAi *iars-1* knockdown (Zheng et al., 2022)<br>**Zebrafish (*Danio rerio*)**<br>• Morpholino knockdown of *iars1* (Kopajtich et al., 2016)<br>• *iars1* variant identified from mutagenesis causing UPR and defects in angiogenesis (Castranova et al., 2016)<br>**Cattle (*Bos taurus*)**<br>• Homozygous missense mutation in *IARS1* causing weak calf syndrome discovered prior to human disease, produces similar phenotype (Hirano et al., 2013; Hirano et al., 2016)
KARS1	• Autosomal recessive deafness 89 [OMIM #613916] (Santos-Cortez et al., 2013)<br>• Congenital deafness and adult-onset progressive leukoencephalopathy [OMIM #619196] (Sun et al., 2019; van der Knaap et al., 2019)<br>• Infantile-onset progressive leukoencephalopathy, with or without deafness [OMIM #619147] (McMillan et al., 2015; Zhou et al., 2017; Ardissone et al., 2018; Ruzzenente et al., 2018; Itoh et al.,	***C. elegans***<br>• RNAi *kars-1* knockdown (Zheng et al., 2022)<br>***Xenopus laevis***<br>• Morpholino *kars1* knockdown and rescue analyses (Itoh et al., 2019)<br>**Zebrafish (*Danio rerio*)**<br>• *kars1* knockout using CRISPR/Cas9 (Lin et al., 2021)

	2019; Sun et al., 2019; Vargas et al., 2020; Lin et al., 2021; Murofushi et al., 2022)<br>• Charcot-Marie-Tooth disease, recessive intermediate B; CMTRIB [OMIM #613641] (McLaughlin et al., 2010)<br>• Hypertrophic cardiomyopathy and combined mitochondrial respiratory chain defect (Verrigni et al., 2017)<br>• Severe neurological and neurosensory disease with optic neuropathy (Scheidecker et al., 2019)<br>• Mitochondrial encephalohepatopathy, sensorineural hearing loss, leopard spot retinopathy and advanced liver disease (Peluso et al., 2021)	• *kars1* variant identified in mutagenesis screen for genes important for early development (Amsterdam et al., 2004)<br>**Mouse (*Mus musculus*)**<br>• ALS-causing SOD1 variant found to aberrantly interact with LysRS (particularly the mitochondrial form) (Kunst et al., 1997)<br>• *Kars1* knockout (IMPC data) (Dickinson et al., 2016)
LARS1	• Infantile liver syndrome 1 [OMIM #615438] (Casey et al., 2012; Lenz et al., 2020a; Hirata et al., 2021)	***C. elegans***<br>• RNAi *lars-1* knockdown (Zheng et al., 2022)<br>**Zebrafish (*Danio rerio*)**<br>• *larsb* knockout using CRISPR/Cas9 (Wang et al., 2018b; Inoue et al., 2021)
MARS1	• Interstitial lung and liver disease [OMIM #615486] (van Meel et al., 2013; Sun et al., 2017; Abuduxikuer et al., 2018; Rips et al., 2018; La Fay et al., 2021)<br>• Pulmonary alveolar proteinosis (Hadchouel et al., 2015; Comisso et al., 2018; Alzaid et al., 2019; Lenz et al., 2020b)<br>• Trichothiodystrophy (Botta et al., 2021)<br>• Delayed motor development, spastic paraplegia and intellectual disability (Okamoto et al., 2022)	***C. elegans***<br>• RNAi *mars-1* knockdown (Zheng et al., 2022)<br>**Zebrafish (*Danio rerio*)**<br>• *mars1* variants identified in mutagenesis screen for genes important for early development (Amsterdam et al., 2004)
NARS1	• Neurodevelopmental Disorder with Microcephaly, Impaired Language, and Gait Abnormalities, autosomal recessive; NEDMILG [OMIM #619091] (Manole et al., 2020; Wang et al., 2020)	***C. elegans***<br>• RNAi *nars-1* knockdown (Zheng et al., 2022)<br>**Zebrafish (*Danio rerio*)**<br>• Microinjection of mutant human *NARS1* mRNA into zebrafish embryos (Manole et al., 2020)<br>**Mouse (*Mus musculus*)**<br>• *Nars1* knockout (IMPC data) (Dickinson et al., 2016)
QARS1	• Progressive microcephaly, with seizures and cerebral and cerebellar atrophy; MSCCA [OMIM #615760] (Zhang et al., 2014; Shen et al., 2020; Chan et al., 2022)<br>• Early-onset epileptic encephalopathy (Kodera et al., 2015)<br>• Severe growth deficiency, microcephaly & intellectual disability (Leshinsky-Silver et al., 2017)	***C. elegans***<br>• RNAi *qars-1* knockdown (Zheng et al., 2022)<br>***Drosophila melanogaster***<br>• *GlnRS* variant identified in MARCM-based forward screen for mutants affecting dendritic and axonal development (Chihara et al., 2007)<br>**Zebrafish (*Danio rerio*)**

		• *qars1* knockout by insertion of a gene-trap cassette through retroviral infection of embryos (Zhang et al., 2014)<br>**Mouse (*Mus musculus*)**<br>• *Qars1* knockout (IMPC data) (Dickinson et al., 2016)
RARS1	• Hypomyelinating leukodystrophy 9; HLD9 [OMIM #616140] (Wolf et al., 2014; Nafisinia et al., 2017b; Rezaei et al., 2019; Mendes et al., 2020)	***C. elegans***<br>• RNAi *rars-1* knockdown (Zheng et al., 2022)<br>***Drosophila melanogaster***<br>• *ArgRS* knockout using CRISPR/Cas9, expression of SUMO conjugation resistant *ArgRS* variant (Nayak et al., 2021)<br>**Mouse (*Mus musculus*)**<br>• *Rars1* knockout (IMPC data) (Dickinson et al., 2016)
SARS1	• Neurodevelopmental disorder with microcephaly, ataxia, and seizures; NEDMAS [OMIM #617709] (Musante et al., 2017; Bögershausen et al., 2022; Karaer et al., 2022)<br>• Neurodevelopmental delay, central deafness, cardiomyopathy and metabolic decompensation during fever (Ravel et al., 2021)<br>• Neurodevelopmental disorder with spastic paraplegia, ataxia and seizures, without microcephaly (Verdura et al., 2022)	***C. elegans***<br>• RNAi *sars-1* knockdown (Zheng et al., 2022)<br>**Zebrafish (*Danio rerio*)**<br>• Morpholino *sars1* knockdown and mRNA rescue experiments (Wang et al., 2018a)<br>• *sars1* variant identified in mutagenesis screen for genes important for early development (Amsterdam et al., 2004)<br>• *sars1* variants identified in mutagenesis screens for mutants affecting vascular development, mRNA rescue experiments (Jin et al., 2007; Fukui et al., 2009; Herzog et al., 2009)<br>• Expression of phospho-defective and phospho-mimetic *sars1* mutant mRNA to investigate secondary function in vascular development (Shi et al., 2020)<br>• Morpholino *sars1* knockdown highlighting role in vascular development, mRNA rescue experiments (Xu et al., 2012; Shi et al., 2014)<br>**Mouse (*Mus musculus*)**<br>• Cell graft of a phosphorylation-deficient, constitutively active *Sars1* variant to investigate effects on angiogenesis (Shi et al., 2020)
TARS1	• Nonphotosensitive trichothiodystrophy 7; TTD7 [OMIM #618546] (Theil et al., 2019)	***C. elegans***<br>• RNAi *tars-1* knockdown (Zheng et al., 2022)<br>**Zebrafish (*Danio rerio*)**<br>• *tars1* variant identified in a mutagenesis screen causing abnormal angiogenesis, mRNA rescue experiments (Cao et al., 2016; Zhang et al., 2021)<br>• *tars1* variant identified from mutagenesis causing UPR and defects in angiogenesis (Castranova et al., 2016)<br>• Morpholino *tars1* knockdown (Mirando et al., 2015; Jeong et al., 2019)

		**Mouse (*Mus musculus*)**<br>• Intramuscular *Tars1* knockdown using lentivirus following injury-induced muscle regeneration, investigating non-canonical function of ThrRS in myogenic differentiation (Dai et al., 2021)
VARS1	• Neurodevelopmental disorder with microcephaly, seizures, and cortical atrophy [OMIM #617802] (Karaca et al., 2015; Okur et al., 2018; Stephen et al., 2018; Friedman et al., 2019; Siekierska et al., 2019)	***C. elegans***<br>• Expression of partial loss-of-function mutation (Rastogi et al., 2015)<br>• RNAi *vars-2* knockdown (Zheng et al., 2022)<br>**Zebrafish (*Danio rerio*)**<br>• Transient overexpression of editing defective *vars1* variant mRNA (Song et al., 2016)<br>• *vars1* knockout using CRISPR/Cas9 (Siekierska et al., 2019)
WARS1	• Neurodevelopmental syndrome (Bögershausen et al., 2022; Lin et al., 2022; Okamoto et al., 2022)	***C. elegans***<br>• RNAi *wars-1* knockdown (Lin et al., 2022; Zheng et al., 2022)<br>***Drosophila melanogaster***<br>• *TrpRS* variant identified in MARCM-based forward screen for mutants affecting dendritic and axonal development (Chihara et al., 2007)<br>**Zebrafish**<br>• *Wars1* knockout using CRISPR/Cas9, mRNA rescue experiments (Bögershausen et al., 2022; Lin et al., 2022)
YARS1	• Infantile-onset multisystem neurologic, endocrine, and pancreatic disease 2 [OMIM #619418] (Nowaczyk et al., 2017; Williams et al., 2019; Averdunk et al., 2021; Estève et al., 2021; Zeiad et al., 2021)<br>• Multisystem disease including retinitis pigmentosa and deafness (Tracewska-Siemiątkowska et al., 2017)	***C. elegans***<br>• RNAi *yars-1* knockdown (Zheng et al., 2022)<br>**Mouse (*Mus musculus*)**<br>• *Yars1* knockout (IMPC data) (Dickinson et al., 2016)<br>• Heterozygous and homozygous loss of function through gene trap insertion into exon 11 of *Yars1* (Hines et al., 2021)

References

Abuduxikuer, K., Feng, J.Y., Lu, Y., Xie, X.B., Chen, L., and Wang, J.S. (2018). Novel methionyl-tRNA synthetase gene variants/phenotypes in interstitial lung and liver disease: A case report and review of literature. *World J Gastroenterol* 24(36)**,** 4208-4216. doi: 10.3748/wjg.v24.i36.4208.

Alzaid, M., Alshamrani, A., Al Harbi, A.S., Alenzi, A., and Mohamed, S. (2019). Methionyl-tRNA synthetase novel mutation causes pulmonary alveolar proteinosis. *Saudi Med J* 40(2)**,** 195-198. doi: 10.15537/smj.2019.2.23908.

Amsterdam, A., Nissen, R.M., Sun, Z., Swindell, E.C., Farrington, S., and Hopkins, N. (2004). Identification of 315 genes essential for early zebrafish development. *Proc Natl Acad Sci U S A* 101(35)**,** 12792-12797. doi: 10.1073/pnas.0403929101.

Antonellis, A., Oprescu, S.N., Griffin, L.B., Heider, A., Amalfitano, A., and Innis, J.W. (2018). Compound heterozygosity for loss-of-function FARSB variants in a patient with classic features of recessive aminoacyl-tRNA synthetase-related disease. *Hum Mutat* 39(6)**,** 834-840. doi: 10.1002/humu.23424.

Ardissone, A., Tonduti, D., Legati, A., Lamantea, E., Barone, R., Dorboz, I., et al. (2018). KARS-related diseases: progressive leukoencephalopathy with brainstem and spinal cord calcifications as new phenotype and a review of literature. *Orphanet J Rare Dis* 13(1)**,** 45. doi: 10.1186/s13023-018-0788-4.

Arif, A., Terenzi, F., Potdar, A.A., Jia, J., Sacks, J., China, A., et al. (2017). EPRS is a critical mTORC1-S6K1 effector that influences adiposity in mice. *Nature* 542(7641)**,** 357-361. doi: 10.1038/nature21380.

Averdunk, L., Sticht, H., Surowy, H., Lüdecke, H.J., Koch-Hogrebe, M., Alsaif, H.S., et al. (2021). The recurrent missense mutation p.(Arg367Trp) in YARS1 causes a distinct neurodevelopmental phenotype. *J Mol Med (Berl)* 99(12)**,** 1755-1768. doi: 10.1007/s00109-021-02124-9.

Bögershausen, N., Krawczyk, H.E., Jamra, R.A., Lin, S.J., Yigit, G., Hüning, I., et al. (2022). WARS1 and SARS1: Two tRNA synthetases implicated in autosomal recessive microcephaly. *Hum Mutat* 43(10)**,** 1454-1471. doi: 10.1002/humu.24430.

Botta, E., Theil, A.F., Raams, A., Caligiuri, G., Giachetti, S., Bione, S., et al. (2021). Protein instability associated with AARS1 and MARS1 mutations causes trichothiodystrophy. *Hum Mol Genet* 30(18)**,** 1711-1720. doi: 10.1093/hmg/ddab123.

Cao, Z., Wang, H., Mao, X., and Luo, L. (2016). Noncanonical function of threonyl-tRNA synthetase regulates vascular development in zebrafish. *Biochem Biophys Res Commun* 473(1)**,** 67-72. doi: 10.1016/j.bbrc.2016.03.051.

Casey, J.P., McGettigan, P., Lynam-Lennon, N., McDermott, M., Regan, R., Conroy, J., et al. (2012). Identification of a mutation in LARS as a novel cause of infantile hepatopathy. *Mol Genet Metab* 106(3), 351-358. doi: 10.1016/j.ymgme.2012.04.017.

Castranova, D., Davis, A.E., Lo, B.D., Miller, M.F., Paukstelis, P.J., Swift, M.R., et al. (2016). Aminoacyl-Transfer RNA Synthetase Deficiency Promotes Angiogenesis via the Unfolded Protein Response Pathway. *Arterioscler Thromb Vasc Biol* 36(4)**,** 655-662. doi: 10.1161/atvbaha.115.307087.

Chan, D.L., Rudinger-Thirion, J., Frugier, M., Riley, L.G., Ho, G., Kothur, K., et al. (2022). A case of QARS1 associated epileptic encephalopathy and review of epilepsy in aminoacyl-tRNA synthetase disorders. *Brain Dev* 44(2), 142-147. doi: 10.1016/j.braindev.2021.10.009.

Charbit-Henrion, F., Goguyer-Deschaumes, R., Borensztajn, K., Mirande, M., Berthelet, J., Rodrigues-Lima, F., et al. (2022). Systemic inflammatory syndrome in children with FARSA deficiency. *Clin Genet* 101(5-6), 552-558. doi: 10.1111/cge.14120.

Chihara, T., Luginbuhl, D., and Luo, L. (2007). Cytoplasmic and mitochondrial protein translation in axonal and dendritic terminal arborization. *Nat Neurosci* 10(7), 828-837. doi: 10.1038/nn1910.

Comisso, M., Hadchouel, A., de Blic, J., and Mirande, M. (2018). Mutations in MARS identified in a specific type of pulmonary alveolar proteinosis alter methionyl-tRNA synthetase activity. *Febs j* 285(14), 2654-2661. doi: 10.1111/febs.14510.

Dai, C., Reyes-Ordoñez, A., You, J.S., and Chen, J. (2021). A non-translational role of threonyl-tRNA synthetase in regulating JNK signaling during myogenic differentiation. *Faseb j* 35(10), e21948. doi: 10.1096/fj.202101094R.

Dickinson, M.E., Flenniken, A.M., Ji, X., Teboul, L., Wong, M.D., White, J.K., et al. (2016). High-throughput discovery of novel developmental phenotypes. *Nature* 537(7621), 508-514. doi: 10.1038/nature19356.

Estève, C., Roman, C., DeLeusse, C., Baravalle, M., Bertaux, K., Blanc, F., et al. (2021). Novel partial loss-of-function variants in the tyrosyl-tRNA synthetase 1 (YARS1) gene involved in multisystem disease. *Eur J Med Genet* 64(10), 104294. doi: 10.1016/j.ejmg.2021.104294.

Fagbemi, A., Newman, W.G., Tangye, S.G., Hughes, S.M., Cheesman, E., and Arkwright, P.D. (2020). Refractory very early-onset inflammatory bowel disease associated with cytosolic isoleucyl-tRNA synthetase deficiency: A case report. *World J Gastroenterol* 26(15), 1841-1846. doi: 10.3748/wjg.v26.i15.1841.

Friedman, J., Smith, D.E., Issa, M.Y., Stanley, V., Wang, R., Mendes, M.I., et al. (2019). Biallelic mutations in valyl-tRNA synthetase gene VARS are associated with a progressive neurodevelopmental epileptic encephalopathy. *Nat Commun* 10(1), 707. doi: 10.1038/s41467-018-07067-3.

Fröhlich, D., Mendes, M.I., Kueh, A.J., Bongers, A., Herold, M.J., Salomons, G.S., et al. (2020). A Hypomorphic Dars1 (D367Y) Model Recapitulates Key Aspects of the Leukodystrophy HBSL. *Front Cell Neurosci* 14, 625879. doi: 10.3389/fncel.2020.625879.

Fröhlich, D., Suchowerska, A.K., Spencer, Z.H., von Jonquieres, G., Klugmann, C.B., Bongers, A., et al. (2017). In vivocharacterization of the aspartyl-tRNA synthetase DARS: Homing in on the leukodystrophy HBSL. *Neurobiology of Disease* 97(Pt A), 24-35. doi: 10.1016/j.nbd.2016.10.008.

Fukui, H., Hanaoka, R., and Kawahara, A. (2009). Noncanonical activity of seryl-tRNA synthetase is involved in vascular development. *Circ Res* 104(11), 1253-1259. doi: 10.1161/circresaha.108.191189.

Galatolo, D., Kuo, M.E., Mullen, P., Meyer-Schuman, R., Doccini, S., Battini, R., et al. (2020). Bi-allelic mutations in HARS1 severely impair histidyl-tRNA synthetase expression and enzymatic activity causing a novel multisystem ataxic syndrome. *Hum Mutat* 41(7), 1232-1237. doi: 10.1002/humu.24024.

Hadchouel, A., Wieland, T., Griese, M., Baruffini, E., Lorenz-Depiereux, B., Enaud, L., et al. (2015). Biallelic Mutations of Methionyl-tRNA Synthetase Cause a Specific Type of Pulmonary Alveolar Proteinosis Prevalent on Réunion Island. *Am J Hum Genet* 96(5)**,** 826-831. doi: 10.1016/j.ajhg.2015.03.010.

Helman, G., Mendes, M.I., Nicita, F., Darbelli, L., Sherbini, O., Moore, T., et al. (2021). Expanded phenotype of AARS1-related white matter disease. *Genet Med*. doi: 10.1038/s41436-021-01286-8.

Herzog, W., Müller, K., Huisken, J., and Stainier, D.Y. (2009). Genetic evidence for a noncanonical function of seryl-tRNA synthetase in vascular development. *Circ Res* 104(11)**,** 1260-1266. doi: 10.1161/circresaha.108.191718.

Hines, T.J., Tadenev, A.L.D., Lone, M.A., Hatton, C.L., Bagasrawala, I., Stum, M.G., et al. (2021). Precision mouse models of Yars/dominant intermediate Charcot-Marie-Tooth disease type C and Sptlc1/hereditary sensory and autonomic neuropathy type 1. *J Anat*. doi: 10.1111/joa.13605.

Hirano, T., Kobayashi, N., Matsuhashi, T., Watanabe, D., Watanabe, T., Takasuga, A., et al. (2013). Mapping and exome sequencing identifies a mutation in the IARS gene as the cause of hereditary perinatal weak calf syndrome. *PLoS One* 8(5)**,** e64036. doi: 10.1371/journal.pone.0064036.

Hirano, T., Matsuhashi, T., Takeda, K., Hara, H., Kobayashi, N., Kita, K., et al. (2016). IARS mutation causes prenatal death in Japanese Black cattle. *Anim Sci J* 87(9)**,** 1178-1181. doi: 10.1111/asj.12639.

Hirata, K., Okamoto, N., Ichikawa, C., Inoue, S., Nozaki, M., Banno, K., et al. (2021). Severe course with lethal hepatocellular injury and skeletal muscular dysgenesis in a neonate with infantile liver failure syndrome type 1 caused by novel LARS1 mutations. *Am J Med Genet A* 185(3)**,** 866-870. doi: 10.1002/ajmg.a.62012.

Ho, M.T., Lu, J., Brunßen, D., and Suter, B. (2021). A translation-independent function of PheRS activates growth and proliferation in Drosophila. *Dis Model Mech* 14(3). doi: 10.1242/dmm.048132.

Inoue, M., Miyahara, H., Shiraishi, H., Shimizu, N., Tsumori, M., Kiyota, K., et al. (2021). Leucyl-tRNA synthetase deficiency systemically induces excessive autophagy in zebrafish. *Sci Rep* 11(1)**,** 8392. doi: 10.1038/s41598-021-87879-4.

Itoh, M., Dai, H., Horike, S.I., Gonzalez, J., Kitami, Y., Meguro-Horike, M., et al. (2019). Biallelic KARS pathogenic variants cause an early-onset progressive leukodystrophy. *Brain* 142(3)**,** 560-573. doi: 10.1093/brain/awz001.

Jeong, S.J., Park, S., Nguyen, L.T., Hwang, J., Lee, E.Y., Giong, H.K., et al. (2019). A threonyl-tRNA synthetase-mediated translation initiation machinery. *Nat Commun* 10(1)**,** 1357. doi: 10.1038/s41467-019-09086-0.

Jin, D., Wek, S.A., Cordova, R.A., Wek, R.C., Lacombe, D., Michaud, V., et al. (2022). Aminoacylation-defective bi-allelic mutations in human EPRS1 associated with psychomotor developmental delay, epilepsy, and deafness. *Clin Genet*. doi: 10.1111/cge.14269.

Jin, S.W., Herzog, W., Santoro, M.M., Mitchell, T.S., Frantsve, J., Jungblut, B., et al. (2007). A transgene-assisted genetic screen identifies essential regulators of vascular development in vertebrate embryos. *Dev Biol* 307(1)**,** 29-42. doi: 10.1016/j.ydbio.2007.03.526.

Karaca, E., Harel, T., Pehlivan, D., Jhangiani, S.N., Gambin, T., Coban Akdemir, Z., et al. (2015). Genes that Affect Brain Structure and Function Identified by Rare Variant Analyses of Mendelian Neurologic Disease. *Neuron* 88(3)**,** 499-513. doi: 10.1016/j.neuron.2015.09.048.

Karaer, K., Karaer, D., Yüksel, Z., and Işikay, S. (2022). Neurodevelopmental disorder with microcephaly, ataxia, and seizures syndrome: expansion of the clinical spectrum. *Clin Dysmorphol* 31(4)**,** 167-173. doi: 10.1097/mcd.0000000000000426.

Kim, S.Y., Ko, S., Kang, H., Kim, M.J., Moon, J., Lim, B.C., et al. (2022). Fatal systemic disorder caused by biallelic variants in FARSA. *Orphanet J Rare Dis* 17(1)**,** 306. doi: 10.1186/s13023-022-02457-9.

Klugmann, M., Kalotay, E., Delerue, F., Ittner, L.M., Bongers, A., Yu, J., et al. (2022). Developmental delay and late onset HBSL pathology in hypomorphic Dars1(M256L) mice. *Neurochem Res* 47(7)**,** 1972-1984. doi: 10.1007/s11064-022-03582-4.

Kodera, H., Osaka, H., Iai, M., Aida, N., Yamashita, A., Tsurusaki, Y., et al. (2015). Mutations in the glutaminyl-tRNA synthetase gene cause early-onset epileptic encephalopathy. *J Hum Genet* 60(2)**,** 97-101. doi: 10.1038/jhg.2014.103.

Kopajtich, R., Murayama, K., Janecke, A.R., Haack, T.B., Breuer, M., Knisely, A.S., et al. (2016). Biallelic IARS Mutations Cause Growth Retardation with Prenatal Onset, Intellectual Disability, Muscular Hypotonia, and Infantile Hepatopathy. *Am J Hum Genet* 99(2)**,** 414-422. doi: 10.1016/j.ajhg.2016.05.027.

Krenke, K., Szczaluba, K., Bielecka, T., Rydzanicz, M., Lange, J., Koppolu, A., et al. (2019). FARSA mutations mimic phenylalanyl-tRNA synthetase deficiency caused by FARSB defects. *Clin Genet* 96(5)**,** 468-472. doi: 10.1111/cge.13614.

Kunst, C.B., Mezey, E., Brownstein, M.J., and Patterson, D. (1997). Mutations in SOD1 associated with amyotrophic lateral sclerosis cause novel protein interactions. *Nat Genet* 15(1)**,** 91-94. doi: 10.1038/ng0197-91.

Kuo, M.E., Theil, A.F., Kievit, A., Malicdan, M.C., Introne, W.J., Christian, T., et al. (2019). Cysteinyl-tRNA Synthetase Mutations Cause a Multi-System, Recessive Disease That Includes Microcephaly, Developmental Delay, and Brittle Hair and Nails. *Am J Hum Genet* 104(3)**,** 520-529. doi: 10.1016/j.ajhg.2019.01.006.

La Fay, C., Hoebeke, C., Juzaud, M., Spraul, A., Heux, P., Dubus, J.C., et al. (2021). Deep phenotyping of MARS1 (interstitial lung and liver disease) and LARS1 (infantile liver failure syndrome 1) recessive multisystemic disease using Human Phenotype Ontology annotation: Overlap and differences. Case report and review of literature. *Eur J Med Genet* 64(11)**,** 104334. doi: 10.1016/j.ejmg.2021.104334.

Lee, J.W., Beebe, K., Nangle, L.A., Jang, J., Longo-Guess, C.M., Cook, S.A., et al. (2006). Editing-defective tRNA synthetase causes protein misfolding and neurodegeneration. *Nature* 443(7107)**,** 50-55. doi: 10.1038/nature05096.

Lenz, D., Smith, D.E.C., Crushell, E., Husain, R.A., Salomons, G.S., Alhaddad, B., et al. (2020a). Genotypic diversity and phenotypic spectrum of infantile liver failure syndrome type 1 due to variants in LARS1. *Genet Med* 22(11)**,** 1863-1873. doi: 10.1038/s41436-020-0904-4.

Lenz, D., Stahl, M., Seidl, E., Schöndorf, D., Brennenstuhl, H., Gesenhues, F., et al. (2020b). Rescue of respiratory failure in pulmonary alveolar proteinosis due to pathogenic MARS1 variants. *Pediatr Pulmonol* 55(11)**,** 3057-3066. doi: 10.1002/ppul.25031.

Leshinsky-Silver, E., Ling, J., Wu, J., Vinkler, C., Yosovich, K., Bahar, S., et al. (2017). Severe growth deficiency, microcephaly, intellectual disability, and characteristic facial features are due to a homozygous QARS mutation. *Neurogenetics* 18(3)**,** 141-146. doi: 10.1007/s10048-017-0516-6.

Lin, S.J., Vona, B., Barbalho, P.G., Kaiyrzhanov, R., Maroofian, R., Petree, C., et al. (2021). Biallelic variants in KARS1 are associated with neurodevelopmental disorders and hearing loss recapitulated by the knockout zebrafish. *Genet Med* 23(10)**,** 1933-1943. doi: 10.1038/s41436-021-01239-1.

Lin, S.J., Vona, B., Porter, H.M., Izadi, M., Huang, K., Lacassie, Y., et al. (2022). Biallelic variants in WARS1 cause a highly variable neurodevelopmental syndrome and implicate a critical exon for normal auditory function. *Hum Mutat* 43(10)**,** 1472-1489. doi: 10.1002/humu.24435.

Liu, Y., Satz, J.S., Vo, M.N., Nangle, L.A., Schimmel, P., and Ackerman, S.L. (2014). Deficiencies in tRNA synthetase editing activity cause cardioproteinopathy. *Proc Natl Acad Sci U S A* 111(49)**,** 17570-17575. doi: 10.1073/pnas.1420196111.

Lu, J., Bergert, M., Walther, A., and Suter, B. (2014). Double-sieving-defective aminoacyl-tRNA synthetase causes protein mistranslation and affects cellular physiology and development. *Nat Commun* 5**,** 5650. doi: 10.1038/ncomms6650.

Malissovas, N., Griffin, L.B., Antonellis, A., and Beis, D. (2016). Dimerization is required for GARS-mediated neurotoxicity in dominant CMT disease. *Hum Mol Genet* 25(8)**,** 1528-1542. doi: 10.1093/hmg/ddw031.

Manole, A., Efthymiou, S., O'Connor, E., Mendes, M.I., Jennings, M., Maroofian, R., et al. (2020). De Novo and Bi-allelic Pathogenic Variants in NARS1 Cause Neurodevelopmental Delay Due to Toxic Gain-of-Function and Partial Loss-of-Function Effects. *Am J Hum Genet* 107(2)**,** 311-324. doi: 10.1016/j.ajhg.2020.06.016.

Marten, L.M., Brinkert, F., Smith, D.E.C., Prokisch, H., Hempel, M., and Santer, R. (2020). Recurrent acute liver failure in alanyl-tRNA synthetase-1 (AARS1) deficiency. *Mol Genet Metab Rep* 25**,** 100681. doi: 10.1016/j.ymgmr.2020.100681.

McLaughlin, H.M., Sakaguchi, R., Liu, C., Igarashi, T., Pehlivan, D., Chu, K., et al. (2010). Compound heterozygosity for loss-of-function lysyl-tRNA synthetase mutations in a patient with peripheral neuropathy. *Am J Hum Genet* 87(4)**,** 560-566. doi: 10.1016/j.ajhg.2010.09.008.

McMillan, H.J., Humphreys, P., Smith, A., Schwartzentruber, J., Chakraborty, P., Bulman, D.E., et al. (2015). Congenital Visual Impairment and Progressive Microcephaly Due to Lysyl-Transfer Ribonucleic Acid (RNA) Synthetase (KARS) Mutations: The Expanding Phenotype of Aminoacyl-Transfer RNA Synthetase Mutations in Human Disease. *J Child Neurol* 30(8)**,** 1037-1043. doi: 10.1177/0883073814553272.

McMillan, H.J., Schwartzentruber, J., Smith, A., Lee, S., Chakraborty, P., Bulman, D.E., et al. (2014). Compound heterozygous mutations in glycyl-tRNA synthetase are a proposed cause of systemic mitochondrial disease. *BMC Med Genet* 15**,** 36. doi: 10.1186/1471-2350-15-36.

Mendes, M.I., Green, L.M.C., Bertini, E., Tonduti, D., Aiello, C., Smith, D., et al. (2020). RARS1-related hypomyelinating leukodystrophy: Expanding the spectrum. *Ann Clin Transl Neurol* 7(1)**,** 83-93. doi: 10.1002/acn3.50960.

Mendes, M.I., Gutierrez Salazar, M., Guerrero, K., Thiffault, I., Salomons, G.S., Gauquelin, L., et al. (2018). Bi-allelic Mutations in EPRS, Encoding the Glutamyl-Prolyl-Aminoacyl-tRNA Synthetase, Cause a Hypomyelinating Leukodystrophy. *Am J Hum Genet* 102(4), 676-684. doi: 10.1016/j.ajhg.2018.02.011.

Mirando, A.C., Fang, P., Williams, T.F., Baldor, L.C., Howe, A.K., Ebert, A.M., et al. (2015). Aminoacyl-tRNA synthetase dependent angiogenesis revealed by a bioengineered macrolide inhibitor. *Sci Rep* 5, 13160. doi: 10.1038/srep13160.

Murofushi, Y., Hayakawa, I., Abe, Y., Ohto, T., Murayama, K., Suzuki, H., et al. (2022). Ketogenic Diet for KARS-Related Mitochondrial Dysfunction and Progressive Leukodystrophy. *Neuropediatrics* 53(1), 65-68. doi: 10.1055/s-0041-1732446.

Musante, L., Püttmann, L., Kahrizi, K., Garshasbi, M., Hu, H., Stehr, H., et al. (2017). Mutations of the aminoacyl-tRNA-synthetases SARS and WARS2 are implicated in the etiology of autosomal recessive intellectual disability. *Hum Mutat* 38(6), 621-636. doi: 10.1002/humu.23205.

Nafisinia, M., Riley, L.G., Gold, W.A., Bhattacharya, K., Broderick, C.R., Thorburn, D.R., et al. (2017a). Compound heterozygous mutations in glycyl-tRNA synthetase (GARS) cause mitochondrial respiratory chain dysfunction. *PLoS One* 12(6), e0178125. doi: 10.1371/journal.pone.0178125.

Nafisinia, M., Sobreira, N., Riley, L., Gold, W., Uhlenberg, B., Weiss, C., et al. (2017b). Mutations in RARS cause a hypomyelination disorder akin to Pelizaeus-Merzbacher disease. *European Journal of Human Genetics* 25(10), 1134-1141. doi: 10.1038/ejhg.2017.119.

Nakayama, T., Wu, J., Galvin-Parton, P., Weiss, J., Andriola, M.R., Hill, R.S., et al. (2017). Deficient activity of alanyl-tRNA synthetase underlies an autosomal recessive syndrome of progressive microcephaly, hypomyelination, and epileptic encephalopathy. *Hum Mutat* 38(10), 1348-1354. doi: 10.1002/humu.23250.

Nayak, P., Kejriwal, A., and Ratnaparkhi, G.S. (2021). SUMOylation of Arginyl tRNA Synthetase Modulates the Drosophila Innate Immune Response. *Front Cell Dev Biol* 9, 695630. doi: 10.3389/fcell.2021.695630.

Nowaczyk, M.J., Huang, L., Tarnopolsky, M., Schwartzentruber, J., Majewski, J., Bulman, D.E., et al. (2017). A novel multisystem disease associated with recessive mutations in the tyrosyl-tRNA synthetase (YARS) gene. *Am J Med Genet A* 173(1), 126-134. doi: 10.1002/ajmg.a.37973.

Okamoto, N., Miya, F., Tsunoda, T., Kanemura, Y., Saitoh, S., Kato, M., et al. (2022). Four pedigrees with aminoacyl-tRNA synthetase abnormalities. *Neurol Sci* 43(4), 2765-2774. doi: 10.1007/s10072-021-05626-z.

Okur, V., Ganapathi, M., Wilson, A., and Chung, W.K. (2018). Biallelic variants in VARS in a family with two siblings with intellectual disability and microcephaly: case report and review of the literature. *Cold Spring Harb Mol Case Stud* 4(5). doi: 10.1101/mcs.a003301.

Oprescu, S.N., Chepa-Lotrea, X., Takase, R., Golas, G., Markello, T.C., Adams, D.R., et al. (2017). Compound heterozygosity for loss-of-function GARS variants results in a multisystem developmental syndrome that includes severe growth retardation. *Hum Mutat* 38(10), 1412-1420. doi: 10.1002/humu.23287.

Orenstein, N., Weiss, K., Oprescu, S.N., Shapira, R., Kidron, D., Vanagaite-Basel, L., et al. (2017). Bi-allelic IARS mutations in a child with intra-uterine growth retardation, neonatal cholestasis, and mild developmental delay. *Clin Genet* 91(6), 913-917. doi: 10.1111/cge.12930.

Peluso, F., Palazzo, V., Indolfi, G., Mari, F., Pasqualetti, R., Procopio, E., et al. (2021). Leopard-like retinopathy and severe early-onset portal hypertension expand the phenotype of KARS1-related syndrome: a case report. *BMC Med Genomics* 14(1), 25. doi: 10.1186/s12920-020-00863-1.

Pierce, S.B., Chisholm, K.M., Lynch, E.D., Lee, M.K., Walsh, T., Opitz, J.M., et al. (2011). Mutations in mitochondrial histidyl tRNA synthetase HARS2 cause ovarian dysgenesis and sensorineural hearing loss of Perrault syndrome. *Proc Natl Acad Sci U S A* 108(16), 6543-6548. doi: 10.1073/pnas.1103471108.

Puffenberger, E.G., Jinks, R.N., Sougnez, C., Cibulskis, K., Willert, R.A., Achilly, N.P., et al. (2012). Genetic mapping and exome sequencing identify variants associated with five novel diseases. *PLoS One* 7(1), e28936. doi: 10.1371/journal.pone.0028936.

Rastogi, S., Borgo, B., Pazdernik, N., Fox, P., Mardis, E.R., Kohara, Y., et al. (2015). Caenorhabditis elegans glp-4 Encodes a Valyl Aminoacyl tRNA Synthetase. *G3 (Bethesda)* 5(12), 2719-2728. doi: 10.1534/g3.115.021899.

Ravel, J.M., Dreumont, N., Mosca, P., Smith, D.E.C., Mendes, M.I., Wiedemann, A., et al. (2021). A bi-allelic loss-of-function SARS1 variant in children with neurodevelopmental delay, deafness, cardiomyopathy, and decompensation during fever. *Hum Mutat* 42(12), 1576-1583. doi: 10.1002/humu.24285.

Rezaei, Z., Hosseinpour, S., Ashrafi, M.R., Mahdieh, N., Alizadeh, H., Mohammadpour, M., et al. (2019). Hypomyelinating Leukodystrophy with Spinal Cord Involvement Caused by a Novel Variant in RARS: Report of Two Unrelated Patients. *Neuropediatrics* 50(2), 130-134. doi: 10.1055/s-0039-1679911.

Rips, J., Meyer-Schuman, R., Breuer, O., Tsabari, R., Shaag, A., Revel-Vilk, S., et al. (2018). MARS variant associated with both recessive interstitial lung and liver disease and dominant Charcot-Marie-Tooth disease. *Eur J Med Genet* 61(10), 616-620. doi: 10.1016/j.ejmg.2018.04.005.

Ruzzenente, B., Assouline, Z., Barcia, G., Rio, M., Boddaert, N., Munnich, A., et al. (2018). Inhibition of mitochondrial translation in fibroblasts from a patient expressing the KARS p.(Pro228Leu) variant and presenting with sensorineural deafness, developmental delay, and lactic acidosis. *Hum Mutat* 39(12), 2047-2059. doi: 10.1002/humu.23657.

Santos-Cortez, R.L., Lee, K., Azeem, Z., Antonellis, P.J., Pollock, L.M., Khan, S., et al. (2013). Mutations in KARS, encoding lysyl-tRNA synthetase, cause autosomal-recessive nonsyndromic hearing impairment DFNB89. *Am J Hum Genet* 93(1), 132-140. doi: 10.1016/j.ajhg.2013.05.018.

Scheidecker, S., Bär, S., Stoetzel, C., Geoffroy, V., Lannes, B., Rinaldi, B., et al. (2019). Mutations in KARS cause a severe neurological and neurosensory disease with optic neuropathy. *Hum Mutat* 40(10), 1826-1840. doi: 10.1002/humu.23799.

Schuch, L.A., Forstner, M., Rapp, C.K., Li, Y., Smith, D.E.C., Mendes, M.I., et al. (2021). FARS1-related disorders caused by bi-allelic mutations in cytosolic phenylalanyl-tRNA synthetase genes: Look beyond the lungs! *Clin Genet* 99(6)**,** 789-801. doi: 10.1111/cge.13943.

Seburn, K.L., Nangle, L.A., Cox, G.A., Schimmel, P., and Burgess, R.W. (2006). An active dominant mutation of glycyl-tRNA synthetase causes neuropathy in a Charcot-Marie-Tooth 2D mouse model. *Neuron* 51(6)**,** 715-726. doi: 10.1016/j.neuron.2006.08.027.

Shen, Y.W., Weng, Z.F., He, W., Chen, Y.H., Wang, Q.H., Zou, L.P., et al. (2020). [QARS1 gene related glutaminyl-tRNA synthetase deficiency syndrome: report of three cases and a review of literature]. *Zhonghua Er Ke Za Zhi* 58(12)**,** 1006-1012. doi: 10.3760/cma.j.cn112140-20200603-00571.

Shi, Y., Liu, Z., Zhang, Q., Vallee, I., Mo, Z., Kishi, S., et al. (2020). Phosphorylation of seryl-tRNA synthetase by ATM/ATR is essential for hypoxia-induced angiogenesis. *PLoS Biol* 18(12)**,** e3000991. doi: 10.1371/journal.pbio.3000991.

Shi, Y., Xu, X., Zhang, Q., Fu, G., Mo, Z., Wang, G.S., et al. (2014). tRNA synthetase counteracts c-Myc to develop functional vasculature. *Elife* 3**,** e02349. doi: 10.7554/eLife.02349.

Siekierska, A., Stamberger, H., Deconinck, T., Oprescu, S.N., Partoens, M., Zhang, Y., et al. (2019). Biallelic VARS variants cause developmental encephalopathy with microcephaly that is recapitulated in vars knockout zebrafish. *Nat Commun* 10(1)**,** 708. doi: 10.1038/s41467-018-07953-w.

Simons, C., Griffin, L.B., Helman, G., Golas, G., Pizzino, A., Bloom, M., et al. (2015). Loss-of-function alanyl-tRNA synthetase mutations cause an autosomal-recessive early-onset epileptic encephalopathy with persistent myelination defect. *Am J Hum Genet* 96(4)**,** 675-681. doi: 10.1016/j.ajhg.2015.02.012.

Smigiel, R., Biela, M., Biernacka, A., Stembalska, A., Sasiadek, M., Kosinska, J., et al. (2017). New evidence for association of recessive IARS gene mutations with hepatopathy, hypotonia, intellectual disability and growth retardation. *Clin Genet* 92(6)**,** 671-673. doi: 10.1111/cge.13080.

Song, Y., Shi, Y., Carland, T.M., Lian, S., Sasaki, T., Schork, N.J., et al. (2016). p53-Dependent DNA damage response sensitive to editing-defective tRNA synthetase in zebrafish. *Proc Natl Acad Sci U S A* 113(30)**,** 8460-8465. doi: 10.1073/pnas.1608139113.

Stephen, J., Nampoothiri, S., Banerjee, A., Tolman, N.J., Penninger, J.M., Elling, U., et al. (2018). Loss of function mutations in VARS encoding cytoplasmic valyl-tRNA synthetase cause microcephaly, seizures, and progressive cerebral atrophy. *Hum Genet* 137(4)**,** 293-303. doi: 10.1007/s00439-018-1882-3.

Sun, C., Song, J., Jiang, Y., Zhao, C., Lu, J., Li, Y., et al. (2019). Loss-of-function mutations in Lysyl-tRNA synthetase cause various leukoencephalopathy phenotypes. *Neurol Genet* 5(2)**,** e565. doi: 10.1212/nxg.0000000000000316.

Sun, Y., Hu, G., Luo, J., Fang, D., Yu, Y., Wang, X., et al. (2017). Mutations in methionyl-tRNA synthetase gene in a Chinese family with interstitial lung and liver disease, postnatal growth failure and anemia. *J Hum Genet* 62(6)**,** 647-651. doi: 10.1038/jhg.2017.10.

Taft, R.J., Vanderver, A., Leventer, R.J., Damiani, S.A., Simons, C., Grimmond, S.M., et al. (2013). Mutations in DARS cause hypomyelination with brain stem and spinal cord involvement and leg spasticity. *Am J Hum Genet* 92(5), 774-780. doi: 10.1016/j.ajhg.2013.04.006.

Taylor, R.W., Pyle, A., Griffin, H., Blakely, E.L., Duff, J., He, L., et al. (2014). Use of whole-exome sequencing to determine the genetic basis of multiple mitochondrial respiratory chain complex deficiencies. *Jama* 312(1), 68-77. doi: 10.1001/jama.2014.7184.

Theil, A.F., Botta, E., Raams, A., Smith, D.E.C., Mendes, M.I., Caligiuri, G., et al. (2019). Bi-allelic TARS Mutations Are Associated with Brittle Hair Phenotype. *Am J Hum Genet* 105(2), 434-440. doi: 10.1016/j.ajhg.2019.06.017.

Tracewska-Siemiątkowska, A., Haer-Wigman, L., Bosch, D.G.M., Nickerson, D., Bamshad, M.J., van de Vorst, M., et al. (2017). An Expanded Multi-Organ Disease Phenotype Associated with Mutations in YARS. *Genes (Basel)* 8(12). doi: 10.3390/genes8120381.

van der Knaap, M.S., Bugiani, M., Mendes, M.I., Riley, L.G., Smith, D.E.C., Rudinger-Thirion, J., et al. (2019). Biallelic variants in LARS2 and KARS cause deafness and (ovario)leukodystrophy. *Neurology* 92(11), e1225-e1237. doi: 10.1212/wnl.0000000000007098.

van Meel, E., Wegner, D.J., Cliften, P., Willing, M.C., White, F.V., Kornfeld, S., et al. (2013). Rare recessive loss-of-function methionyl-tRNA synthetase mutations presenting as a multi-organ phenotype. *BMC Med Genet* 14, 106. doi: 10.1186/1471-2350-14-106.

Vargas, A., Rojas, J., Aivasovsky, I., Vergara, S., Castellanos, M., Prieto, C., et al. (2020). Progressive Early-Onset Leukodystrophy Related to Biallelic Variants in the KARS Gene: The First Case Described in Latin America. *Genes (Basel)* 11(12). doi: 10.3390/genes11121437.

Verdura, E., Senger, B., Raspall-Chaure, M., Schlüter, A., Launay, N., Ruiz, M., et al. (2022). Loss of seryl-tRNA synthetase (SARS1) causes complex spastic paraplegia and cellular senescence. *J Med Genet* 59(12), 1227-1233. doi: 10.1136/jmg-2022-108529.

Verrigni, D., Diodato, D., Di Nottia, M., Torraco, A., Bellacchio, E., Rizza, T., et al. (2017). Novel mutations in KARS cause hypertrophic cardiomyopathy and combined mitochondrial respiratory chain defect. *Clin Genet* 91(6), 918-923. doi: 10.1111/cge.12931.

Vo, M.N., Terrey, M., Lee, J.W., Roy, B., Moresco, J.J., Sun, L., et al. (2018). ANKRD16 prevents neuron loss caused by an editing-defective tRNA synthetase. *Nature* 557(7706), 510-515. doi: 10.1038/s41586-018-0137-8.

Waldron, A., Wilcox, C., Francklyn, C., and Ebert, A. (2019). Knock-Down of Histidyl-tRNA Synthetase Causes Cell Cycle Arrest and Apoptosis of Neuronal Progenitor Cells in vivo. *Front Cell Dev Biol* 7, 67. doi: 10.3389/fcell.2019.00067.

Wang, K., Zhao, S., Liu, B., Zhang, Q., Li, Y., Liu, J., et al. (2018a). Perturbations of BMP/TGF-β and VEGF/VEGFR signalling pathways in non-syndromic sporadic brain arteriovenous malformations (BAVM). *J Med Genet* 55(10), 675-684. doi: 10.1136/jmedgenet-2017-105224.

Wang, L., Li, Z., Sievert, D., Smith, D.E.C., Mendes, M.I., Chen, D.Y., et al. (2020). Loss of NARS1 impairs progenitor proliferation in cortical brain organoids and leads to microcephaly. *Nat Commun* 11(1), 4038. doi: 10.1038/s41467-020-17454-4.

Wang, Z., Song, J., Luo, L., and Ma, J. (2018b). Loss of Leucyl-tRNA synthetase b leads to ILFS1-like symptoms in zebrafish. *Biochem Biophys Res Commun* 505(2), 378-384. doi: 10.1016/j.bbrc.2018.09.133.

Williams, K.B., Brigatti, K.W., Puffenberger, E.G., Gonzaga-Jauregui, C., Griffin, L.B., Martinez, E.D., et al. (2019). Homozygosity for a mutation affecting the catalytic domain of tyrosyl-tRNA synthetase (YARS) causes multisystem disease. *Hum Mol Genet* 28(4)**,** 525-538. doi: 10.1093/hmg/ddy344.

Wolf, N.I., Salomons, G.S., Rodenburg, R.J., Pouwels, P.J., Schieving, J.H., Derks, T.G., et al. (2014). Mutations in RARS cause hypomyelination. *Ann Neurol* 76(1)**,** 134-139. doi: 10.1002/ana.24167.

Wolf, N.I., Toro, C., Kister, I., Latif, K.A., Leventer, R., Pizzino, A., et al. (2015). DARS-associated leukoencephalopathy can mimic a steroid-responsive neuroinflammatory disorder. *Neurology* 84(3)**,** 226-230. doi: 10.1212/WNL.0000000000001157.

Xu, X., Shi, Y., Zhang, H.M., Swindell, E.C., Marshall, A.G., Guo, M., et al. (2012). Unique domain appended to vertebrate tRNA synthetase is essential for vascular development. *Nat Commun* 3**,** 681. doi: 10.1038/ncomms1686.

Zadjali, F., Al-Yahyaee, A., Al-Nabhani, M., Al-Mubaihsi, S., Gujjar, A., Raniga, S., et al. (2018). Homozygosity for FARSB mutation leads to Phe-tRNA synthetase-related disease of growth restriction, brain calcification, and interstitial lung disease. *Hum Mutat* 39(10)**,** 1355-1359. doi: 10.1002/humu.23595.

Zeiad, R., Ferren, E.C., Young, D.D., De Lancy, S.J., Dedousis, D., Schillaci, L.A., et al. (2021). A Novel Homozygous Missense Mutation in the YARS Gene: Expanding the Phenotype of YARS Multisystem Disease. *J Endocr Soc* 5(2)**,** bvaa196. doi: 10.1210/jendso/bvaa196.

Zhang, F., Zeng, Q.Y., Xu, H., Xu, A.N., Liu, D.J., Li, N.Z., et al. (2021). Selective and competitive functions of the AAR and UPR pathways in stress-induced angiogenesis. *Cell Discov* 7(1)**,** 98. doi: 10.1038/s41421-021-00332-8.

Zhang, X., Ling, J., Barcia, G., Jing, L., Wu, J., Barry, B.J., et al. (2014). Mutations in QARS, encoding glutaminyl-tRNA synthetase, cause progressive microcephaly, cerebral-cerebellar atrophy, and intractable seizures. *Am J Hum Genet* 94(4)**,** 547-558. doi: 10.1016/j.ajhg.2014.03.003.

Zheng, T., Luo, Q., Han, C., Zhou, J., Gong, J., Chun, L., et al. (2022). Cytoplasmic and mitochondrial aminoacyl-tRNA synthetases differentially regulate lifespan in Caenorhabditis elegans. *iScience* 25(11)**,** 105266. doi: 10.1016/j.isci.2022.105266.

Zhou, X.L., He, L.X., Yu, L.J., Wang, Y., Wang, X.J., Wang, E.D., et al. (2017). Mutations in KARS cause early-onset hearing loss and leukoencephalopathy: Potential pathogenic mechanism. *Hum Mutat* 38(12)**,** 1740-1750. doi: 10.1002/humu.23335.

Zou, T.T., Sun, H.Q., Zhu, Y., He, T.T., Ling, W.W., Zhu, H.M., et al. (2022). Compound heterozygous variations in IARS1 cause recurrent liver failure and growth retardation in a Chinese patient: a case report. *BMC Pediatr* 22(1)**,** 329. doi: 10.1186/s12887-022-03371-6.
