# Supplementary material for: Recessive aminoacyl-tRNA synthetase disorders: lessons learned from in vivo disease models
Source: Front Neurosci. 2023 May 9;17:1182874. doi: 10.3389/fnins.2023.1182874 (PMC10234152; doi:10.3389/fnins.2023.1182874)
Supplement: Supplementary file 2 [file Table_2.pdf]

Supplementary Table 2. Diseases caused by recessive mutations in mitochondrial ARSs and associated animal studies. Some mouse ARS knockout models were generated and phenotyped by the International Mouse Phenotyping Consortium (IMPC), with data available at www.mousephenotype.org. Note that in *C. elegans*, the genes corresponding to the mitochondrial alanyl- and valyl-tRNA synthetase genes are *aars-1* and *vars-1*. Other *C. elegans* genes follow the standard pattern of nomenclature, where cytosolic and bifunctional ARS genes are assigned the suffix *-1* and mitochondrial genes are assigned the suffix *-2*.

Human ARS gene	Clinical presentation(s)	Animal studies and disease models
AARS2	• Combined oxidative phosphorylation deficiency 8 [OMIM #614096]; (Gotz et al., 2011; Taylor et al., 2014; Mazurova et al., 2017; Sommerville et al., 2019; Nielsen et al., 2020; van Helden et al., 2021)<br>• Progressive leukoencephalopathy with ovarian failure [OMIM #615889] (Dallabona et al., 2014; Zhou et al., 2019; Fan et al., 2022)<br>• Leukodystrophy without ovarian failure (Sun et al., 2017; Srivastava et al., 2019; Wang et al., 2019; Axelsen et al., 2021)<br>• Early-onset epileptic encephalopathy with hypomyelination (Simons et al., 2015; Nakayama et al., 2017)<br>• Adult-onset leukoencephalopathy with axonal spheroids and pigmented glia *(prev. known as hereditary diffuse leukoencephalopathy with axonal spheroids (HDLS) or pigmentary orthochromatic leukodystrophy (POLD))* (Lynch et al., 2016; Taglia et al., 2018)<br>• Primary pulmonary hypoplasia (Kiraly-Borri et al., 2019)<br>• Ataxia without leukoencephalopathy (Srivastava et al., 2019; Kuo et al., 2020; Okamoto et al., 2022)<br>• Retinopathy and optic atrophy (Peragallo et al., 2018)	***C. elegans***<br>• RNAi *aars-1* knockdown (Zheng et al., 2022a)<br>**Mouse (*Mus musculus*)**<br>• Introduction of editing defective *Aars2* variants (Hilander et al., 2018)<br>• *Aars2* knockout (IMPC data) (Dickinson et al., 2016)
CARS2	• Combined oxidative phosphorylation deficiency 27 [OMIM #616672] (Hallmann et al., 2014; Coughlin et al., 2015; Samanta et al., 2018; Kapoor et al., 2021; Li et al., 2021b)	***C. elegans***<br>• RNAi bifunctional *cars-1* knockdown (Zheng et al., 2022a)<br>**Zebrafish (*Danio rerio*)**<br>• Morpholino *cars2* knockdown (Wang et al., 2015)<br>**Mouse (*Mus musculus*)**<br>• Homozygous and heterozygous *Cars2* knockout (Akaike et al., 2017)
DARS2	• Leukoencephalopathy with brain stem and spinal cord involvement and lactate elevation; LBSL [OMIM #611105] (van der Knaap et al., 2003; Petzold et al., 2006; Scheper et al., 2007; Uluc et al., 2008; Isohanni et al., 2010; Lin et al., 2010; Labauge et al., 2011; Mierzewska et al., 2011; Miyake et al., 2011; Orcesi et al., 2011; Sharma et al., 2011; Steenweg et al., 2011; Synofzik et al., 2011; Steenweg et al., 2012b; Tzoulis et al., 2012; Cheng et al., 2013; Martikainen et al.,	***C. elegans***<br>• RNAi *dars-2* knockdown (Zheng et al., 2022a)<br>**Mouse (*Mus musculus*)**<br>• Conditional *Dars2* knockout in neurons (Aradjanski et al., 2017; Nemeth et al., 2020; Rumyantseva et al., 2020)<br>• Conditional *Dars2* knockout in oligodendrocytes (Aradjanski et al., 2017)

	2013; Yamashita et al., 2013; Tylki-Szymanska et al., 2014; van Berge et al., 2014; Köhler et al., 2015; Lan et al., 2017; Çavuşoğlu et al., 2018; Yahia et al., 2018; Lin et al., 2019; Yelam et al., 2019; Al Balushi et al., 2020; Felhi et al., 2020; Yazici Gencdal et al., 2020; Axelsen et al., 2021; Li et al., 2021c; Ngo et al., 2021; Roux et al., 2021; Stellingwerff et al., 2021; Wongkittichote et al., 2022)	• Conditional *Dars2* knockout in heart and skeletal muscle (Dogan et al., 2014)<br>• Heterozygous and homozygous *Dars2* knockout (Dickinson et al., 2016; Aradjanski et al., 2017)
EARS2	• Leukoencephalopathy With Thalamus and Brainstem Involvement and High Lactate; LTBL [OMIM #614924] (Steenweg et al., 2012a; Biancheri et al., 2015; Danhauser et al., 2016; Güngör et al., 2016; Kevelam et al., 2016; Şahin et al., 2016; Taskin et al., 2016; Oliveira et al., 2017; Sellars et al., 2017; Al Balushi et al., 2020; Barbosa-Gouveia et al., 2020; Felhi et al., 2020; Ni et al., 2021; Roux et al., 2021; Sawada et al., 2021)	***C. elegans***<br>• RNAi *ears-2* knockdown (Zheng et al., 2022a)<br>**Mouse (*Mus musculus*)**<br>• *Ears2* knockout (IMPC data) (Dickinson et al., 2016)
FARS2	• Combined oxidative phosphorylation deficiency 14 [OMIM #613658]; mitochondrial encephalopathy (Elo et al., 2012; Shamseldin et al., 2012; Almalki et al., 2014; Vernon et al., 2015; Walker et al., 2016; Almannai et al., 2018; Ville et al., 2020; Barcia et al., 2021b; Guerrero and Bhatia, 2021; Li et al., 2021b; Roux et al., 2021)<br>• Juvenile onset refractory epilepsy (Hotait et al., 2020)<br>• Autosomal recessive spastic paraplegia 77 [OMIM #617046] (Raviglione et al., 2016; Yang et al., 2016; Vantroys et al., 2017; Almannai et al., 2018; Forman et al., 2019; Meszarosova et al., 2020)	***C. elegans***<br>• RNAi *fars-2* knockdown (Zheng et al., 2022a)<br>***Drosophila melanogaster***<br>• *PheRS-m* knockout using CRISPR/Cas9 (Fan et al., 2021)<br>• RNAi *PheRS-m* knockdown (Fan et al., 2021)<br>• Introduction of loss-of-function missense mutations into *PheRS-m* (Mo et al., 2023)<br>**Zebrafish (*Danio rerio*)**<br>• Morpholino *fars2* knockdown (Li et al., 2021a; Chen et al., 2022)<br>**Mouse (*Mus musculus*)**<br>• *Fars2* knockout (IMPC data) (Dickinson et al., 2016)<br>• Introduction of human patient mutation into *Fars2* (Chen et al., 2022)<br>• *Fars2 knockout using CRISPR/Cas9* (Chen et al., 2022)<br>• Conditional *Fars2* knockout in neurons (Chen et al., 2022)
HARS2	• Perrault Syndrome 2 [OMIM #614926] (Pierce et al., 2011; Lerat et al., 2016; Demain et al., 2020; Yu et al., 2020; Zou et al., 2020; Souissi et al., 2021)	***C. elegans***<br>• RNAi bifunctional *hars-1* knockdown (Pierce et al., 2011; Zheng et al., 2022a)<br>**Zebrafish (*Danio rerio*)**<br>• Morpholino knockdown of bifunctional *hars1* (Waldron et al., 2019)<br>• *hars1* variants identified in mutagenesis screen for genes important for early development (Amsterdam et al., 2004)<br>**Mouse (*Mus musculus*)**<br>• Conditional *Hars2* knockout in hair cells (Xu et al., 2021)<br>• *Hars2* knockout (IMPC data) (Dickinson et al., 2016)

IARS2	• Cataracts, growth hormone deficiency, sensory neuropathy, sensorineural hearing loss, and skeletal dysplasia; CAGSSS [OMIM #616007] (Liberfarb et al., 1993; Schwartzentruber et al., 2014; Jabbour and Harissi-Dagher, 2016; Moosa et al., 2017; Vona et al., 2018; Lee et al., 2020)<br>• Leigh syndrome, West syndrome & CAGSSS (Takezawa et al., 2018)<br>• Infantile spasms, Leigh disease and Wolff-Parkinson White pattern (Upadia et al., 2022)<br>• Sideroblastic anemia (Barcia et al., 2021a; Gong et al., 2022), with hypoparathyroidism (Gong et al., 2022)	***C. elegans***<br>• RNAi *iars-2* knockdown (Zheng et al., 2022a)<br>**Mouse (*Mus musculus*)**<br>• *Iars2* knockout (IMPC data) (Dickinson et al., 2016)
LARS2	• Hydrops, lactic acidosis, and sideroblastic anemia; HLASA [OMIM #617021] (Riley et al., 2016; Riley et al., 2020)<br>• Perrault syndrome 4 [OMIM #615300] (Pierce et al., 2013; Lerat et al., 2016; Soldà et al., 2016; Demain et al., 2017; Kosaki et al., 2018; Al-Jaroudi et al., 2019; van der Knaap et al., 2019; Carminho-Rodrigues et al., 2020; Pan et al., 2020; Riley et al., 2020; Tucker et al., 2020; Buonfiglio et al., 2022; Sun et al., 2022)<br>• Premature ovarian insufficiency without hearing loss (Neyroud et al., 2022)<br>• Reversible mitochondrial myopathy, lactic acidosis and developmental delay (Riley et al., 2020)	***C. elegans***<br>• Expression of protein-truncating *lars-2* variant (Pierce et al., 2013)<br>• *lars-2* variant identified in genetic screen for genes affecting *C. elegans* lifespan (Lee et al., 2003)<br>• RNAi *lars-2* knockdown (Zheng et al., 2022a)
MARS2	• Combined oxidative phosphorylation deficiency 25 [OMIM #616430] (Webb et al., 2015)<br>• Autosomal recessive spastic ataxia with leukoencephalopathy; ARSAL [OMIM #611390] (Thiffault et al., 2006; Bayat et al., 2012)	***C. elegans***<br>• RNAi bifunctional *mars-1* knockdown (Zheng et al., 2022a)<br>***Drosophila melanogaster***<br>• *MetRS-m* variant identified in a forward genetic screen causing photoreceptor neuron degeneration (Bayat et al., 2012)<br>**Mouse (*Mus musculus*)**<br>• *Mars2* knockout (Dickinson et al., 2016; Cheong et al., 2020)
NARS2	• Autosomal recessive deafness 94; DFNB94 [OMIM #618434] (Simon et al., 2015; Al-Sharif et al., 2022)<br>• Combined oxidative phosphorylation deficiency 24 [OMIM #616239], Alpers syndrome, Leigh syndrome (Simon et al., 2015; Sofou et al., 2015; Vanlander et al., 2015; Mizuguchi et al., 2017; Seaver et al., 2018; Lee et al., 2020; Li et al., 2021b; Sofou et al., 2021; Štěrbová et al., 2021; Ait-El-Mkadem Saadi et al., 2022; Cokyaman et al., 2022; Hu et al., 2022; Tanaka et al., 2022; Vafaee-Shahi et al., 2022; Yang et al., 2022b; Kistol et al., 2023)	***C. elegans***<br>• RNAi *nars-2* knockdown (Zheng et al., 2022a)<br>**Mouse (*Mus musculus*)**<br>• *Nars2* knockout (Dickinson et al., 2016; Cheong et al., 2020)

	• Developmental delay, epilepsy, and neonatal diabetes (Yagasaki et al., 2022)	
PARS2	• Developmental and epileptic encephalopathy 75; DEE75 [OMIM #618437], Alpers syndrome (Sofou et al., 2012; Sofou et al., 2015; Mizuguchi et al., 2017; Ciara et al., 2018; Yin et al., 2018; Al Balushi et al., 2020; M et al., 2020; Li et al., 2021b; Okamoto et al., 2022)	***C. elegans***<br>• RNAi *pars-2* knockdown (Zheng et al., 2022a)
RARS2	• Fatal infantile encephalopathy with mitochondrial respiratory chain defects (Pontocerebellar Hypoplasia, Type 6 (PCH6)) [OMIM #611523] (Edvardson et al., 2007; Rankin et al., 2010; Namavar et al., 2011; Glamuzina et al., 2012; Cassandrini et al., 2013; Joseph et al., 2014; Li et al., 2015; Alkhateeb et al., 2016; Ngoh et al., 2016; Al Balushi et al., 2020; Nevanlinna et al., 2020; Roux et al., 2021; de Valles-Ibáñez et al., 2022; Nuovo et al., 2022; Zhang et al., 2022)<br>• Epileptic encephalopathy without pontocerebellar hypoplasia (atypical PCH6) (Kastrissianakis et al., 2013; Lühl et al., 2016; Nishri et al., 2016; van Dijk et al., 2017; Mathew et al., 2018; Zhang et al., 2018; Minardi et al., 2020; Xu et al., 2020)<br>• PCH6 with cardiomyopathy, hydrops and pulmonary hypoplasia (Lax et al., 2015)<br>• PCH6 with liver involvement (Sevinç et al., 2022)	***C. elegans***<br>• RNAi *rars-2* knockdown (Zheng et al., 2022a)<br>**Zebrafish (*Danio rerio*)**<br>• Morpholino *rars2* knockdown (Kasher et al., 2011)<br>**Mouse (*Mus musculus*)**<br>• *Rars2* knockout (IMPC data) (Dickinson et al., 2016)
SARS2	• Hyperuricemia, pulmonary hypertension, renal failure, and alkalosis (HUPRA syndrome) [OMIM #613845] (Belostotsky et al., 2011; Rivera et al., 2013; Zhou et al., 2021; Göknar et al., 2022; Yang et al., 2022a)<br>• Progressive spastic paresis (Linnankivi et al., 2016)<br>• HUPRA and progressive spastic paresis with seizures (Yu et al., 2022)<br>• Congenital sideroblastic anemia (Colin et al., 2021)	***C. elegans***<br>• RNAi *sars-2* knockdown (Zheng et al., 2022a)<br>***Drosophila melanogaster***<br>• RNAi *SerRS-m* knockdown (Guitart et al., 2013)<br>**Mouse (*Mus musculus*)**<br>• *Sars2* expression and localization data (Gibbons et al., 2004)<br>• Introduction of human patient variants into *Sars2* (Yu et al., 2022)
TARS2	• Combined oxidative phosphorylation deficiency 21 [OMIM #615918] (Diodato et al., 2014; Li et al., 2020; Gao et al., 2022; Zheng et al., 2022b; He et al., 2023)	***C. elegans***<br>• RNAi bifunctional *tars-1* knockdown (Zheng et al., 2022a)<br>**Mouse (*Mus musculus*)**<br>• *Tars2* knockout (IMPC data) (Dickinson et al., 2016)
VARS2	• Combined oxidative phosphorylation deficiency 20 [OMIM #615917] (Diodato et al., 2014; Taylor et al., 2014; Baertling et al., 2017; Bruni et al., 2018; Ma et al., 2018; Pereira et al., 2018; Begliuomini et al., 2019; Chin et al., 2019; Ruzman et al., 2019; Kušíková et al., 2021; Wu et al., 2022)	***C. elegans***<br>• RNAi *vars-1* knockdown (Zheng et al., 2022a)<br>**Zebrafish (*Danio rerio*)**<br>• Morpholino *vars2* knockdown (Kayvanpour et al., 2022)<br>**Mouse (*Mus musculus*)**

	• Epilepsy, mental retardation, short stature, growth hormone deficiency and hypogonadism (Alsemari et al., 2017)	• *Vars2* knockout (IMPC data) (Dickinson et al., 2016)
WARS2	• Mitochondrial neurodevelopmental disorder, with abnormal movements and lactic acidosis, with or without seizures; NEMMLAS [OMIM #617710] (Theisen et al., 2017; Wortmann et al., 2017; Vantroys et al., 2018; Maffezzini et al., 2019; Virdee et al., 2019; Ilinca et al., 2022)<br>• Childhood-onset Parkinsonism-dystonia 3; PKDYS3 [OMIM #619738] (Burke et al., 2018; Martinelli et al., 2020; Ilinca et al., 2022; Skorvanek et al., 2022)<br>• Hyperkinetic movement disorder (Hübers et al., 2020)<br>• Intellectual disability, ataxia and athetosis (Musante et al., 2017)	***C. elegans***<br>• RNAi *wars-2* knockdown (Zheng et al., 2022a)<br>***Drosophila melanogaster***<br>• RNAi *TrpRS-m* silencing (Maffezzini et al., 2019)<br>**Zebrafish (*Danio rerio*)**<br>• Morpholino *wars2* knockdown (Wang et al., 2016)<br>**Rat (*Rattus norvegicus*)**<br>• Heterozygous and homozygous *Wars2* knockout with zinc finger nuclease (Wang et al., 2016)<br>• *Wars2* variant (p.L53F) identified through linkage analysis with effects on metabolism and coronary blood flow (Wang et al., 2016; Pravenec et al., 2017)<br>**Mouse (*Mus musculus*)**<br>• *Wars2* knockout (IMPC data) (Dickinson et al., 2016)<br>• *Wars2* variant identified in a mutagenesis screen causing age-related hearing loss (Potter et al., 2016)<br>• *Wars2* variant identified in mutagenesis screen causing hearing loss and metabolic changes (Agnew et al., 2018; Mušo et al., 2022)
YARS2	• Myopathy, lactic acidosis, and sideroblastic anemia 2; MLASA2 [OMIM #613561] (Riley et al., 2010; Sasarman et al., 2012; Riley et al., 2013; Shahni et al., 2013; Nakajima et al., 2014; Ardissone et al., 2015; Sommerville et al., 2017; Riley et al., 2018; Smith et al., 2018; Carreño-Gago et al., 2021; Roux et al., 2021; Rudaks et al., 2022)	***C. elegans***<br>• RNAi *yars-2* knockdown (Zheng et al., 2022a)<br>***Drosophila melanogaster***<br>• Interaction between *TyrRS-m* mutation and a mutation in mtDNA-encoded tyrosine tRNA (Meiklejohn et al., 2013; Holmbeck et al., 2015)<br>**Zebrafish (*Danio rerio*)**<br>• *yars2* knockout using CRISPR/Cas9 (Jin et al., 2021)<br>**Dog (*Canis lupus familiaris*)**<br>• Identification of missense *Yars2* variant causing cardiomyopathy and juvenile mortality (Gurtner et al., 2020)

References

Agnew, T., Goldsworthy, M., Aguilar, C., Morgan, A., Simon, M., Hilton, H., et al. (2018). A Wars2 Mutant Mouse Model Displays OXPHOS Deficiencies and Activation of Tissue-Specific Stress Response Pathways. *Cell Rep* 25(12)**,** 3315-3328.e3316. doi: 10.1016/j.celrep.2018.11.080.

Ait-El-Mkadem Saadi, S., Kaphan, E., Morales Jaurrieta, A., Fragaki, K., Chaussenot, A., Bannwarth, S., et al. (2022). Splicing variants in NARS2 are associated with milder phenotypes and intra-familial variability. *Eur J Med Genet* 65(12)**,** 104643. doi: 10.1016/j.ejmg.2022.104643.

Akaike, T., Ida, T., Wei, F.Y., Nishida, M., Kumagai, Y., Alam, M.M., et al. (2017). Cysteinyl-tRNA synthetase governs cysteine polysulfidation and mitochondrial bioenergetics. *Nat Commun* 8(1)**,** 1177. doi: 10.1038/s41467-017-01311-y.

Al Balushi, A., Matviychuk, D., Jobling, R., Salomons, G.S., Blaser, S., and Mercimek-Andrews, S. (2020). Phenotypes and genotypes of mitochondrial aminoacyl-tRNA synthetase deficiencies from a single neurometabolic clinic. *JIMD Rep* 51(1)**,** 3-10. doi: 10.1002/jmd2.12079.

Al-Jaroudi, D., Enabi, S., and AlThagafi, M.S. (2019). Perrault syndrome with amenorrhea, infertility, Tarlov cyst, and degenerative disc. *Gynecol Endocrinol* 35(12)**,** 1037-1039. doi: 10.1080/09513590.2019.1637407.

Al-Sharif, F., Alsadeq, H., Rozan, A., Halabi, M.B., Badwilan, H., Mohammed, A.A., et al. (2022). Bilateral Nonsyndromic Sensorineural Hearing Loss Caused by a NARS2 Mutation. *Cureus* 14(11)**,** e31467. doi: 10.7759/cureus.31467.

Alkhateeb, A.M., Aburahma, S.K., Habbab, W., and Thompson, I.R. (2016). Novel mutations in WWOX, RARS2, and C10orf2 genes in consanguineous Arab families with intellectual disability. *Metab Brain Dis* 31(4)**,** 901-907. doi: 10.1007/s11011-016-9827-9.

Almalki, A., Alston, C.L., Parker, A., Simonic, I., Mehta, S.G., He, L., et al. (2014). Mutation of the human mitochondrial phenylalanine-tRNA synthetase causes infantile-onset epilepsy and cytochrome c oxidase deficiency. *Biochim Biophys Acta* 1842(1)**,** 56-64. doi: 10.1016/j.bbadis.2013.10.008.

Almannai, M., Wang, J., Dai, H., El-Hattab, A.W., Faqeih, E.A., Saleh, M.A., et al. (2018). FARS2 deficiency; new cases, review of clinical, biochemical, and molecular spectra, and variants interpretation based on structural, functional, and evolutionary significance. *Mol Genet Metab* 125(3)**,** 281-291. doi: 10.1016/j.ymgme.2018.07.014.

Alsemari, A., Al-Younes, B., Goljan, E., Jaroudi, D., BinHumaid, F., Meyer, B.F., et al. (2017). Recessive VARS2 mutation underlies a novel syndrome with epilepsy, mental retardation, short stature, growth hormone deficiency, and hypogonadism. *Hum Genomics* 11(1)**,** 28. doi: 10.1186/s40246-017-0124-4.

Amsterdam, A., Nissen, R.M., Sun, Z., Swindell, E.C., Farrington, S., and Hopkins, N. (2004). Identification of 315 genes essential for early zebrafish development. *Proc Natl Acad Sci U S A* 101(35)**,** 12792-12797. doi: 10.1073/pnas.0403929101.

Aradjanski, M., Dogan, S.A., Lotter, S., Wang, S., Hermans, S., Wibom, R., et al. (2017). DARS2 protects against neuroinflammation and apoptotic neuronal loss, but is dispensable for myelin producing cells. *Hum Mol Genet* 26(21)**,** 4181-4189. doi: 10.1093/hmg/ddx307.

Ardissone, A., Lamantea, E., Quartararo, J., Dallabona, C., Carrara, F., Moroni, I., et al. (2015). A Novel Homozygous YARS2 Mutation in Two Italian Siblings and a Review of Literature. *JIMD Rep* 20**,** 95-101. doi: 10.1007/8904_2014_397.

Axelsen, T.M., Vammen, T.L., Bak, M., Pourhadi, N., Stenør, C.M., and Grønborg, S. (2021). Case report: 'AARS2 leukodystrophy'. *Mol Genet Metab Rep* 28**,** 100782. doi: 10.1016/j.ymgmr.2021.100782.

Baertling, F., Alhaddad, B., Seibt, A., Budaeus, S., Meitinger, T., Strom, T.M., et al. (2017). Neonatal encephalocardiomyopathy caused by mutations in VARS2. *Metab Brain Dis* 32(1)**,** 267-270. doi: 10.1007/s11011-016-9890-2.

Barbosa-Gouveia, S., González-Vioque, E., Hermida, Á., Suarez, M.U., Martínez-González, M.J., Borges, F., et al. (2020). Identification of a Novel Variant in EARS2 Associated with a Severe Clinical Phenotype Expands the Clinical Spectrum of LTBL. *Genes (Basel)* 11(9). doi: 10.3390/genes11091028.

Barcia, G., Pandithan, D., Ruzzenente, B., Assouline, Z., Pennisi, A., Ormieres, C., et al. (2021a). Biallelic IARS2 mutations presenting as sideroblastic anemia. *Haematologica* 106(4)**,** 1220-1225. doi: 10.3324/haematol.2020.270710.

Barcia, G., Rio, M., Assouline, Z., Zangarelli, C., Roux, C.J., de Lonlay, P., et al. (2021b). Novel FARS2 variants in patients with early onset encephalopathy with or without epilepsy associated with long survival. *Eur J Hum Genet* 29(3)**,** 533-538. doi: 10.1038/s41431-020-00757-x.

Bayat, V., Thiffault, I., Jaiswal, M., Tetreault, M., Donti, T., Sasarman, F., et al. (2012). Mutations in the mitochondrial methionyl-tRNA synthetase cause a neurodegenerative phenotype in flies and a recessive ataxia (ARSAL) in humans. *PLoS Biol* 10(3)**,** e1001288. doi: 10.1371/journal.pbio.1001288.

Begliuomini, C., Magli, G., Di Rocco, M., Santorelli, F.M., Cassandrini, D., Nesti, C., et al. (2019). VARS2-linked mitochondrial encephalopathy: two case reports enlarging the clinical phenotype. *BMC Med Genet* 20(1)**,** 77. doi: 10.1186/s12881-019-0798-7.

Belostotsky, R., Ben-Shalom, E., Rinat, C., Becker-Cohen, R., Feinstein, S., Zeligson, S., et al. (2011). Mutations in the mitochondrial seryl-tRNA synthetase cause hyperuricemia, pulmonary hypertension, renal failure in infancy and alkalosis, HUPRA syndrome. *Am J Hum Genet* 88(2)**,** 193-200. doi: 10.1016/j.ajhg.2010.12.010.

Biancheri, R., Lamantea, E., Severino, M., Diodato, D., Pedemonte, M., Cassandrini, D., et al. (2015). Expanding the Clinical and Magnetic Resonance Spectrum of Leukoencephalopathy with Thalamus and Brainstem Involvement and High Lactate (LTBL) in a Patient Harboring a Novel EARS2 Mutation. *JIMD Rep* 23**,** 85-89. doi: 10.1007/8904_2015_434.

Bruni, F., Di Meo, I., Bellacchio, E., Webb, B.D., McFarland, R., Chrzanowska-Lightowlers, Z.M.A., et al. (2018). Clinical, biochemical, and genetic features associated with VARS2-related mitochondrial disease. *Hum Mutat* 39(4)**,** 563-578. doi: 10.1002/humu.23398.

Buonfiglio, P.I., Bruque, C.D., Lotersztein, V., Luce, L., Giliberto, F., Menazzi, S., et al. (2022). Predicting pathogenicity for novel hearing loss mutations based on genetic and protein structure approaches. *Sci Rep* 12(1)**,** 301. doi: 10.1038/s41598-021-04081-2.

Burke, E.A., Frucht, S.J., Thompson, K., Wolfe, L.A., Yokoyama, T., Bertoni, M., et al. (2018). Biallelic mutations in mitochondrial tryptophanyl-tRNA synthetase cause Levodopa-responsive infantile-onset Parkinsonism. *Clin Genet* 93(3)**,** 712-718. doi: 10.1111/cge.13172.

Carminho-Rodrigues, M.T., Klee, P., Laurent, S., Guipponi, M., Abramowicz, M., Cao-van, H., et al. (2020). LARS2-Perrault syndrome: a new case report and literature review. *BMC Med Genet* 21(1)**,** 109. doi: 10.1186/s12881-020-01028-8.

Carreño-Gago, L., Juárez-Flores, D.L., Grau, J.M., Ramón, J., Lozano, E., Vila-Julià, F., et al. (2021). Two Novel Variants in YARS2 Gene Are Responsible for an Extended MLASA Phenotype with Pancreatic Insufficiency. *J Clin Med* 10(16). doi: 10.3390/jcm10163471.

Cassandrini, D., Cilio, M.R., Bianchi, M., Doimo, M., Balestri, M., Tessa, A., et al. (2013). Pontocerebellar hypoplasia type 6 caused by mutations in RARS2: definition of the clinical spectrum and molecular findings in five patients. *J Inherit Metab Dis* 36(1)**,** 43-53. doi: 10.1007/s10545-012-9487-9.

Çavuşoğlu, D., Olgaç-Dündar, N., Öztekin, Ö., Özdemir, T.R., Arıcan, P., and Gençpınar, P. (2018). The first pediatric case of leukoencephalopathy with brainstem and spinal cord involvement and lactate elevation (LBSL) from Turkey. *Turk J Pediatr* 60(2)**,** 216-220x. doi: 10.24953/turkjped.2018.02.018.

Chen, X., Liu, F., Li, B., Wang, Y., Yuan, L., Yin, A., et al. (2022). Neuropathy-associated Fars2 deficiency affects neuronal development and potentiates neuronal apoptosis by impairing mitochondrial function. *Cell Biosci* 12(1)**,** 103. doi: 10.1186/s13578-022-00838-y.

Cheng, F.B., Shen, P.P., Zhou, H.W., Meng, H.M., Yang, Y., and Feng, J.C. (2013). Adult-onset leukoencephalopathy with brain stem and spinal cord involvement in Chinese Han population: a case report and literature review. *Neurol India* 61(2)**,** 161-163. doi: 10.4103/0028-3886.111123.

Cheong, A., Archambault, D., Degani, R., Iverson, E., Tremblay, K.D., and Mager, J. (2020). Nuclear-encoded mitochondrial ribosomal proteins are required to initiate gastrulation. *Development* 147(10). doi: 10.1242/dev.188714.

Chin, H.L., Goh, D.L., Wang, F.S., Tay, S.K.H., Heng, C.K., Donnini, C., et al. (2019). A combination of two novel VARS2 variants causes a mitochondrial disorder associated with failure to thrive and pulmonary hypertension. *J Mol Med (Berl)* 97(11)**,** 1557-1566. doi: 10.1007/s00109-019-01834-5.

Ciara, E., Rokicki, D., Lazniewski, M., Mierzewska, H., Jurkiewicz, E., Bekiesińska-Figatowska, M., et al. (2018). Clinical and molecular characteristics of newly reported mitochondrial disease entity caused by biallelic PARS2 mutations. *J Hum Genet* 63(4)**,** 473-485. doi: 10.1038/s10038-017-0401-z.

Cokyaman, T., Cetin, H., Dogan, D., and Silan, F. (2022). A new entity in the NARS2 variant: the first reported case of type 1 diabetes mellitus associated with the phenotype. *J Trop Pediatr* 69(1). doi: 10.1093/tropej/fmac108.

Colin, E., Courtois, G., Brouzes, C., Pulman, J., Rabant, M., Rötig, A., et al. (2021). Biallelic mutations in the SARS2 gene presenting as congenital sideroblastic anemia. *Haematologica* 106(12), 3202-3205. doi: 10.3324/haematol.2021.279138.

Coughlin, C.R., 2nd, Scharer, G.H., Friederich, M.W., Yu, H.C., Geiger, E.A., Creadon-Swindell, G., et al. (2015). Mutations in the mitochondrial cysteinyl-tRNA synthase gene, CARS2, lead to a severe epileptic encephalopathy and complex movement disorder. *J Med Genet* 52(8), 532-540. doi: 10.1136/jmedgenet-2015-103049.

Dallabona, C., Diodato, D., Kevelam, S.H., Haack, T.B., Wong, L.J., Salomons, G.S., et al. (2014). Novel (ovario) leukodystrophy related to AARS2 mutations. *Neurology* 82(23), 2063-2071. doi: 10.1212/WNL.0000000000000497.

Danhauser, K., Haack, T.B., Alhaddad, B., Melcher, M., Seibt, A., Strom, T.M., et al. (2016). EARS2 mutations cause fatal neonatal lactic acidosis, recurrent hypoglycemia and agenesis of corpus callosum. *Metab Brain Dis* 31(3), 717-721. doi: 10.1007/s11011-016-9793-2.

de Valles-Ibáñez, G., Hildebrand, M.S., Bahlo, M., King, C., Coleman, M., Green, T.E., et al. (2022). Infantile-onset myoclonic developmental and epileptic encephalopathy: A new RARS2 phenotype. *Epilepsia Open* 7(1), 170-180. doi: 10.1002/epi4.12553.

Demain, L.A., Urquhart, J.E., O'Sullivan, J., Williams, S.G., Bhaskar, S.S., Jenkinson, E.M., et al. (2017). Expanding the genotypic spectrum of Perrault syndrome. *Clin Genet* 91(2), 302-312. doi: 10.1111/cge.12776.

Demain, L.A.M., Gerkes, E.H., Smith, R.J.H., Molina-Ramirez, L.P., O'Keefe, R.T., and Newman, W.G. (2020). A recurrent missense variant in HARS2 results in variable sensorineural hearing loss in three unrelated families. *J Hum Genet* 65(3), 305-311. doi: 10.1038/s10038-019-0706-1.

Dickinson, M.E., Flenniken, A.M., Ji, X., Teboul, L., Wong, M.D., White, J.K., et al. (2016). High-throughput discovery of novel developmental phenotypes. *Nature* 537(7621), 508-514. doi: 10.1038/nature19356.

Diodato, D., Melchionda, L., Haack, T.B., Dallabona, C., Baruffini, E., Donnini, C., et al. (2014). VARS2 and TARS2 mutations in patients with mitochondrial encephalomyopathies. *Hum Mutat* 35(8), 983-989. doi: 10.1002/humu.22590.

Dogan, S.A., Pujol, C., Maiti, P., Kukat, A., Wang, S., Hermans, S., et al. (2014). Tissue-specific loss of DARS2 activates stress responses independently of respiratory chain deficiency in the heart. *Cell Metab* 19(3), 458-469. doi: 10.1016/j.cmet.2014.02.004.

Edvardson, S., Shaag, A., Kolesnikova, O., Gomori, J.M., Tarassov, I., Einbinder, T., et al. (2007). Deleterious mutation in the mitochondrial arginyl-transfer RNA synthetase gene is associated with pontocerebellar hypoplasia. *Am J Hum Genet* 81(4), 857-862. doi: 10.1086/521227.

Elo, J.M., Yadavalli, S.S., Euro, L., Isohanni, P., Götz, A., Carroll, C.J., et al. (2012). Mitochondrial phenylalanyl-tRNA synthetase mutations underlie fatal infantile Alpers encephalopathy. *Hum Mol Genet* 21(20), 4521-4529. doi: 10.1093/hmg/dds294.

Fan, W., Jin, X., Xu, M., Xi, Y., Lu, W., Yang, X., et al. (2021). FARS2 deficiency in Drosophila reveals the developmental delay and seizure manifested by aberrant mitochondrial tRNA metabolism. *Nucleic Acids Res* 49(22), 13108-13121. doi: 10.1093/nar/gkab1187.

Fan, Y., Han, J., Yang, Y., and Chen, T. (2022). Novel mitochondrial alanyl-tRNA synthetase 2 (AARS2) heterozygous mutations in a Chinese patient with adult-onset leukoencephalopathy. *BMC Neurol* 22(1)**,** 214. doi: 10.1186/s12883-022-02720-3.

Felhi, R., Charif, M., Sfaihi, L., Mkaouar-Rebai, E., Desquiret-Dumas, V., Kallel, R., et al. (2020). Mutations in aARS genes revealed by targeted next-generation sequencing in patients with mitochondrial diseases. *Mol Biol Rep* 47(5)**,** 3779-3787. doi: 10.1007/s11033-020-05425-3.

Forman, E.B., Gorman, K.M., Ennis, S., and King, M.D. (2019). FARS2 Causing Complex Hereditary Spastic Paraplegia With Dysphonia: Expanding the Disease Spectrum. *J Child Neurol* 34(10)**,** 621. doi: 10.1177/0883073819846805.

Gao, X., Xin, G., Tu, Y., Liang, X., Yang, H., Meng, H., et al. (2022). TARS2 variants causes combination oxidative phosphorylation deficiency-21: a case report and literature review. *Neuropediatrics*. doi: 10.1055/a-1949-9310.

Gibbons, W.J., Jr., Yan, Q., Li, R., Li, X., and Guan, M.X. (2004). Genomic organization, expression, and subcellular localization of mouse mitochondrial seryl-tRNA synthetase. *Biochem Biophys Res Commun* 317(3)**,** 774-778. doi: 10.1016/j.bbrc.2004.03.113.

Glamuzina, E., Brown, R., Hogarth, K., Saunders, D., Russell-Eggitt, I., Pitt, M., et al. (2012). Further delineation of pontocerebellar hypoplasia type 6 due to mutations in the gene encoding mitochondrial arginyl-tRNA synthetase, RARS2. *J Inherit Metab Dis* 35(3)**,** 459-467. doi: 10.1007/s10545-011-9413-6.

Göknar, N., Keleşoğlu, E., Kasap, N., Üçkardeş, D., and Candan, C. (2022). A case of chronic kidney disease with pulmonary hypertension, hyperuricemia, immunodeficiency and other extrarenal findings: Answers. *Pediatr Nephrol* 37(11)**,** 2617-2619. doi: 10.1007/s00467-022-05560-y.

Gong, Y., Lan, X.P., and Guo, S. (2022). IARS2-related disease manifesting as sideroblastic anemia and hypoparathyroidism: A case report. *Front Pediatr* 10**,** 1080664. doi: 10.3389/fped.2022.1080664.

Gotz, A., Tyynismaa, H., Euro, L., Ellonen, P., Hyotylainen, T., Ojala, T., et al. (2011). Exome sequencing identifies mitochondrial alanyl-tRNA synthetase mutations in infantile mitochondrial cardiomyopathy. *Am J Hum Genet* 88(5)**,** 635-642. doi: 10.1016/j.ajhg.2011.04.006.

Guerrero, C.M., and Bhatia, S. (2021). FARS2 (Phenylalanyl-tRNA Synthetase 2) Deficiency: A Novel Mutation Associated with EEG Phenotype of Epilepsy of Infancy with Migrating Focal Seizures (EIMFS). *J Pediatr Neurosci* 16(4)**,** 323-326. doi: 10.4103/jpn.JPN_207_20.

Guitart, T., Picchioni, D., Piñeyro, D., and Ribas de Pouplana, L. (2013). Human mitochondrial disease-like symptoms caused by a reduced tRNA aminoacylation activity in flies. *Nucleic Acids Res* 41(13)**,** 6595-6608. doi: 10.1093/nar/gkt402.

Güngör, O., Özkaya, A.K., Şahin, Y., Güngör, G., Dilber, C., and Aydın, K. (2016). A compound heterozygous EARS2 mutation associated with mild leukoencephalopathy with thalamus and brainstem involvement and high lactate (LTBL). *Brain Dev* 38(9)**,** 857-861. doi: 10.1016/j.braindev.2016.04.002.

Gurtner, C., Hug, P., Kleiter, M., Köhler, K., Dietschi, E., Jagannathan, V., et al. (2020). YARS2 Missense Variant in Belgian Shepherd Dogs with Cardiomyopathy and Juvenile Mortality. *Genes (Basel)* 11(3). doi: 10.3390/genes11030313.

Hallmann, K., Zsurka, G., Moskau-Hartmann, S., Kirschner, J., Korinthenberg, R., Ruppert, A.K., et al. (2014). A homozygous splice-site mutation in CARS2 is associated with progressive myoclonic epilepsy. *Neurology* 83(23)**,** 2183-2187. doi: 10.1212/WNL.0000000000001055.

He, P., Wang, Q., Hong, X., and Yuan, H. (2023). Novel TARS2 variant identified in a Chinese patient with mitochondrial encephalomyopathy and a systematic review. *Am J Med Genet A* 191(1)**,** 70-76. doi: 10.1002/ajmg.a.62988.

Hilander, T., Zhou, X.L., Konovalova, S., Zhang, F.P., Euro, L., Chilov, D., et al. (2018). Editing activity for eliminating mischarged tRNAs is essential in mammalian mitochondria. *Nucleic Acids Res* 46(2)**,** 849-860. doi: 10.1093/nar/gkx1231.

Holmbeck, M.A., Donner, J.R., Villa-Cuesta, E., and Rand, D.M. (2015). A Drosophila model for mito-nuclear diseases generated by an incompatible interaction between tRNA and tRNA synthetase. *Dis Model Mech* 8(8)**,** 843-854. doi: 10.1242/dmm.019323.

Hotait, M., Nasreddine, W., El-Khoury, R., Dirani, M., Nawfal, O., and Beydoun, A. (2020). FARS2 Mutations: More Than Two Phenotypes? A Case Report. *Front Genet* 11**,** 787. doi: 10.3389/fgene.2020.00787.

Hu, W., Fang, H., Peng, Y., Li, L., Guo, D., Tang, J., et al. (2022). Clinical and genetic analyses of premature mitochondrial encephalopathy with epilepsia partialis continua caused by novel biallelic NARS2 mutations. *Front Neurosci* 16**,** 1076183. doi: 10.3389/fnins.2022.1076183.

Hübers, A., Huppertz, H.J., Wortmann, S.B., and Kassubek, J. (2020). Mutation of the WARS2 Gene as the Cause of a Severe Hyperkinetic Movement Disorder. *Mov Disord Clin Pract* 7(1)**,** 88-90. doi: 10.1002/mdc3.12855.

Ilinca, A., Kafantari, E., and Puschmann, A. (2022). A relatively common hypomorphic variant in WARS2 causes monogenic disease. *Parkinsonism Relat Disord* 94**,** 129-131. doi: 10.1016/j.parkreldis.2022.01.012.

Isohanni, P., Linnankivi, T., Buzkova, J., Lönnqvist, T., Pihko, H., Valanne, L., et al. (2010). DARS2 mutations in mitochondrial leucoencephalopathy and multiple sclerosis. *J Med Genet* 47(1)**,** 66-70. doi: 10.1136/jmg.2009.068221.

Jabbour, S., and Harissi-Dagher, M. (2016). Recessive Mutation in a Nuclear-Encoded Mitochondrial tRNA Synthetase Associated With Infantile Cataract, Congenital Neurotrophic Keratitis, and Orbital Myopathy. *Cornea* 35(6)**,** 894-896. doi: 10.1097/ico.0000000000000847.

Jin, X., Zhang, Z., Nie, Z., Wang, C., Meng, F., Yi, Q., et al. (2021). An animal model for mitochondrial tyrosyl-tRNA synthetase deficiency reveals links between oxidative phosphorylation and retinal function. *J Biol Chem* 296**,** 100437. doi: 10.1016/j.jbc.2021.100437.

Joseph, J.T., Innes, A.M., Smith, A.C., Vanstone, M.R., Schwartzentruber, J.A., Bulman, D.E., et al. (2014). Neuropathologic features of pontocerebellar hypoplasia type 6. *J Neuropathol Exp Neurol* 73(11)**,** 1009-1025. doi: 10.1097/nen.0000000000000123.

Kapoor, D., Majethia, P., Anand, A., Shukla, A., and Sharma, S. (2021). Expanding the electro-clinical phenotype of CARS2associated neuroregression. *Epilepsy Behav Rep* 16**,** 100485. doi: 10.1016/j.ebr.2021.100485.

Kasher, P.R., Namavar, Y., van Tijn, P., Fluiter, K., Sizarov, A., Kamermans, M., et al. (2011). Impairment of the tRNA-splicing endonuclease subunit 54 (tsen54) gene causes neurological abnormalities and larval death in zebrafish models of pontocerebellar hypoplasia. *Hum Mol Genet* 20(8)**,** 1574-1584. doi: 10.1093/hmg/ddr034.

Kastrissianakis, K., Anand, G., Quaghebeur, G., Price, S., Prabhakar, P., Marinova, J., et al. (2013). Subdural effusions and lack of early pontocerebellar hypoplasia in siblings with RARS2 mutations. *Arch Dis Child* 98(12)**,** 1004-1007. doi: 10.1136/archdischild-2013-304308.

Kayvanpour, E., Wisdom, M., Lackner, M.K., Sedaghat-Hamedani, F., Boeckel, J.N., Müller, M., et al. (2022). VARS2 Depletion Leads to Activation of the Integrated Stress Response and Disruptions in Mitochondrial Fatty Acid Oxidation. *Int J Mol Sci* 23(13). doi: 10.3390/ijms23137327.

Kevelam, S.H., Klouwer, F.C., Fock, J.M., Salomons, G.S., Bugiani, M., and van der Knaap, M.S. (2016). Absent Thalami Caused by a Homozygous EARS2 Mutation: Expanding Disease Spectrum of LTBL. *Neuropediatrics* 47(1)**,** 64-67. doi: 10.1055/s-0035-1568987.

Kiraly-Borri, C., Jevon, G., Ji, W., Jeffries, L., Ricciardi, J.L., Konstantino, M., et al. (2019). Siblings with lethal primary pulmonary hypoplasia and compound heterozygous variants in the AARS2 gene: further delineation of the phenotypic spectrum. *Cold Spring Harb Mol Case Stud* 5(3). doi: 10.1101/mcs.a003699.

Kistol, D., Tsygankova, P., Krylova, T., Bychkov, I., Itkis, Y., Nikolaeva, E., et al. (2023). Leigh Syndrome: Spectrum of Molecular Defects and Clinical Features in Russia. *Int J Mol Sci* 24(2). doi: 10.3390/ijms24021597.

Köhler, C., Heyer, C., Hoffjan, S., Stemmler, S., Lücke, T., Thiels, C., et al. (2015). Early-onset leukoencephalopathy due to a homozygous missense mutation in the DARS2 gene. *Mol Cell Probes* 29(5)**,** 319-322. doi: 10.1016/j.mcp.2015.06.005.

Kosaki, R., Horikawa, R., Fujii, E., and Kosaki, K. (2018). Biallelic mutations in LARS2 can cause Perrault syndrome type 2 with neurologic symptoms. *Am J Med Genet A* 176(2)**,** 404-408. doi: 10.1002/ajmg.a.38552.

Kuo, M.E., Antonellis, A., and Shakkottai, V.G. (2020). Alanyl-tRNA Synthetase 2 (AARS2)-Related Ataxia Without Leukoencephalopathy. *Cerebellum* 19(1)**,** 154-160. doi: 10.1007/s12311-019-01080-y.

Kušíková, K., Feichtinger, R.G., Csillag, B., Kalev, O.K., Weis, S., Duba, H.C., et al. (2021). Case Report and Review of the Literature: A New and a Recurrent Variant in the VARS2 Gene Are Associated With Isolated Lethal Hypertrophic Cardiomyopathy, Hyperlactatemia, and Pulmonary Hypertension in Early Infancy. *Front Pediatr* 9**,** 660076. doi: 10.3389/fped.2021.660076.

Labauge, P., Dorboz, I., Eymard-Pierre, E., Dereeper, O., and Boespflug-Tanguy, O. (2011). Clinically asymptomatic adult patient with extensive LBSL MRI pattern and DARS2 mutations. *J Neurol* 258(2)**,** 335-337. doi: 10.1007/s00415-010-5755-5.

Lan, M.Y., Chang, Y.Y., Yeh, T.H., Lin, T.K., and Lu, C.S. (2017). Leukoencephalopathy with brainstem and spinal cord involvement and lactate elevation (LBSL) with a novel DARS2 mutation and isolated progressive spastic paraparesis. *J Neurol Sci* 372**,** 229-231. doi: 10.1016/j.jns.2016.11.058.

Lax, N.Z., Alston, C.L., Schon, K., Park, S.M., Krishnakumar, D., He, L., et al. (2015). Neuropathologic Characterization of Pontocerebellar Hypoplasia Type 6 Associated With Cardiomyopathy and Hydrops Fetalis and Severe Multisystem Respiratory Chain Deficiency due to Novel RARS2 Mutations. *J Neuropathol Exp Neurol* 74(7)**,** 688-703. doi: 10.1097/nen.0000000000000209.

Lee, J.S., Yoo, T., Lee, M., Lee, Y., Jeon, E., Kim, S.Y., et al. (2020). Genetic heterogeneity in Leigh syndrome: Highlighting treatable and novel genetic causes. *Clin Genet* 97(4)**,** 586-594. doi: 10.1111/cge.13713.

Lee, S.S., Lee, R.Y., Fraser, A.G., Kamath, R.S., Ahringer, J., and Ruvkun, G. (2003). A systematic RNAi screen identifies a critical role for mitochondria in C. elegans longevity. *Nat Genet* 33(1)**,** 40-48. doi: 10.1038/ng1056.

Lerat, J., Jonard, L., Loundon, N., Christin-Maitre, S., Lacombe, D., Goizet, C., et al. (2016). An Application of NGS for Molecular Investigations in Perrault Syndrome: Study of 14 Families and Review of the Literature. *Hum Mutat* 37(12)**,** 1354-1362. doi: 10.1002/humu.23120.

Li, B., Chen, K., Liu, F., Zhang, J., Chen, X., Chen, T., et al. (2021a). Developmental Angiogenesis Requires the Mitochondrial Phenylalanyl-tRNA Synthetase. *Front Cardiovasc Med* 8**,** 724846. doi: 10.3389/fcvm.2021.724846.

Li, H., Wang, W., Han, X., Zhang, Y., Dai, L., Xu, M., et al. (2021b). Clinical Attributes and Electroencephalogram Analysis of Patients With Varying Alpers' Syndrome Genotypes. *Front Pharmacol* 12**,** 669516. doi: 10.3389/fphar.2021.669516.

Li, J.L., Lee, N.C., Chen, P.S., Lee, G.H., and Wu, R.M. (2021c). Leukoencephalopathy with Brainstem and Spinal Cord Involvement and Lactate Elevation: A Novel DARS2 Mutation and Intra-Familial Heterogeneity. *Mov Disord Clin Pract* 8(7)**,** 1116-1122. doi: 10.1002/mdc3.13281.

Li, X., Peng, B., Hou, C., Li, J., Zeng, Y., Wu, W., et al. (2020). Novel compound heterozygous TARS2 variants in a Chinese family with mitochondrial encephalomyopathy: a case report. *BMC Med Genet* 21(1)**,** 217. doi: 10.1186/s12881-020-01149-0.

Li, Z., Schonberg, R., Guidugli, L., Johnson, A.K., Arnovitz, S., Yang, S., et al. (2015). A novel mutation in the promoter of RARS2 causes pontocerebellar hypoplasia in two siblings. *J Hum Genet* 60(7)**,** 363-369. doi: 10.1038/jhg.2015.31.

Liberfarb, R.M., Jackson, A.H., Eavey, R.D., and Robb, R.M. (1993). Unique hereditary sensory and autonomic neuropathy with growth hormone deficiency. *J Child Neurol* 8(3)**,** 271-276. doi: 10.1177/088307389300800312.

Lin, J., Chiconelli Faria, E., Da Rocha, A.J., Rodrigues Masruha, M., Pereira Vilanova, L.C., Scheper, G.C., et al. (2010). Leukoencephalopathy with brainstem and spinal cord involvement and normal lactate: a new mutation in the DARS2 gene. *J Child Neurol* 25(11)**,** 1425-1428. doi: 10.1177/0883073810370897.

Lin, T.K., Chang, Y.Y., Lin, H.Y., Liou, C.W., Wang, P.W., Chuang, J.H., et al. (2019). Mitochondrial dysfunctions in leukoencephalopathy with brainstem and spinal cord involvement and lactate elevation (LBSL). *PLoS One* 14(10)**,** e0224173. doi: 10.1371/journal.pone.0224173.

Linnankivi, T., Neupane, N., Richter, U., Isohanni, P., and Tyynismaa, H. (2016). Splicing Defect in Mitochondrial Seryl-tRNA Synthetase Gene Causes Progressive Spastic Paresis Instead of HUPRA Syndrome. *Hum Mutat* 37(9)**,** 884-888. doi: 10.1002/humu.23021.

Lühl, S., Bode, H., Schlötzer, W., Bartsakoulia, M., Horvath, R., Abicht, A., et al. (2016). Novel homozygous RARS2 mutation in two siblings without pontocerebellar hypoplasia - further expansion of the phenotypic spectrum. *Orphanet J Rare Dis* 11(1)**,** 140. doi: 10.1186/s13023-016-0525-9.

Lynch, D.S., Zhang, W.J., Lakshmanan, R., Kinsella, J.A., Uzun, G.A., Karbay, M., et al. (2016). Analysis of Mutations in AARS2 in a Series of CSF1R-Negative Patients With Adult-Onset Leukoencephalopathy With Axonal Spheroids and Pigmented Glia. *JAMA Neurol* 73(12)**,** 1433-1439. doi: 10.1001/jamaneurol.2016.2229.

M, A.A., Vernon, H.J., Ferguson, M., and Kline, A.D. (2020). PARS2-associated mitochondrial disease: A case report of a patient with prolonged survival and literature review. *Mol Genet Metab Rep* 24**,** 100613. doi: 10.1016/j.ymgmr.2020.100613.

Ma, K., Xie, M., He, X., Liu, G., Lu, X., Peng, Q., et al. (2018). A novel compound heterozygous mutation in VARS2 in a newborn with mitochondrial cardiomyopathy: a case report of a Chinese family. *BMC Med Genet* 19(1)**,** 202. doi: 10.1186/s12881-018-0689-3.

Maffezzini, C., Laine, I., Dallabona, C., Clemente, P., Calvo-Garrido, J., Wibom, R., et al. (2019). Mutations in the mitochondrial tryptophanyl-tRNA synthetase cause growth retardation and progressive leukoencephalopathy. *Mol Genet Genomic Med* 7(6)**,** e654. doi: 10.1002/mgg3.654.

Martikainen, M.H., Ellfolk, U., and Majamaa, K. (2013). Impaired information-processing speed and working memory in leukoencephalopathy with brainstem and spinal cord involvement and elevated lactate (LBSL) and DARS2 mutations: a report of three adult patients. *J Neurol* 260(8)**,** 2078-2083. doi: 10.1007/s00415-013-6940-0.

Martinelli, S., Cordeddu, V., Galosi, S., Lanzo, A., Palma, E., Pannone, L., et al. (2020). Co-occurring WARS2 and CHRNA6 mutations in a child with a severe form of infantile parkinsonism. *Parkinsonism Relat Disord* 72**,** 75-79. doi: 10.1016/j.parkreldis.2020.02.003.

Mathew, T., Avati, A., D'Souza, D., and Therambil, M. (2018). Expanding spectrum of RARS2 gene disorders: Myoclonic epilepsy, mental retardation, spasticity, and extrapyramidal features. *Epilepsia Open* 3(2)**,** 270-275. doi: 10.1002/epi4.12108.

Mazurova, S., Magner, M., Kucerova-Vidrova, V., Vondrackova, A., Stranecky, V., Pristoupilova, A., et al. (2017). Thymidine kinase 2 and alanyl-tRNA synthetase 2 deficiencies cause lethal mitochondrial cardiomyopathy: case reports and review of the literature. *Cardiol Young* 27(5)**,** 936-944. doi: 10.1017/s1047951116001876.

Meiklejohn, C.D., Holmbeck, M.A., Siddiq, M.A., Abt, D.N., Rand, D.M., and Montooth, K.L. (2013). An Incompatibility between a mitochondrial tRNA and its nuclear-encoded tRNA synthetase compromises development and fitness in Drosophila. *PLoS Genet* 9(1)**,** e1003238. doi: 10.1371/journal.pgen.1003238.

Meszarosova, A.U., Seeman, P., Jencik, J., Drabova, J., Cibochova, R., Stellmachova, J., et al. (2020). Two types of recessive hereditary spastic paraplegia in Roma patients in compound heterozygous state; no ethnically prevalent variant found. *Neurosci Lett* 721**,** 134800. doi: 10.1016/j.neulet.2020.134800.

Mierzewska, H., van der Knaap, M.S., Scheper, G.C., Bekiesinska-Figatowska, M., Szczepanik, E., and Jurkiewicz, E. (2011). Leukoencephalopathy with brain stem and spinal cord involvement and lactate elevation in the first Polish patient. *Brain Dev* 33(9)**,** 713-717. doi: 10.1016/j.braindev.2010.12.005.

Minardi, R., Licchetta, L., Baroni, M.C., Pippucci, T., Stipa, C., Mostacci, B., et al. (2020). Whole-exome sequencing in adult patients with developmental and epileptic encephalopathy: It is never too late. *Clin Genet* 98(5)**,** 477-485. doi: 10.1111/cge.13823.

Miyake, N., Yamashita, S., Kurosawa, K., Miyatake, S., Tsurusaki, Y., Doi, H., et al. (2011). A novel homozygous mutation of DARS2 may cause a severe LBSL variant. *Clin Genet* 80(3)**,** 293-296. doi: 10.1111/j.1399-0004.2011.01644.x.

Mizuguchi, T., Nakashima, M., Kato, M., Yamada, K., Okanishi, T., Ekhilevitch, N., et al. (2017). PARS2 and NARS2 mutations in infantile-onset neurodegenerative disorder. *J Hum Genet* 62(5)**,** 525-529. doi: 10.1038/jhg.2016.163.

Mo, L., Li, R., He, C., Chen, Q., Xu, C., Shen, L., et al. (2023). Hedgehog pathway is negatively regulated during the development of Drosophila melanogaster PheRS-m (Drosophila homologs gene of human FARS2) mutants. *Hum Cell* 36(1)**,** 121-131. doi: 10.1007/s13577-022-00796-0.

Moosa, S., Haagerup, A., Gregersen, P.A., Petersen, K.K., Altmüller, J., Thiele, H., et al. (2017). Confirmation of CAGSSS syndrome as a distinct entity in a Danish patient with a novel homozygous mutation in IARS2. *Am J Med Genet A* 173(4)**,** 1102-1108. doi: 10.1002/ajmg.a.38116.

Musante, L., Püttmann, L., Kahrizi, K., Garshasbi, M., Hu, H., Stehr, H., et al. (2017). Mutations of the aminoacyl-tRNA-synthetases SARS and WARS2 are implicated in the etiology of autosomal recessive intellectual disability. *Hum Mutat* 38(6)**,** 621-636. doi: 10.1002/humu.23205.

Mušo, M., Bentley, L., Vizor, L., Yon, M., Burling, K., Barker, P., et al. (2022). A Wars2 mutant mouse shows a sex and diet specific change in fat distribution, reduced food intake and depot-specific upregulation of WAT browning. *Front Physiol* 13**,** 953199. doi: 10.3389/fphys.2022.953199.

Nakajima, J., Eminoglu, T.F., Vatansever, G., Nakashima, M., Tsurusaki, Y., Saitsu, H., et al. (2014). A novel homozygous YARS2 mutation causes severe myopathy, lactic acidosis, and sideroblastic anemia 2. *J Hum Genet* 59(4)**,** 229-232. doi: 10.1038/jhg.2013.143.

Nakayama, T., Wu, J., Galvin-Parton, P., Weiss, J., Andriola, M.R., Hill, R.S., et al. (2017). Deficient activity of alanyl-tRNA synthetase underlies an autosomal recessive syndrome of progressive microcephaly, hypomyelination, and epileptic encephalopathy. *Hum Mutat* 38(10)**,** 1348-1354. doi: 10.1002/humu.23250.

Namavar, Y., Barth, P.G., Kasher, P.R., van Ruissen, F., Brockmann, K., Bernert, G., et al. (2011). Clinical, neuroradiological and genetic findings in pontocerebellar hypoplasia. *Brain* 134(Pt 1)**,** 143-156. doi: 10.1093/brain/awq287.

Nemeth, C.L., Tomlinson, S.N., Rosen, M., O'Brien, B.M., Larraza, O., Jain, M., et al. (2020). Neuronal ablation of mt-AspRS in mice induces immune pathway activation prior to severe and progressive cortical and behavioral disruption. *Exp Neurol* 326**,** 113164. doi: 10.1016/j.expneurol.2019.113164.

Nevanlinna, V., Konovalova, S., Ceulemans, B., Muona, M., Laari, A., Hilander, T., et al. (2020). A patient with pontocerebellar hypoplasia type 6: Novel RARS2 mutations, comparison to previously published patients and clinical distinction from PEHO syndrome. *Eur J Med Genet* 63(3)**,** 103766. doi: 10.1016/j.ejmg.2019.103766.

Neyroud, A.S., Rudinger-Thirion, J., Frugier, M., Riley, L.G., Bidet, M., Akloul, L., et al. (2022). LARS2 variants can present as premature ovarian insufficiency in the absence of overt hearing loss. *Eur J Hum Genet*. doi: 10.1038/s41431-022-01252-1.

Ngo, J., Prokop, J.W., Umfleet, J., and Seaver, L.H. (2021). Perinatal Manifestations of DARS2-Associated Leukoencephalopathy With Brainstem and Spinal Cord Involvement and Lactate Elevation (LBSL). *Child Neurol Open* 8**,** 2329048x211019173. doi: 10.1177/2329048x211019173.

Ngoh, A., Bras, J., Guerreiro, R., Meyer, E., McTague, A., Dawson, E., et al. (2016). RARS2 mutations in a sibship with infantile spasms. *Epilepsia* 57(5)**,** e97-e102. doi: 10.1111/epi.13358.

Ni, M., Black, L.F., Pan, C., Vu, H., Pei, J., Ko, B., et al. (2021). Metabolic impact of pathogenic variants in the mitochondrial glutamyl-tRNA synthetase EARS2. *J Inherit Metab Dis* 44(4)**,** 949-960. doi: 10.1002/jimd.12387.

Nielsen, S.K., Hansen, F., Schroder, H.D., Wibrand, F., Gustafsson, F., and Mogensen, J. (2020). Recessive Inheritance of a Rare Variant in the Nuclear Mitochondrial Gene for AARS2 in Late-Onset Dilated Cardiomyopathy. *Circ Genom Precis Med* 13(5)**,** 560-562. doi: 10.1161/CIRCGEN.120.003086.

Nishri, D., Goldberg-Stern, H., Noyman, I., Blumkin, L., Kivity, S., Saitsu, H., et al. (2016). RARS2 mutations cause early onset epileptic encephalopathy without ponto-cerebellar hypoplasia. *Eur J Paediatr Neurol* 20(3)**,** 412-417. doi: 10.1016/j.ejpn.2016.02.012.

Nuovo, S., Micalizzi, A., Romaniello, R., Arrigoni, F., Ginevrino, M., Casella, A., et al. (2022). Refining the mutational spectrum and gene-phenotype correlates in pontocerebellar hypoplasia: results of a multicentric study. *J Med Genet* 59(4)**,** 399-409. doi: 10.1136/jmedgenet-2020-107497.

Okamoto, N., Miya, F., Tsunoda, T., Kanemura, Y., Saitoh, S., Kato, M., et al. (2022). Four pedigrees with aminoacyl-tRNA synthetase abnormalities. *Neurol Sci* 43(4)**,** 2765-2774. doi: 10.1007/s10072-021-05626-z.

Oliveira, R., Sommerville, E.W., Thompson, K., Nunes, J., Pyle, A., Grazina, M., et al. (2017). Lethal Neonatal LTBL Associated with Biallelic EARS2 Variants: Case Report and Review of the Reported Neuroradiological Features. *JIMD Rep* 33**,** 61-68. doi: 10.1007/8904_2016_581.

Orcesi, S., La Piana, R., Uggetti, C., Tonduti, D., Pichiecchio, A., Pasin, M., et al. (2011). Spinal cord calcification in an early-onset progressive leukoencephalopathy. *J Child Neurol* 26(7)**,** 876-880. doi: 10.1177/0883073810390038.

Pan, Z., Xu, H., Tian, Y., Liu, D., Liu, H., Li, R., et al. (2020). Perrault syndrome: Clinical report and retrospective analysis. *Mol Genet Genomic Med* 8(10)**,** e1445. doi: 10.1002/mgg3.1445.

Peragallo, J.H., Keller, S., van der Knaap, M.S., Soares, B.P., and Shankar, S.P. (2018). Retinopathy and optic atrophy: Expanding the phenotypic spectrum of pathogenic variants in the AARS2 gene. *Ophthalmic Genet* 39(1)**,** 99-102. doi: 10.1080/13816810.2017.1350723.

Pereira, S., Adrião, M., Sampaio, M., Basto, M.A., Rodrigues, E., Vilarinho, L., et al. (2018). Mitochondrial Encephalopathy: First Portuguese Report of a VARS2 Causative Variant. *JIMD Rep* 42**,** 113-119. doi: 10.1007/8904_2018_89.

Petzold, G.C., Bohner, G., Klingebiel, R., Amberger, N., van der Knaap, M.S., and Zschenderlein, R. (2006). Adult onset leucoencephalopathy with brain stem and spinal cord involvement and normal lactate. *J Neurol Neurosurg Psychiatry* 77(7)**,** 889-891. doi: 10.1136/jnnp.2005.078568.

Pierce, S.B., Chisholm, K.M., Lynch, E.D., Lee, M.K., Walsh, T., Opitz, J.M., et al. (2011). Mutations in mitochondrial histidyl tRNA synthetase HARS2 cause ovarian dysgenesis and sensorineural hearing loss of Perrault syndrome. *Proc Natl Acad Sci U S A* 108(16)**,** 6543-6548. doi: 10.1073/pnas.1103471108.

Pierce, S.B., Gersak, K., Michaelson-Cohen, R., Walsh, T., Lee, M.K., Malach, D., et al. (2013). Mutations in LARS2, encoding mitochondrial leucyl-tRNA synthetase, lead to premature ovarian failure and hearing loss in Perrault syndrome. *Am J Hum Genet* 92(4)**,** 614-620. doi: 10.1016/j.ajhg.2013.03.007.

Potter, P.K., Bowl, M.R., Jeyarajan, P., Wisby, L., Blease, A., Goldsworthy, M.E., et al. (2016). Novel gene function revealed by mouse mutagenesis screens for models of age-related disease. *Nat Commun* 7**,** 12444. doi: 10.1038/ncomms12444.

Pravenec, M., Zídek, V., Landa, V., Mlejnek, P., Šilhavý, J., Šimáková, M., et al. (2017). Mutant Wars2 gene in spontaneously hypertensive rats impairs brown adipose tissue function and predisposes to visceral obesity. *Physiol Res* 66(6)**,** 917-924. doi: 10.33549/physiolres.933811.

Rankin, J., Brown, R., Dobyns, W.B., Harington, J., Patel, J., Quinn, M., et al. (2010). Pontocerebellar hypoplasia type 6: A British case with PEHO-like features. *Am J Med Genet A* 152a(8)**,** 2079-2084. doi: 10.1002/ajmg.a.33531.

Raviglione, F., Conte, G., Ghezzi, D., Parazzini, C., Righini, A., Vergaro, R., et al. (2016). Clinical findings in a patient with FARS2 mutations and early-infantile-encephalopathy with epilepsy. *Am J Med Genet A* 170(11)**,** 3004-3007. doi: 10.1002/ajmg.a.37836.

Riley, L.G., Cooper, S., Hickey, P., Rudinger-Thirion, J., McKenzie, M., Compton, A., et al. (2010). Mutation of the mitochondrial tyrosyl-tRNA synthetase gene, YARS2, causes myopathy, lactic acidosis, and sideroblastic anemia--MLASA syndrome. *Am J Hum Genet* 87(1)**,** 52-59. doi: 10.1016/j.ajhg.2010.06.001.

Riley, L.G., Heeney, M.M., Rudinger-Thirion, J., Frugier, M., Campagna, D.R., Zhou, R., et al. (2018). The phenotypic spectrum of germline YARS2 variants: from isolated sideroblastic anemia to mitochondrial myopathy, lactic acidosis and sideroblastic anemia 2. *Haematologica* 103(12)**,** 2008-2015. doi: 10.3324/haematol.2017.182659.

Riley, L.G., Menezes, M.J., Rudinger-Thirion, J., Duff, R., de Lonlay, P., Rotig, A., et al. (2013). Phenotypic variability and identification of novel YARS2 mutations in YARS2 mitochondrial myopathy, lactic acidosis and sideroblastic anaemia. *Orphanet J Rare Dis* 8**,** 193. doi: 10.1186/1750-1172-8-193.

Riley, L.G., Rudinger-Thirion, J., Frugier, M., Wilson, M., Luig, M., Alahakoon, T.I., et al. (2020). The expanding LARS2 phenotypic spectrum: HLASA, Perrault syndrome with leukodystrophy, and mitochondrial myopathy. *Hum Mutat* 41(8)**,** 1425-1434. doi: 10.1002/humu.24050.

Riley, L.G., Rudinger-Thirion, J., Schmitz-Abe, K., Thorburn, D.R., Davis, R.L., Teo, J., et al. (2016). LARS2 Variants Associated with Hydrops, Lactic Acidosis, Sideroblastic Anemia, and Multisystem Failure. *JIMD Rep* 28**,** 49-57. doi: 10.1007/8904_2015_515.

Rivera, H., Martín-Hernández, E., Delmiro, A., García-Silva, M.T., Quijada-Fraile, P., Muley, R., et al. (2013). A new mutation in the gene encoding mitochondrial seryl-tRNA synthetase as a cause of HUPRA syndrome. *BMC Nephrol* 14**,** 195. doi: 10.1186/1471-2369-14-195.

Roux, C.J., Barcia, G., Schiff, M., Sissler, M., Levy, R., Dangouloff-Ros, V., et al. (2021). Phenotypic diversity of brain MRI patterns in mitochondrial aminoacyl-tRNA synthetase mutations. *Mol Genet Metab* 133(2)**,** 222-229. doi: 10.1016/j.ymgme.2021.04.004.

Rudaks, L.I., Watson, E., Oboudiyat, C., Kumar, K.R., Sullivan, P., Cowley, M.J., et al. (2022). Decompensation of cardiorespiratory function and emergence of anemia during pregnancy in a case of mitochondrial myopathy, lactic acidosis, and sideroblastic anemia 2 with compound heterozygous YARS2 pathogenic variants. *Am J Med Genet A* 188(7)**,** 2226-2230. doi: 10.1002/ajmg.a.62755.

Rumyantseva, A., Motori, E., and Trifunovic, A. (2020). DARS2 is indispensable for Purkinje cell survival and protects against cerebellar ataxia. *Hum Mol Genet* 29(17)**,** 2845-2854. doi: 10.1093/hmg/ddaa176.

Ruzman, L., Kolic, I., Radic Nisevic, J., Ruzic Barsic, A., Skarpa Prpic, I., and Prpic, I. (2019). A novel VARS2 gene variant in a patient with epileptic encephalopathy. *Ups J Med Sci* 124(4)**,** 273-277. doi: 10.1080/03009734.2019.1670297.

Şahin, S., Cansu, A., Kalay, E., Dinçer, T., Kul, S., Çakır İ, M., et al. (2016). Leukoencephalopathy with thalamus and brainstem involvement and high lactate caused by novel mutations in the EARS2 gene in two siblings. *J Neurol Sci* 365**,** 54-58. doi: 10.1016/j.jns.2016.04.008.

Samanta, D., Gokden, M., and Willis, E. (2018). Clinicopathologic Findings of CARS2 Mutation. *Pediatr Neurol* 87**,** 65-69. doi: 10.1016/j.pediatrneurol.2018.06.009.

Sasarman, F., Nishimura, T., Thiffault, I., and Shoubridge, E.A. (2012). A novel mutation in YARS2 causes myopathy with lactic acidosis and sideroblastic anemia. *Hum Mutat* 33(8)**,** 1201-1206. doi: 10.1002/humu.22098.

Sawada, D., Naito, S., Aoyama, H., Shiohama, T., Ichikawa, T., Imagawa, E., et al. (2021). Remitting and exacerbating white matter lesions in leukoencephalopathy with thalamus and brainstem involvement and high lactate. *Brain Dev* 43(7)**,** 798-803. doi: 10.1016/j.braindev.2021.03.008.

Scheper, G.C., van der Klok, T., van Andel, R.J., van Berkel, C.G., Sissler, M., Smet, J., et al. (2007). Mitochondrial aspartyl-tRNA synthetase deficiency causes leukoencephalopathy with brain stem and spinal cord involvement and lactate elevation. *Nat Genet* 39(4)**,** 534-539. doi: 10.1038/ng2013.

Schwartzentruber, J., Buhas, D., Majewski, J., Sasarman, F., Papillon-Cavanagh, S., Thiffault, I., et al. (2014). Mutation in the nuclear-encoded mitochondrial isoleucyl-tRNA synthetase IARS2 in patients with cataracts, growth hormone deficiency with short stature, partial sensorineural deafness, and peripheral neuropathy or with Leigh syndrome. *Hum Mutat* 35(11)**,** 1285-1289. doi: 10.1002/humu.22629.

Seaver, L.H., DeRoos, S., Andersen, N.J., Betz, B., Prokop, J., Lannen, N., et al. (2018). Lethal NARS2-Related Disorder Associated With Rapidly Progressive Intractable Epilepsy and Global Brain Atrophy. *Pediatr Neurol* 89**,** 26-30. doi: 10.1016/j.pediatrneurol.2018.07.014.

Sellars, E.A., Balmakund, T., Bosanko, K., Nichols, B.L., Kahler, S.G., and Zarate, Y.A. (2017). Severe Metabolic Acidosis and Hepatopathy due to Leukoencephalopathy with Thalamus and Brainstem Involvement and High Lactate. *Neuropediatrics* 48(2)**,** 108-110. doi: 10.1055/s-0036-1593984.

Sevinç, S., İnci, A., Ezgü, F.S., and Eminoğlu, F.T. (2022). A Patient with a Novel RARS2 Variant Exhibiting Liver Involvement as a New Clinical Feature and Review of the Literature. *Mol Syndromol* 13(3)**,** 226-234. doi: 10.1159/000519604.

Shahni, R., Wedatilake, Y., Cleary, M.A., Lindley, K.J., Sibson, K.R., and Rahman, S. (2013). A distinct mitochondrial myopathy, lactic acidosis and sideroblastic anemia (MLASA) phenotype associates with YARS2 mutations. *Am J Med Genet A* 161a(9)**,** 2334-2338. doi: 10.1002/ajmg.a.36065.

Shamseldin, H.E., Alshammari, M., Al-Sheddi, T., Salih, M.A., Alkhalidi, H., Kentab, A., et al. (2012). Genomic analysis of mitochondrial diseases in a consanguineous population reveals novel candidate disease genes. *J Med Genet* 49(4)**,** 234-241. doi: 10.1136/jmedgenet-2012-100836.

Sharma, S., Sankhyan, N., Kumar, A., Scheper, G.C., van der Knaap, M.S., and Gulati, S. (2011). Leukoencephalopathy with brain stem and spinal cord involvement and high lactate: a genetically proven case without elevated white matter lactate. *J Child Neurol* 26(6)**,** 773-776. doi: 10.1177/0883073810390695.

Simon, M., Richard, E.M., Wang, X., Shahzad, M., Huang, V.H., Qaiser, T.A., et al. (2015). Mutations of human NARS2, encoding the mitochondrial asparaginyl-tRNA synthetase, cause nonsyndromic deafness and Leigh syndrome. *PLoS Genet* 11(3)**,** e1005097. doi: 10.1371/journal.pgen.1005097.

Simons, C., Griffin, L.B., Helman, G., Golas, G., Pizzino, A., Bloom, M., et al. (2015). Loss-of-function alanyl-tRNA synthetase mutations cause an autosomal-recessive early-onset epileptic encephalopathy with persistent myelination defect. *Am J Hum Genet* 96(4)**,** 675-681. doi: 10.1016/j.ajhg.2015.02.012.

Skorvanek, M., Rektorova, I., Mandemakers, W., Wagner, M., Steinfeld, R., Orec, L., et al. (2022). WARS2 mutations cause dopa-responsive early-onset parkinsonism and progressive myoclonus ataxia. *Parkinsonism Relat Disord* 94**,** 54-61. doi: 10.1016/j.parkreldis.2021.11.030.

Smith, F., Hopton, S., Dallabona, C., Gilberti, M., Falkous, G., Norwood, F., et al. (2018). Sideroblastic anemia with myopathy secondary to novel, pathogenic missense variants in the YARS2 gene. *Haematologica* 103(12)**,** e564-e566. doi: 10.3324/haematol.2018.194464.

Sofou, K., Kollberg, G., Hedberg-Oldfors, C., and Oldfors, A. (2021). The phenotypic variability and natural history of NARS2 associated disease. *Eur J Paediatr Neurol* 31**,** 31-37. doi: 10.1016/j.ejpn.2021.01.012.

Sofou, K., Kollberg, G., Holmström, M., Dávila, M., Darin, N., Gustafsson, C.M., et al. (2015). Whole exome sequencing reveals mutations in NARS2 and PARS2, encoding the mitochondrial asparaginyl-tRNA synthetase and prolyl-tRNA synthetase, in patients with Alpers syndrome. *Mol Genet Genomic Med* 3(1)**,** 59-68. doi: 10.1002/mgg3.115.

Sofou, K., Moslemi, A.R., Kollberg, G., Bjarnadóttir, I., Oldfors, A., Nennesmo, I., et al. (2012). Phenotypic and genotypic variability in Alpers syndrome. *Eur J Paediatr Neurol* 16(4)**,** 379-389. doi: 10.1016/j.ejpn.2011.12.006.

Soldà, G., Caccia, S., Robusto, M., Chiereghin, C., Castorina, P., Ambrosetti, U., et al. (2016). First independent replication of the involvement of LARS2 in Perrault syndrome by whole-exome sequencing of an Italian family. *J Hum Genet* 61(4)**,** 295-300. doi: 10.1038/jhg.2015.149.

Sommerville, E.W., Ng, Y.S., Alston, C.L., Dallabona, C., Gilberti, M., He, L., et al. (2017). Clinical Features, Molecular Heterogeneity, and Prognostic Implications in YARS2-Related Mitochondrial Myopathy. *JAMA Neurol* 74(6)**,** 686-694. doi: 10.1001/jamaneurol.2016.4357.

Sommerville, E.W., Zhou, X.L., Oláhová, M., Jenkins, J., Euro, L., Konovalova, S., et al. (2019). Instability of the mitochondrial alanyl-tRNA synthetase underlies fatal infantile-onset cardiomyopathy. *Hum Mol Genet* 28(2)**,** 258-268. doi: 10.1093/hmg/ddy294.

Souissi, A., Ben Said, M., Frikha, F., Elloumi, I., Masmoudi, S., and Megarbane, A. (2021). Expanding the Clinical and Molecular Spectrum of HARS2-Perrault Syndrome: Identification of a Novel Homozygous Missense Variant in the HARS2 gene. *Genet Test Mol Biomarkers* 25(8)**,** 528-539. doi: 10.1089/gtmb.2021.0092.

Srivastava, S., Butala, A., Mahida, S., Richter, J., Mu, W., Poretti, A., et al. (2019). Expansion of the clinical spectrum associated with AARS2-related disorders. *Am J Med Genet A* 179(8)**,** 1556-1564. doi: 10.1002/ajmg.a.61188.

Steenweg, M.E., Ghezzi, D., Haack, T., Abbink, T.E., Martinelli, D., van Berkel, C.G., et al. (2012a). Leukoencephalopathy with thalamus and brainstem involvement and high lactate 'LTBL' caused by EARS2 mutations. *Brain* 135(Pt 5)**,** 1387-1394. doi: 10.1093/brain/aws070.

Steenweg, M.E., Pouwels, P.J., Wolf, N.I., van Wieringen, W.N., Barkhof, F., and van der Knaap, M.S. (2011). Leucoencephalopathy with brainstem and spinal cord involvement and high lactate: quantitative magnetic resonance imaging. *Brain* 134(Pt 11)**,** 3333-3341. doi: 10.1093/brain/awr254.

Steenweg, M.E., van Berge, L., van Berkel, C.G., de Coo, I.F., Temple, I.K., Brockmann, K., et al. (2012b). Early-onset LBSL: how severe does it get? *Neuropediatrics* 43(6)**,** 332-338. doi: 10.1055/s-0032-1329395.

Stellingwerff, M.D., Figuccia, S., Bellacchio, E., Alvarez, K., Castiglioni, C., Topaloglu, P., et al. (2021). LBSL: Case Series and DARS2 Variant Analysis in Early Severe Forms With Unexpected Presentations. *Neurol Genet* 7(2)**,** e559. doi: 10.1212/nxg.0000000000000559.

Štěrbová, K., Vlčková, M., Hansíková, H., Sebroňová, V., Sedláčková, L., Pavlíček, P., et al. (2021). Novel variants in the NARS2 gene as a cause of infantile-onset severe epilepsy leading to fatal refractory status epilepticus: case study and literature review. *Neurogenetics* 22(4)**,** 359-364. doi: 10.1007/s10048-021-00659-0.

Sun, J., Quan, C., Luo, S.S., Zhou, L., and Zhao, C.B. (2017). Leukodystrophy without Ovarian Failure Caused by Compound Heterozygous Alanyl-tRNA Synthetase 2 Mutations. *Chin Med J (Engl)* 130(24)**,** 3021-3022. doi: 10.4103/0366-6999.220300.

Sun, L., Lin, Z., Zhang, J., Shen, J., Wang, X., and Yang, J. (2022). Genetic etiological analysis of auditory neuropathy spectrum disorder by next-generation sequencing. *Front Neurol* 13**,** 1026695. doi: 10.3389/fneur.2022.1026695.

Synofzik, M., Schicks, J., Lindig, T., Biskup, S., Schmidt, T., Hansel, J., et al. (2011). Acetazolamide-responsive exercise-induced episodic ataxia associated with a novel homozygous DARS2 mutation. *J Med Genet* 48(10)**,** 713-715. doi: 10.1136/jmg.2011.090282.

Taglia, I., Di Donato, I., Bianchi, S., Cerase, A., Monti, L., Marconi, R., et al. (2018). AARS2-related ovarioleukodystrophy: Clinical and neuroimaging features of three new cases. *Acta Neurol Scand* 138(4)**,** 278-283. doi: 10.1111/ane.12954.

Takezawa, Y., Fujie, H., Kikuchi, A., Niihori, T., Funayama, R., Shirota, M., et al. (2018). Novel IARS2 mutations in Japanese siblings with CAGSSS, Leigh, and West syndrome. *Brain Dev* 40(10)**,** 934-938. doi: 10.1016/j.braindev.2018.06.010.

Tanaka, R., Takeguchi, R., Kuroda, M., Suzuki, N., Makita, Y., Yanagi, K., et al. (2022). Novel NARS2 variant causing leigh syndrome with normal lactate levels. *Hum Genome Var* 9(1)**,** 12. doi: 10.1038/s41439-022-00191-z.

Taskin, B.D., Karalok, Z.S., Gurkas, E., Aydin, K., Aydogmus, U., Ceylaner, S., et al. (2016). Early-Onset Mild Type Leukoencephalopathy Caused by a Homozygous EARS2 Mutation. *J Child Neurol* 31(7)**,** 938-941. doi: 10.1177/0883073816630087.

Taylor, R.W., Pyle, A., Griffin, H., Blakely, E.L., Duff, J., He, L., et al. (2014). Use of whole-exome sequencing to determine the genetic basis of multiple mitochondrial respiratory chain complex deficiencies. *Jama* 312(1)**,** 68-77. doi: 10.1001/jama.2014.7184.

Theisen, B.E., Rumyantseva, A., Cohen, J.S., Alcaraz, W.A., Shinde, D.N., Tang, S., et al. (2017). Deficiency of WARS2, encoding mitochondrial tryptophanyl tRNA synthetase, causes severe infantile onset leukoencephalopathy. *Am J Med Genet A* 173(9)**,** 2505-2510. doi: 10.1002/ajmg.a.38339.

Thiffault, I., Rioux, M.F., Tetreault, M., Jarry, J., Loiselle, L., Poirier, J., et al. (2006). A new autosomal recessive spastic ataxia associated with frequent white matter changes maps to 2q33-34. *Brain* 129(Pt 9)**,** 2332-2340. doi: 10.1093/brain/awl110.

Tucker, E.J., Rius, R., Jaillard, S., Bell, K., Lamont, P.J., Travessa, A., et al. (2020). Genomic sequencing highlights the diverse molecular causes of Perrault syndrome: a peroxisomal disorder (PEX6), metabolic disorders (CLPP, GGPS1), and mtDNA maintenance/translation disorders (LARS2, TFAM). *Hum Genet* 139(10)**,** 1325-1343. doi: 10.1007/s00439-020-02176-w.

Tylki-Szymanska, A., Jurkiewicz, E., Zakharova, E.Y., and Bobek-Billewicz, B. (2014). Leukoencephalopathy with brain stem and spinal cord involvement and lactate elevation: high outcome variation between two siblings. *Neuropediatrics* 45(3)**,** 188-191. doi: 10.1055/s-0033-1364105.

Tzoulis, C., Tran, G.T., Gjerde, I.O., Aasly, J., Neckelmann, G., Rydland, J., et al. (2012). Leukoencephalopathy with brainstem and spinal cord involvement caused by a novel mutation in the DARS2 gene. *J Neurol* 259(2)**,** 292-296. doi: 10.1007/s00415-011-6176-9.

Uluc, K., Baskan, O., Yildirim, K.A., Ozsahin, S., Koseoglu, M., Isak, B., et al. (2008). Leukoencephalopathy with brain stem and spinal cord involvement and high lactate: a genetically proven case with distinct MRI findings. *J Neurol Sci* 273(1-2)**,** 118-122. doi: 10.1016/j.jns.2008.06.002.

Upadia, J., Li, Y., Walano, N., Deputy, S., Gajewski, K., and Andersson, H.C. (2022). Genotype-phenotype correlation in IARS2-related diseases: A case report and review of literature. *Clin Case Rep* 10(2)**,** e05401. doi: 10.1002/ccr3.5401.

Vafaee-Shahi, M., Farhadi, M., Razmara, E., Morovvati, S., Ghasemi, S., Abedini, S.S., et al. (2022). Novel phenotype and genotype spectrum of NARS2 and literature review of previous mutations. *Ir J Med Sci* 191(4)**,** 1877-1890. doi: 10.1007/s11845-021-02736-7.

van Berge, L., Hamilton, E.M., Linnankivi, T., Uziel, G., Steenweg, M.E., Isohanni, P., et al. (2014). Leukoencephalopathy with brainstem and spinal cord involvement and lactate elevation: clinical and genetic characterization and target for therapy. *Brain* 137(Pt 4)**,** 1019-1029. doi: 10.1093/brain/awu026.

van der Knaap, M.S., Bugiani, M., Mendes, M.I., Riley, L.G., Smith, D.E.C., Rudinger-Thirion, J., et al. (2019). Biallelic variants in LARS2 and KARS cause deafness and (ovario)leukodystrophy. *Neurology* 92(11)**,** e1225-e1237. doi: 10.1212/wnl.0000000000007098.

van der Knaap, M.S., van der Voorn, P., Barkhof, F., Van Coster, R., Krägeloh-Mann, I., Feigenbaum, A., et al. (2003). A new leukoencephalopathy with brainstem and spinal cord involvement and high lactate. *Ann Neurol* 53(2)**,** 252-258. doi: 10.1002/ana.10456.

van Dijk, T., van Ruissen, F., Jaeger, B., Rodenburg, R.J., Tamminga, S., van Maarle, M., et al. (2017). RARS2 Mutations: Is Pontocerebellar Hypoplasia Type 6 a Mitochondrial Encephalopathy? *JIMD Rep* 33**,** 87-92. doi: 10.1007/8904_2016_584.

van Helden, R.W.J., Birket, M.J., Freund, C., Arendzen, C.H., Mikkers, H.M., Orlova, V., et al. (2021). Generation of three human induced pluripotent stem cell lines, LUMCi024-A, LUMCi025-A, and LUMCi026-A, from two patients with combined oxidative phosphorylation deficiency 8 and a related control. *Stem Cell Res* 53**,** 102374. doi: 10.1016/j.scr.2021.102374.

Vanlander, A.V., Menten, B., Smet, J., De Meirleir, L., Sante, T., De Paepe, B., et al. (2015). Two siblings with homozygous pathogenic splice-site variant in mitochondrial asparaginyl-tRNA synthetase (NARS2). *Hum Mutat* 36(2)**,** 222-231. doi: 10.1002/humu.22728.

Vantroys, E., Larson, A., Friederich, M., Knight, K., Swanson, M.A., Powell, C.A., et al. (2017). New insights into the phenotype of FARS2 deficiency. *Mol Genet Metab* 122(4)**,** 172-181. doi: 10.1016/j.ymgme.2017.10.004.

Vantroys, E., Smet, J., Vanlander, A.V., Vergult, S., De Bruyne, R., Roels, F., et al. (2018). Severe hepatopathy and neurological deterioration after start of valproate treatment in a 6-year-old child with mitochondrial tryptophanyl-tRNA synthetase deficiency. *Orphanet J Rare Dis* 13(1)**,** 80. doi: 10.1186/s13023-018-0822-6.

Vernon, H.J., McClellan, R., Batista, D.A., and Naidu, S. (2015). Mutations in FARS2 and non-fatal mitochondrial dysfunction in two siblings. *Am J Med Genet A* 167a(5)**,** 1147-1151. doi: 10.1002/ajmg.a.36993.

Ville, D., Lesca, G., Labalme, A., Portes, V.D., Arzimanoglou, A., and de Bellescize, J. (2020). Early-onset epileptic encephalopathy with migrating focal seizures associated with a FARS2 homozygous nonsense variant. *Epileptic Disord* 22(3)**,** 327-335. doi: 10.1684/epd.2020.1168.

Virdee, M., Swarnalingam, E., Kozenko, M., Tarnopolsky, M., and Jones, K. (2019). Expanding the Phenotype: Neurodevelopmental Disorder, Mitochondrial, With Abnormal Movements and Lactic Acidosis, With or Without Seizures (NEMMLAS) due to WARS2 Biallelic Variants, Encoding Mitochondrial Tryptophanyl-tRNA Synthase. *J Child Neurol* 34(12)**,** 778-781. doi: 10.1177/0883073819854604.

Vona, B., Maroofian, R., Bellacchio, E., Najafi, M., Thompson, K., Alahmad, A., et al. (2018). Expanding the clinical phenotype of IARS2-related mitochondrial disease. *BMC Med Genet* 19(1)**,** 196. doi: 10.1186/s12881-018-0709-3.

Waldron, A., Wilcox, C., Francklyn, C., and Ebert, A. (2019). Knock-Down of Histidyl-tRNA Synthetase Causes Cell Cycle Arrest and Apoptosis of Neuronal Progenitor Cells in vivo. *Front Cell Dev Biol* 7**,** 67. doi: 10.3389/fcell.2019.00067.

Walker, M.A., Mohler, K.P., Hopkins, K.W., Oakley, D.H., Sweetser, D.A., Ibba, M., et al. (2016). Novel Compound Heterozygous Mutations Expand the Recognized Phenotypes of FARS2-Linked Disease. *J Child Neurol* 31(9)**,** 1127-1137. doi: 10.1177/0883073816643402.

Wang, F., Huang, G.D., Tian, H., Zhong, Y.B., Shi, H.J., Li, Z., et al. (2015). Point mutations in KAL1 and the mitochondrial gene MT-tRNA(cys) synergize to produce Kallmann syndrome phenotype. *Sci Rep* 5**,** 13050. doi: 10.1038/srep13050.

Wang, J.Y., Chen, S.F., Zhang, H.Q., Wang, M.Y., Zhu, J.H., and Zhang, X. (2019). A homozygous mutation of alanyl-transfer RNA synthetase 2 in a patient of adult-onset leukodystrophy: A case report and literature review. *Brain Behav* 9(7)**,** e01313. doi: 10.1002/brb3.1313.

Wang, M., Sips, P., Khin, E., Rotival, M., Sun, X., Ahmed, R., et al. (2016). Wars2 is a determinant of angiogenesis. *Nat Commun* 7**,** 12061. doi: 10.1038/ncomms12061.

Webb, B.D., Wheeler, P.G., Hagen, J.J., Cohen, N., Linderman, M.D., Diaz, G.A., et al. (2015). Novel, compound heterozygous, single-nucleotide variants in MARS2 associated with developmental delay, poor growth, and sensorineural hearing loss. *Hum Mutat* 36(6)**,** 587-592. doi: 10.1002/humu.22781.

Wongkittichote, P., Magistrati, M., Shimony, J.S., Smyser, C.D., Fatemi, S.A., Fine, A.S., et al. (2022). Functional analysis of missense DARS2 variants in siblings with leukoencephalopathy with brain stem and spinal cord involvement and lactate elevation. *Mol Genet Metab* 136(4)**,** 260-267. doi: 10.1016/j.ymgme.2022.07.002.

Wortmann, S.B., Timal, S., Venselaar, H., Wintjes, L.T., Kopajtich, R., Feichtinger, R.G., et al. (2017). Biallelic variants in WARS2 encoding mitochondrial tryptophanyl-tRNA synthase in six individuals with mitochondrial encephalopathy. *Hum Mutat* 38(12)**,** 1786-1795. doi: 10.1002/humu.23340.

Wu, X.H., Lin, S.Z., Zhou, Y.Q., Wang, W.Q., Li, J.Y., and Chen, Q.D. (2022). VARS2 gene mutation leading to overall developmental delay in a child with epilepsy: A case report. *World J Clin Cases* 10(24)**,** 8749-8754. doi: 10.12998/wjcc.v10.i24.8749.

Xu, P., Wang, L., Peng, H., Liu, H., Liu, H., Yuan, Q., et al. (2021). Disruption of Hars2 in Cochlear Hair Cells Causes Progressive Mitochondrial Dysfunction and Hearing Loss in Mice. *Front Cell Neurosci* 15**,** 804345. doi: 10.3389/fncel.2021.804345.

Xu, Y., Wu, B.B., Wang, H.J., Zhou, S.Z., Cheng, G.Q., and Zhou, Y.F. (2020). A term neonate with early myoclonic encephalopathy caused by RARS2 gene variants: a case report. *Transl Pediatr* 9(5)**,** 707-712. doi: 10.21037/tp-20-110.

Yagasaki, H., Sano, F., Narusawa, H., Watanabe, D., Kaga, Y., Kobayashi, K., et al. (2022). Compound heterozygous variants of the NARS2 gene in siblings with developmental delay, epilepsy, and neonatal diabetes syndrome. *Am J Med Genet A* 188(8)**,** 2466-2471. doi: 10.1002/ajmg.a.62873.

Yahia, A., Elsayed, L., Babai, A., Salih, M.A., El-Sadig, S.M., Amin, M., et al. (2018). Intra-familial phenotypic heterogeneity in a Sudanese family with DARS2-related leukoencephalopathy, brainstem and spinal cord involvement and lactate elevation: a case report. *BMC Neurol* 18(1)**,** 175. doi: 10.1186/s12883-018-1180-7.

Yamashita, S., Miyake, N., Matsumoto, N., Osaka, H., Iai, M., Aida, N., et al. (2013). Neuropathology of leukoencephalopathy with brainstem and spinal cord involvement and high lactate caused by a homozygous mutation of DARS2. *Brain Dev* 35(4)**,** 312-316. doi: 10.1016/j.braindev.2012.05.007.

Yang, F., Wang, D., Zhang, X., Fan, H., Zheng, Y., Xiao, Z., et al. (2022a). Novel variants of seryl-tRNA synthetase resulting in HUPRA syndrome featured in pulmonary hypertension. *Front Cardiovasc Med* 9**,** 1058569. doi: 10.3389/fcvm.2022.1058569.

Yang, Y., Liu, W., Fang, Z., Shi, J., Che, F., He, C., et al. (2016). A Newly Identified Missense Mutation in FARS2 Causes Autosomal-Recessive Spastic Paraplegia. *Hum Mutat* 37(2)**,** 165-169. doi: 10.1002/humu.22930.

Yang, Z., Cao, J., Song, Y., Li, S., Jiao, Z., Ren, S., et al. (2022b). Whole-exome sequencing identified novel variants in three Chinese Leigh syndrome pedigrees. *Am J Med Genet A* 188(4)**,** 1214-1225. doi: 10.1002/ajmg.a.62641.

Yazici Gencdal, I., Dincer, A., Obuz, O., and Yapici, Z. (2020). Leukoencephalopathy With Brain Stem and Spinal Cord Involvement and Lactate Elevation (LBSL): A Case With Long-term Follow-up. *Neurologist* 25(5)**,** 144-147. doi: 10.1097/nrl.0000000000000286.

Yelam, A., Nagarajan, E., Chuquilin, M., and Govindarajan, R. (2019). Leucoencephalopathy with brain stem and spinal cord involvement and lactate elevation: a novel mutation in the DARS2 gene. *BMJ Case Rep* 12(1). doi: 10.1136/bcr-2018-227755.

Yin, X., Tang, B., Mao, X., Peng, J., Zeng, S., Wang, Y., et al. (2018). The genotypic and phenotypic spectrum of PARS2-related infantile-onset encephalopathy. *J Hum Genet* 63(9)**,** 971-980. doi: 10.1038/s10038-018-0478-z.

Yu, J., Jiang, W., Cao, L., Na, X., and Yang, J. (2020). Two novel likely pathogenic variants of HARS2 identified in a Chinese family with sensorineural hearing loss. *Hereditas* 157(1)**,** 47. doi: 10.1186/s41065-020-00157-7.

Yu, T., Zhang, Y., Zheng, W.Q., Wu, S., Li, G., Zhang, Y., et al. (2022). Selective degradation of tRNASer(AGY) is the primary driver for mitochondrial seryl-tRNA synthetase-related disease. *Nucleic Acids Res*. doi: 10.1093/nar/gkac1028.

Zhang, J., Zhang, Z., Zhang, Y., and Wu, Y. (2018). Distinct magnetic resonance imaging features in a patient with novel RARS2 mutations: A case report and review of the literature. *Exp Ther Med* 15(1)**,** 1099-1104. doi: 10.3892/etm.2017.5491.

Zhang, Y., Yu, Y., Zhao, X., Xu, Y., Chen, L., Li, N., et al. (2022). Novel RARS2 Variants: Updating the Diagnosis and Pathogenesis of Pontocerebellar Hypoplasia Type 6. *Pediatr Neurol* 131**,** 30-41. doi: 10.1016/j.pediatrneurol.2022.04.002.

Zheng, T., Luo, Q., Han, C., Zhou, J., Gong, J., Chun, L., et al. (2022a). Cytoplasmic and mitochondrial aminoacyl-tRNA synthetases differentially regulate lifespan in Caenorhabditis elegans. *iScience* 25(11)**,** 105266. doi: 10.1016/j.isci.2022.105266.

Zheng, W.Q., Pedersen, S.V., Thompson, K., Bellacchio, E., French, C.E., Munro, B., et al. (2022b). Elucidating the molecular mechanisms associated with TARS2-related mitochondrial disease. *Hum Mol Genet* 31(4)**,** 523-534. doi: 10.1093/hmg/ddab257.

Zhou, Y., Chen, B., Li, L., Pan, H., Liu, B., Li, T., et al. (2019). Novel alanyl-tRNA synthetase 2 (AARS2) homozygous mutation in a consanguineous Chinese family with premature ovarian insufficiency. *Fertil Steril* 112(3)**,** 569-576.e562. doi: 10.1016/j.fertnstert.2019.05.005.

Zhou, Y., Zhong, C., Yang, Q., Zhang, G., Yang, H., Li, Q., et al. (2021). Novel SARS2 variants identified in a Chinese girl with HUPRA syndrome. *Mol Genet Genomic Med* 9(4)**,** e1650. doi: 10.1002/mgg3.1650.

Zou, S., Mei, X., Yang, W., Zhu, R., Yang, T., and Hu, H. (2020). Whole-exome sequencing identifies rare pathogenic and candidate variants in sporadic Chinese Han deaf patients. *Clin Genet* 97(2)**,** 352-356. doi: 10.1111/cge.13638.
